# Microsomal Omega-3 Fatty Acid Desaturase Genes in Low Linolenic Acid Soybean Line RG10 and Validation of Major Linolenic Acid QTL

**DOI:** 10.3389/fgene.2016.00038

**Published:** 2016-03-29

**Authors:** Yarmilla Reinprecht, K. Peter Pauls

**Affiliations:** Department of Plant Agriculture, University of GuelphGuelph, ON, Canada

**Keywords:** soybean, ω-3 fatty acid desaturase, *FAD3* genes, QTL validation, gene-based markers, *in silico*, synteny

## Abstract

High levels of linolenic acid (80 g kg^−1^) are associated with the development of off-flavors and poor stability in soybean oil. The development of low linolenic acid lines such as RG10 (20 g kg^−1^ linolenic acid) can reduce these problems. The level of linolenic acid in seed oil is determined by the activities of microsomal omega-3 fatty acid desaturases (FAD3). A major linolenic acid QTL (>70% of variation) on linkage group B2 (chromosome Gm14) was previously detected in a recombinant inbred line population from the RG10 × OX948 cross. The objectives of this study were to validate the major linolenic acid QTL in an independent population and characterize all the soybean *FAD3* genes. Four *FAD3* genes were sequenced and localized in RG10 and OX948 and compared to the genes in the reference Williams 82 genome. The *FAD3A* gene sequences mapped to the locus Glyma.14g194300 [on the chromosome Gm14 (B2)], which is syntenic to the *FAD3B* gene (locus Glyma.02g227200) on the chromosome Gm02 (D1b). The location of the *FAD3A* gene is the same as was previously determined for the *fan* allele, that conditions low linolenic acid content and several linolenic acid QTL, including Linolen 3-3, mapped previously with the RG10 × OX948 population and confirmed in the PI 361088B × OX948 population as Linolen-PO (FAD3A). The *FAD3B* gene-based marker, developed previously, was mapped to the chromosome Gm02 (D1b) in a region containing a newly detected linolenic acid QTL [Linolen-RO(FAD3B)] in the RG10 × OX948 genetic map and corresponds well with the *in silico* position of the *FAD3B* gene sequences. *FAD3C* and *FAD3D* gene sequences, mapped to syntenic regions on chromosomes Gm18 (locus Glyma.18g062000) and Gm11 (locus Glyma.11g227200), respectively. Association of linolenic acid QTL with the desaturase genes *FAD3A* and *FAD3*B, their validation in an independent population, and development of *FAD3* gene-specific markers should simplify and accelerate breeding for low linolenic acid soybean cultivars.

## Introduction

Soybean is grown primarily for high content of oil (20 g kg^−1^) and protein (40 g kg^−1^) in seed. Oil extracted from standard soybean cultivars contains approximately 110 g kg^−1^ palmitic acid (16:0), 50 g kg^−1^ stearic acid (18:0), 210 g kg^−1^ oleic acid (18:1), 550 g kg^−1^ linoleic acid (18:2), and 80 g kg^−1^ linolenic acid (18:3) (Wilcox, 1989). Although a source of omega fatty acid from plants, the normal level of linolenic acid in standard cultivars is associated with instability and short shelf life of soybean oil. Oxidation of linolenic acid results in the accumulation of undesirable odors and flavors in the oil, which reduces its palatability and food quality (Wilson et al., 1981; Kinney, 1997; Reinprecht et al., 2011). Soybean oil stability problems can be reduced by developing cultivars with low linolenic acid levels (Hammond and Fehr, 1975). For good flavor stability and increased storage life, the linolenic acid content of soybean seed should be 10 g kg^−1^ or less (Khanna and Singh, 1991). Oil extracted from low linolenic acid lines is more stable, needs less hydrogenation and contains lower levels of *trans* fatty acids than oil extracted from conventional soybean cultivars (Mounts et al., 1986; Clemente and Cahoon, 2009).

Fatty acid biosynthesis in plants is under developmental and tissue-specific control (Guerra and Holbrook, 1988). *De novo* fatty acid biosynthesis occurs in the chloroplast stroma of leaves and proplastids of seeds and their incorporation into triacylglycerides (TAGs) takes place in the endoplasmic reticulum (ER; Browse and Somerville, 1991; Ohlrogge et al., 1993). In higher plants, polyunsaturated fatty acids (PUFA) are synthesized through both prokaryotic and eukaryotic pathways (Browse et al., 1986). The two pathways are encoded by separate sets of genes (Harwood, 1988; Somerville and Browse, 1991).

In soybean seed, linolenic acid is synthesized by consecutive desaturation [the creation of a double bond (at the delta position) by the enzymatic removal of hydrogen from a methylene group in an acyl chain (Shanklin and Cahoon, 1998)] of oleic acid catalyzed by fatty acid desaturases (FADs, Cherif et al., 1975). Thus, two sets of genes might control linolenic acid content, which may act in additive, possibly sequential ways. The *FAD2* genes encode omega (ω)-6 FADs and control the conversion of oleic acid into linoleic acid. The *FAD3* gene family encodes ω-3 FADs, which catalyzes transformation of linoleic acid into linolenic acid (Yadav et al., 1993).

The FADs possess common structural features and likely share a catalytic mechanism. Almost all membrane-bound FADs are characterized by the presence of a di-iron cofactor that interacts with three regions of conserved histidine motifs (**H**X_(3−4)_**H**X_(7−41)_**H**X_(2−3)_**HH**X_(61−189)_**H**X_(2−3)_**HH**), which are essential for catalytic activity (Shanklin et al., 1994). In addition, the amino acid sequence FVLG**H**DCG**H**GSF (which includes histidine box 1) is found in all known plant omega (ω)-3 FADs (Yadav et al., 1993).

The level of linolenic acid in soybean seed is determined by three *FAD3* genes (*FAD3A, FAD3B*, and *FAD3C;* Bilyeu et al., 2003). At least three major loci (*Fan, Fan2*, and *Fan3*) and potentially a fourth (*Fanx*), have been identified with multiple alleles, including some with the reduced levels of linolenic acid (Rajcan et al., 2005; Lee et al., 2007). The *Fan* locus was mapped between the RFLP markers B124_1 and B194_1 on the linkage group (LG) B2 or chromosome Gm14 (Brummer et al., 1995). The first *FAD3* gene was mapped to chromosome Gm14 (or linkage group B2, Byrum et al., 1995). A reduced level of linolenic acid in A5 was associated with full or partial deletion of a microsomal omega-3 desaturase gene (Byrum et al., 1995).

Loci associated with the low levels of linolnic acid in soybean have been linked to the genes encoding FAD3 desaturases and the annotated genome sequences (Bilyeu et al., 2011). *FAD3A* (*FAD3-1b*) gene corresponds to the *Fan* locus and, in the current Wm82.a2.v1 annotation of soybean genome sequence (Phytozome, http://phytozome.jgi.doe.gov/pz/portal.html; Goodstein et al., 2012), is represented as Glyma.14g194300 on the chromosome G14 (B2) (Glyma.14g194300/*FAD3A/Fan*). *FAD3B* (*FAD3-1a*) gene is analogous to the *Fan3* locus and is represented as Glyma.02g227200 on the chromosome Gm02 (D1b) (Glyma.02g227200/*FAD3B/Fan3*). *FAD3C* (*FAD3-2a*) gene is the *Fan2* locus and is represented as Glyma.18g062000 on the chromosome Gm18 (G) (Glyma.18g062000/*FAD3C*/*Fan2*). *FAD3D* (*FAD3-2b*) gene is at the locus Glyma.11g174100 on the chromosome Gm11 (B1). However, more work is needed to assign Glyma.11g174100/*FAD3D (FAD3-2b)* to any locus associated with the linolenic acid levels.

The *FAD3* gene sequences were determined and deposited in GenBank (Bilyeu et al., 2003; Anai et al., 2005). They have similar structures and contain eight exons (Iba et al., 1993; Chi et al., 2011). The recent release of complete draft of the soybean genome sequence gives new insights into complex organization of this ancient paleopolyploid (Schmutz et al., 2010). Four regions containing *FAD3* genes were identified in soybean genome (Chi et al., 2011; Gilman and Bilyeu, 2012).

The genetic basis of low linolenic acid soybean was associated with mutations in one or more *FAD3* genes (Bilyeu et al., 2003, 2005, 2006; Anai et al., 2005; Chappell and Bilyeu, 2006, 2007; Reinprecht et al., 2009). For example, PI 361088B is a naturally-occurring mutant line which contains over 4% seed linolenic acid (Rennie et al., 1988). A two nucleotide insertion in the coding sequence of *FAD3A* gene resulted in a frameshift and a premature stop codon (Chappell and Bilyeu, 2007). The 3% of linolenic acid content in the seed oil of the line 10–73 (derived from CX1512- 44) was associated with mutations in *FAD3A* and *FAD3C* genes (Bilyeu et al., 2005). Low linolenic acid line RG10, also contains less than 3% linolenic acid. The line was developed by remutagenesis (Stojšin et al., 1998) of the 4% linolenic acid line C1640 (Wilcox and Cavins, 1985). In addition to the *FAD3A* mutation from C1640 (Chappell and Bilyeu, 2006), RG10 contains a novel null allele of the *FAD3B* gene (Reinprecht et al., 2009). By combining mutations in all three *FAD3* genes from an RG10-derived soybean line (RCAT 0716L, *GmFAD3aabbCC*) and 10–73 (*GmFAD3aaBBcc*), Bilyeu et al. (2011) were able to produce novel soybean lines (*GmFAD3aabbcc*) containing only 1% linolenic acid in the seed oil.

The current reference soybean (GmComposite_2003) genetic map [created using the 1999 ISU/USDA (Iowa State University/United States Department of Agriculture) map as the foundation] covers 2657.93 cM of Kosambi map distance and contains 6394 markers and 3027 QTL on 20 linkage groups (LGs) or chromosomes [SoyBase (http://soybase.org/; accessed 26 Sep 2015); Grant et al., 2010]. Forty four QTL for linolenic acid were identified on 14 LGs (chromosomes) of the GmComposite_2003 map by 10 research groups.

We developed a molecular linkage map with 118 markers and identified QTL for linolenic acid content and seed lipoxygenases based on the RG10 × OX948 recombinant inbred line (RIL) population. Based on composite interval mapping (CIM), a major linolenic acid QTL (Linolen 3-3) that accounted for 72–78% of linolenic acid variability, was positioned on LG B2 (Gm14, flanked by SSR marker Satt534 and *FAD3* gene-specific marker FAD3i6; Reinprecht et al., 2006; SoyBase). Although strongly expressed in all three Ontario locations (Harrow, Ridgetown, and Woodslee in 2000) this QTL needed to be validated in different genetic backgrounds in order to be useful for marker-assisted selection (MAS) of soybean lines with reduced levels of linolenic acid. QTL regions are generally large and can contain thousands of potential candidate genes for the trait. The most robust marker for a QTL is located in the gene that determines the phenotype. In our previous work, *FAD3A* gene-based markers were mapped to the region of the major linolenic acid QTL on the chromosome Gm14 (B2) in the RG10 × OX948 population (Reinprecht et al., 2009).

The objectives of the current study were to characterize all of the *FAD3* genes in low linolenic acid genotype RG10 and a wild-type linolenic acid line (OX948) and to validate the major linolenic acid QTL (Linolen 3-3) in an independent PI 361088B × OX948 population. In a previous study (Reinprecht et al., 2009), conducted to determine molecular basis of low linolenic acid trait in RG10, we developed *FAD3A* and *FAD3B* gene mutation-based markers. In this work, we developed markers for the other two *FAD3* genes (*FAD3C* and *FAD3D*). The development of *FAD3* gene-based markers provides simple, genotype-based selection assays that can be used in early stages of breeding for soybean with low levels of linolenic acid.

## Materials and methods

### Plant material

Reciprocal crosses were made between the low linolenic acid mutant lines (RG10 and PI 361088B) and OX948 (seed lipoxygenase triple null line). PI 361088B is a naturally-occurring low linolenic acid source (Rennie et al., 1988) and line RG10 was developed at the University of Guelph, Ridgetown Campus (Stojšin et al., 1998) by treating the low linolenic acid line C1640 with ethyl methanesulfonate (EMS). The allele at the *Fan* locus in RG10 is designated as *fan*-b, for a very low level of linolenic acid (<25 g kg^−1^). In line PI 361088B the *Fan* allele is designated as *fan* (PI 361088B) for low level of linolenic acid (~39 g kg^−1^) (Rennie et al., 1988). OX948 is a selection from a backcross between Harovinton and a triple lipoxygenase null (3lx) F_2_ plant, and has high linolenic acid content (>80 g kg^−1^). The 3lx genotype, produced by gamma irradiation, was obtained from Dr. Kitamura (National Agricultural Research Centre, Ministry of Agriculture, Forestry and Fisheries, Tsukuba, Japan).

Detailed descriptions of the development of the RIL populations were presented in Reinprecht et al. (2005). The RG10 × OX948 mapping population of 169 F_5_-derived RILs was used to develop a linkage map and identify QTL for a number of seed and agronomic traits, including the content of linolenic acid (Reinprecht et al., 2006). Single seed descent was used to advance the F_3_ seed to the F_4_ generation in Belize (Continuous Crop Improvement Co., Ltd., Belize City), where harsh conditions (hurricane) resulted in a significant reduction in the number of lines in the PI 361088B × OX948 confirmation population; from 300 F_2_ lines to 45 F_5_-derived RILs.

### Determination of linolenic acid content and activity of ω-3 FAD

Fatty acid composition was determined using a half-seed technique (Wilcox and Cavins, 1985). Approximately one third of the cotyledon tissue distal from the embryonic axis was used for fatty acid analyses and the rest of the seed containing the embryo was planted for the next generation. The fatty acid compositions of the seed samples (obtained by bulking 10 half-seeds per parental lines and RILs) were determined by a gas liquid chromatography of fatty acid methyl esters (g kg^−1^) according to the modified method of Bannon et al. (1987) as described in Reinprecht et al. (2005). The estimates of ω-3 FAD activity (%) were derived from measurements of linoleic and linolenic acids levels (Cherif et al., 1975) as follows: 18:2 (ω-3) Desaturation = [(18:3)/(18:2 + 18:3)] × 100. The content of fatty acids and the level of ω-3 desaturation were analyzed using Proc Mix protocol in SAS Institute Inc (2009).

### Sequencing *FAD3* genes in RG10 and OX948

PCR amplification and sequencing of the *FAD3* genes are described in Reinprecht et al. (2009). Briefly, sequences for soybean seed *FAD3* genes, available in public databases at the initiation of this study [*G. max* microsomal ω-3 FAD mRNAs for *FAD3A* (GenBank accession AY204710), *FAD*3*B* (GenBank accession AY204711), *FAD*3*C* (GenBank accession AY204712), reported by Bilyeu et al. (2003) and *FAD3D* (*FAD3-2b*) (GenBank accession AB188198, Anai et al., 2005)], were aligned with the *Arabidopsis thaliana* microsomal *FAD3* gene (GenBank accession D26508) sequence (Nishiuchi et al., 1994) using the multiple sequence alignment program ClustalW (Chenna et al., 2003), at the European Bioinformatic Institute (EBI, http://www.ebi.ac.uk/clustalw). Based on differences among the alignments, primers to specifically amplify six to nine overlapping fragments from each of the four *FAD3* genes were designed [using Primer3 (Rozen and Skaletsky, 2000) and synthesized by Sigma-Aldrich Canada (Oakville, ON, Canada)]. In addition, the 1 kb flanking sequences of *FAD3A* and *FAD3B*, and 2 kb of *FAD3C* and *FAD3D* were used to amplify the 5′ and 3′ untranslated regions (UTR) in *FAD3* genes. The criteria for primer design included a length of 18 to 34 base pairs (bp), a GC content of 40 to 60%, fewer than four contiguous identical bases, and a melting temperature between 55 and 65°C. The *FAD3* gene-specific primers used to amplify overlapping fragments for sequencing are listed in a Table S1.

The gene-based primers were used in PCR reactions with genomic DNA from RG10 (low linolenic acid line) and OX948 (wild-type line) to amplify *FAD3* genes (*FAD3A*—6027 bp, region Gm14:45,934,715.45,940,741; FAD3B—6082 bp, region Gm02:41,418,800.41,424,881; *FAD3C*—6887 bp, region Gm18:5,644,513.5,651,399; and *FAD3D*—7372 bp, region Gm11:19,007,492.19,014,883 in the current soybean Wm82.a2.v1 genome annotation). DNA was isolated from the young, freeze-dried leaf tissue in a 3% CTAB solution using the method of Doyle and Doyle (1990). PCR reactions were performed in a total volume of 20 μl containing 1 × PCR buffer (supplied with enzyme), 3 mM MgCl_2_ (supplied with enzyme), 0.1 mM each of dNTPs (GE Healthcare Bio-Sciences Corp., Piscataway, NJ), 1.6 U *Taq* DNA Polymerase (Invitrogen Canada Inc., Burlington, ON, Canada), 5 μM each of the forward and reverse primer and 25 ng of soybean genomic DNA. The reaction mixture was amplified with a PTC-100™ Programmable Thermal Controller (MJ Research, Inc., Watertown, MA, USA) using the program that consisted of an initial 2 min denaturation step at 94°C, followed by 35 cycles of denaturation at 94°C for 30 s, annealing at 55–65°C for 45 s and extension at 72°C for 1 min, with a final extension at 72°C for 10 min. The PCR products were run on a 1% (w/v) agarose gel containing ethidium bromide in 1 × TBE buffer for 2 h at 100 V and visualized under UV light.

RG10 and OX948 PCR fragments were gel-purified and cloned (TOPO cloning kit, Invitrogen, according to the manufacturer's instructions) or used directly as template for cycle sequencing (CEQ™ 8000 genetic analysis system; Beckman Coulter Inc., Fullerton, CA, USA). After verification [BLAST searches at the National Center for Biotechnology Information (NCBI, http://www.ncbi.nlm.nih.gov/BLAST/) and Phytozome (http://phytozome.jgi.doe.gov/pz/portal.html#!search?show=BLAST)], sequences were assembled with the CAP3 program (Xiaoqiu and Madan, 1999). The exon/intron structure, initially determined by aligning genomic sequences with the available cDNA sequences, was confirmed with the reference sequences (NCBI and Phytozome). The gene structure was also analyzed with the FGenesh 2.6 (http://linux1.softberry.com; Salamov and Solovyev, 2000). Translation of nucleotide sequences was generated on ExPASy [Swiss Institute of Bioinformatics (SIB) Bioinformatics Resource Portal], http://www.expasy.ch/tools/dna.html). Verified RG10 and OX948 sequences of the *FAD3A, FAD3B, FAD3C*, and *FAD3D* genes were submitted to GenBank under accession numbers KU310958-KU310965.

### Development of molecular markers for *FAD3C* and *FAD3D* genes

Polymorphisms among *FAD3* genes from different sources were determined by comparing the mutant RG10 with a wild-type OX948 sequences and the wild-type Williams82 (Wm82) reference sequences. Sequence differences between RG10 and OX948 were used to design primers, develop molecular markers, and map *FAD3*C and *FAD3D* genes.

Because of the high similarity, *FAD3C* and *FAD3D* gene sequences were aligned and unique PCR primers were designed for both genes (*FAD3C* gene: forward 5′-TGA ATAAGCTGGCTTAGAAGTCAA-3′ and reverse 5′-**C**TTTCA AAGATTTATTTACTACATTCTAAA-3′; *FAD3D* gene: forward 5′-CCGGCGAGTGTCTTATGAACG-3′ and reverse 5′-CACT TGGTATCCCAACCTTCGAG-3′). The PCR mixture, PCR program (except for the annealing step, which was performed at 63°C for the FAD3C SNP and at 60°C for the FAD3D marker) and electrophoresis conditions were same as for the *FAD3* genes sequencing. Polymorphic primers were screened with the 169 F_5_ RILs from the mapping RG10 × OX948 population and 45 RILs from the verifying PI 361088B × OX948 population.

### *FAD3* genes mapping

#### *In silico* mapping and synteny analysis

The RG10 and OX948 *FAD3* gene sequences were BLASTed against the soybean Wm82.a2.v1 genome sequence (Phytozome). They mapped to four chromosomes (Gm02, Gm11, Gm14, and Gm18) and the sequence coordinates [the beginning and end positions (in bp) on the chromosome] were retrieved. The start positions for each gene were used to map (*in silico*) RG10 and OX948 *FAD3* gene sequences on the soybean sequence map. In addition, genome sequence (Wm82.a2.v1) coordinates for the Consensus map 4.0 markers on the chromosomes Gm14, Gm02, Gm18, and Gm11 were retrieved from SoyBase. Coordinates for the missing markers were obtained by BLASTing their sequences (indicated by “s” in marker name; SoyBase) against the soybean genome (Phytozome). Genetic maps (GmComposite_2003) coordinates for all features on these four chromosomes were downloaded from SoyBase. In this study, only QTL for linolenic acid were used.

Syntenic regions containing *FAD3* genes in soybean genome were determined using locus identifier (Glyma.14g194300, Glyma.02g227200, Glyma.18g062000, and Glyma.11g174100) in PGDD (Plant Genome Duplication Database, http://chibba.agtec.uga.edu/duplication/index/locus; Lee et al., 2013) against complete genome sequences available for 47 flowering plant species. Synteny analysis was also performed in CoGe's (Comparative Genomics) GEvo (Genome Evolution Analysis tool) using BlastZ alignment algorithm and a 40 Mb window (https://genomevolution.org/CoGe/GEvo.pl; Lyons and Freeling, 2008; Lyons et al., 2008).

#### Genotyping with SSRs

To connect different populations and various maps, SSR markers in *FAD3* gene regions on chromosomes Gm02 (D1b), Gm11 (B1), Gm14 (B2), and Gm18 (G) (based on *in silico* map data) were selected from the GmComposite_2003 map. Primers for the SSR markers were screened with parental genomic DNA (RG10, PI 361088B, and OX948) for polymorphisms in PCR reaction mixtures that were identical to those used for sequencing of *FAD3* genes. The SSR amplification program consisted of an initial denaturation for 2 min at 94°C, followed by 35 cycles with denaturation at 94°C, annealing at 48°C and extension at 68°C, each for 45 s with a final extension for 5 min at 72°C. The PCR products were separated by electrophoresis on 3% (w/v) super fine resolution agarose (Amresco, Solon, OH, USA) containing ethidium bromide in a 1 × TBE buffer at 100 V for 3 h and visualized under UV light. Polymorphic markers were used with DNA samples from 169 RILs from the RG10 × OX948 mapping population and 45 RILs from a validating population obtained from the PI 361088B × OX948 cross.

#### Linkage analysis

The new SSR and *FAD3* gene-specific markers were added to the regions containing the *FAD3* genes in the existing RG10 × OX948 map (Reinprecht et al., 2006) and used to develop a linkage map for the validating PI 361088B × OX948 population. Linkage analysis was performed using Mapmaker/EXP version 3.0b (Lander et al., 1987) with a minimum LOD score of 3.0 and maximum distance between two markers of 50.0 cM. Recombination frequencies were converted to cM distances using Kosambi's mapping function (Kosambi, 1944).

#### QTL analysis

QTL analysis was performed with composite interval mapping (CIM). The QTL number, their chromosomal locations and allelic effects were determined by CIM using QTL Cartographer version 2.5 (Wang et al., 2011) with forward and backward regression model (map function Kosambi, window size of 10.0 cM, five control markers and speed walk of 2.0 cM). QTL were declared significant if their LOD threshold values were ≥2.0. The location of a QTL was identified by the position of the peak in the significant LOD interval. The allelic effects at each significant QTL and the percentages of the phenotypic variation (*R*^2^) accounted by the identified QTL were obtained directly from the CIM output. Because the RIL populations were in the F_5_ generation only additive effects were considered. The locations and effects of the linolenic acid QTL identified in the current study were compared with fatty acid QTL previously mapped to the GmComposite_2003 map (SoyBase).

#### Maps alignments

Using common SSRs as anchors, the RG10 × OX948 linkage map was compared with the PI 361088B × OX948 map, as well as the GmComposite_2003 (SoyBase) genetic and sequence maps (Phytozome). On the sequence map, markers associated with the linolenic acid QTL were indicated with the bracketed QTL name (SoyBase and/or original publication). Maps were drawn in MapChart (Voorrips, 2002). Sequence and genetic maps containing *FAD3* (*FAD3A, FAD3B, FAD3C*, and *FAD3D*) genes were connected manually using shared (in general, classical SSR) markers. Naming of maps was based on soybean chromosome number to linkage group assignment (SoyBase).

## Results

### Linolenic acid content and ω-3 FAD activity in parental lines

**Figure 1:**
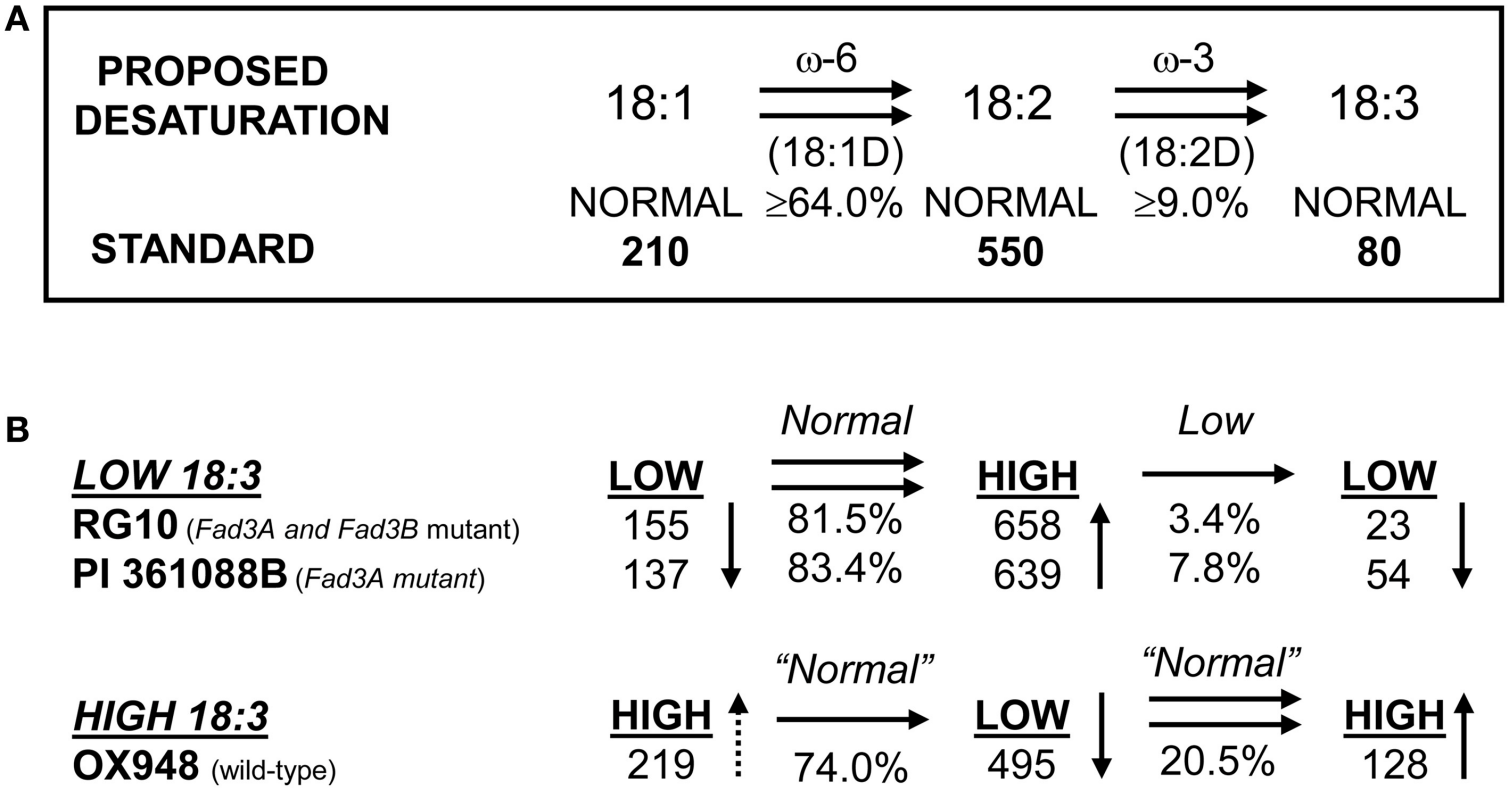
**Proposed mechanism of fatty acid desaturation in seeds of soybean lines RG10 and OX948**. **(A)** Desaturation steps after Wilson et al. (1990); Wilson (1991); **(B)** Desaturation steps proposed for parental lines RG10 (EMS *FAD3A* and *FAD3B* genes mutant:), PI 361088B (naturally occurring *FAD3A* gene mutant), and OX948 (wild-type).

The parental lines RG10, PI 361088B, and OX948 had significantly different levels of unsaturated fatty acids and differed in their relative ω-3 (18:2) desaturase activities (Table 1). In particular, RG10 contained 23 g kg^−1^, PI 361088B 54 g kg^−1^, and OX948 128 g kg^−1^ linolenic acid. The mechanisms of fatty acid desaturation proposed for the parental lines and the differences in the rate of ω-3 FAD activities are presented in Figure 1. Based on this model, the low levels of linolenic acid in RG10 and PI 361088B are results of low activities of the relative ω-3 fatty acid desaturation (3.4 and 7.8%, respectively) in seeds of these genotypes. The much higher level of linolenic acid in a wild-type line (OX948) is a result of 20.5% relative ω-3 FAD activity in this line (Figure 1).

**Table 1:** **Fatty acid composition and relative ω-3 fatty acid desaturase activity of soybean parental lines RG10, PI 361088B, and OX948**.

**Line**	**Fatty acid composition (g kg**^**−1**^**)**^**a**^					**ω-3 desaturation (%)^b^**
	**16:0**	**18:0**	**18:1**	**18:2**	**18:3**	
RG10	124.9 ± 1.6^a^	39.3 ± 1.0^a^	154.5 ± 3.0^a^	658.3 ± 3.3^a^	23.1 ± 0.5^a^	3.4 ± 0.1^a^
PI 361088B	131.7 ± 1.2^b^	37.9 ± 0.8^a^^b^	137.4 ± 1.9^b^	639.1 ± 3.1^b^	53.9 ± 1.3^b^	7.8 ± 0.2^b^
OX948	121.9 ± 1.9^a^^c^	36.0 ± 0.6^b^^c^	219.2 ± 7.2^c^	495.2 ± 5.7^c^	127.7 ± 2.1^c^	20.5 ± 0.2^c^
Commercial cultivars^c^	110	50	210	550	80	<9%^d^ recessive ≥9% dominant

a Fatty acid composition (mean ± standard error): 16:0 palmitic acid, 18:0 stearic acid, 18:1 oleic acid, 18:2 linoleic acid and 18:3 linolenic acid; the values (averages from 10 seed analyses) followed by the same letter are not significantly different at the 0.05 probability level based on the t-tests

b *Desaturation: 18:2 D (ω-3 desaturase) = [(18:3)/(18:2 + 18:3)] × 100 (Cherif et al., 1975)*.

c *(Wilcox, 1989)*.

d *(Wilson et al., 1990)*.

### *FAD3*genes in RG10 and OX948 and comparison with *FAD3* genes in reference williams 82 (Wm82)

Comparisons of the sequences for the four *FAD3* genes from the low linolenic acid (RG10) and wild-type line (OX948) to the GenBank/Phytozome sequences (Figure S1–S4) confirmed that the amplified DNA fragments corresponded to *FAD3A, FAD3B, FAD3C*, and *FAD3D* (*FAD3-2b*, Anai et al., 2005) genes, respectively.

#### *FAD3A* gene

**Figure 2:**
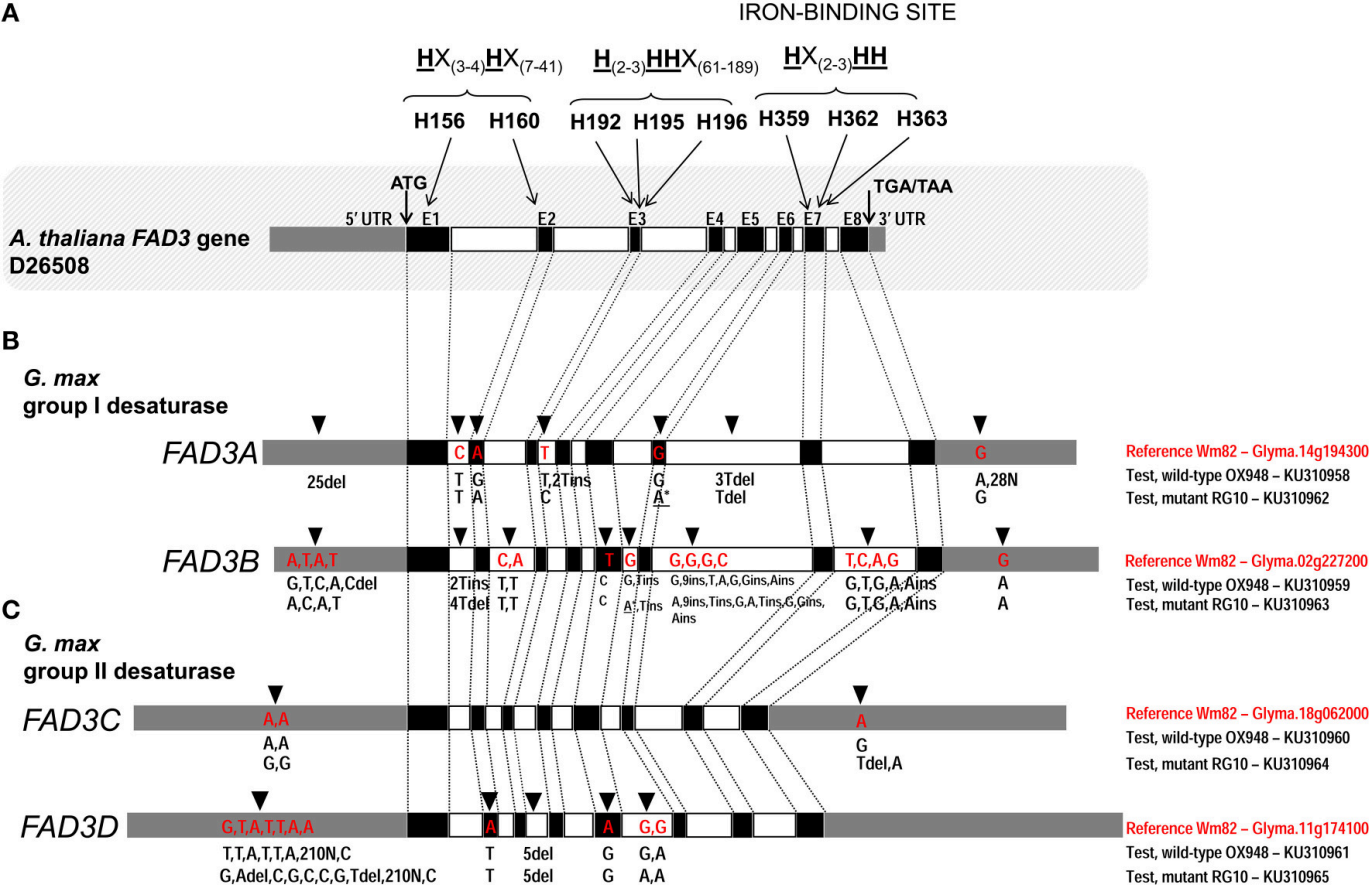
**Comparison of the ω-3 fatty acid desaturase (***FAD3***) genes in test lines, RG10 and OX948, with the Williams 82 (Wm82) reference sequence**. **(A)** *Arabidopsis thaliana FAD3* gene; three conserved histidine boxes (exons 1–2, 3, and 7) and iron-binding sites are indicated. **(B)** *Glycine max FAD3A* and *FAD3B* genes; **(C)** *Glycine max FAD3C* and *FAD3D* genes. Germplasm, gene and GenBank accession are indicated for each sequence on the right of each gene model. In each bar, black boxes represents exons (E1–E8), clear boxes represent introns and gray boxes represent untranslated regions (UTR). The positions of the unique features in each sequence are indicated by an arrow [on the top of the bar (Wm82)] and the feature type (A/T/C/G for a single nucleotide difference and ins/del for inserion and deletions). The positions of mutations in *FAD3A* and *FAD3B* genes in RG10 are indicated by an asterisk (^*^).

The sizes of the *FAD3A* genes sequenced in this study were 5766 bp in RG10 and 5764 bp in OX948 including, approximately, 1000 bp upstream and 860 bp downstream of the open reading frame (ORF) (Table 2; Figure 2). The “TATA” box (TATAAA) is located between nucleotides 915 to 920 in RG10 and 913 through 918 in OX948. The start codon (ATG) occurs at position 1043 in RG10 and 1041 in OX948. The stop codon (TGA) is at 4909 in RG10 and 4907 in OX948. The polyadenylation [poly(A)] signal is located at position 5086 in RG10 and 5084 in OX948 (Figure S1). The OX948 ORF (1131 bp) encodes 376 amino acids with an estimated molecular weight of 43,945 daltons. Because of a mutation that introduces a stop codon in exon 6 (Reinprecht et al., 2009), the RG10 ORF (1131 bp) encodes a smaller protein of 265 amino acids with an estimated molecular weight of 30,783 daltons.

**Table 2:** **Nucleotide sizes (base pairs, bp) of exons and introns and predicted proteins (amino acids, aa; dalton, Da) of four soybean ω-3 fatty acid desaturase (***FAD3***) genes in RG10, OX948, and Williams 82 (Wm82)**.

**Structure**		ω**-3 fatty acid desaturase (*****Fad3*****) gene**^**a**^											
		***FAD3A***			***FAD3B***			***FAD3C***			***FAD3D***		
		**RG10**	**OX948**	**Wm82**	**RG10**	**OX948**	**Wm82**	**RG10**	**OX948**	**Wm82**	**RG10**	**OX948**	**Wm82**
5′ UTR		1042	1040	1090	942	939	1003	2066	2066	2083	2076	2078	2133
Exon 1		293	293	293	305	305	305	296	296	296	299	299	299
Intron 1		167	167	167	188	194	192	160	160	160	254	254	254
Exon 2		90	90	90	90	90	90	90	90	90	90	90	90
Intron 2		324	324	324	348	348	348	123	123	123	121	121	121
Exon 3		67	67	67	67	67	67	67	67	67	67	67	67
Intron 3		135	137	135	142	142	142	166	166	166	164	164	169
Exon 4		93	93	93	93	93	93	93	93	93	93	93	93
Intron 4		109	109	109	98	98	98	156	156	156	219	219	219
Exon 5		186	186	186	186	186	186	186	186	186	186	186	186
Intron 5		292	292	292	116^c^	116	115	148	148	148	387	387	387
Exon 6		81^c^	81	81	81	81	81	81	81	81	81	81	81
Intron 6		1036	1034	1037	1245	1243	1232	361	361	361	345	345	345
Exon 7		138	138	138	138	138	138	138	138	138	138	138	138
Intron 7		673	673	673	627	627	626	277	277	277	323	323	323
Exon 8		183	183	183	183	183	183	192	192	192	192	192	192
3′ UTR		857	857	1069	1135	1135	1183	2226	2227	2270	1685	1083	2275
Total DNA		5766	5764	6027	5984	5985	6082	6826	6827	6887	6720	6120	7372
Total coding region		1131	1131	1131	1143	1143	1143	1143	1143	1143	1146	1146	1146
Total introns		2736	2736	2737	2764	2768	2753	1391	1391	1391	1813	1813	1818
TATA-box		915	913	963	808	805	869	1663	1663	1680	2009	2011	2066
Poly (A)		5086	5084	5135	5009	5010	5059	4961	4961	4978	5481	5483	5543
Predicted protein^b^	aa	265	376	376	185	380	380	380	380	380	381	381	381
	Da	30783	43945	43944	21234	44136	44135	43937	43937	43937	44072	44072	44147

a *FAD3A: KU310962 (RG10), KU310958 (OX948), Glyma.14g194300 [Wm82 (Williams 82)]; FAD3B: KU310963 (RG10), KU310959 (OX948), Glyma.024g227200 [Wm82 (Williams 82)]; FAD3C: KU310964 (RG10), KU310960 (OX948), Glyma.18g062000 [Wm82 (Williams 82)]; FAD3D: KU310965 (RG10), KU310961 (OX948), Glyma.11g174100 [Wm82 (Williams 82)]*.

b *Predicted protein size (amino acid, aa) and molecular weight (dalton, Da; computed at ExPaSy at http://web.expasy.org/compute_pi/)*.

c Mutations (^*^M) in FAD3A and FAD3B genes, respectively (Reinprecht et al., 2009).

The comparison of the *FAD3A* genes in RG10 and OX948 with the corresponding Wm82 (current annotation Wm82.a2.v1) sequence revealed differences in both coding and noncoding regions (Figure 2). Two substitutions were detected in the coding region. One change in exon 2 (A to G) in OX948 did not change the amino acid (leucine, L109) that was encoded (CTA to CTG). The second change, detected in exon 6 in RG10, results in a substitution of an A for a G, and a codon change from TGG for tryptophan (W236) in OX948 and Wm82 to a stop codon (TGA) in RG10. Several differences among the sequences were found in intronic and UTR regions. A single nucleotide change (C to T) was detected in intron 1 of RG10 and OX948 compared to the Wm82 sequence. One T to C change was detected in intron 3 in RG10 and two T insertions were found in the OX948 sequences. One T and 3T deletions were detected in intron 6 in RG10 and OX948, respectively, compared to the reference Wm82 sequence. In addition, OX948 had 25 single nucleotide substitutions in the 5′UTR sequence and two G to A changes and 28 Ns in its 3′UTR region compared to the RG10 and Wm82 sequences (Figure 2 and Figure S1).

#### *FAD3B* gene

The sizes of the *FAD3B* gene sequenced in this study were 5984 bp in RG10 and 5985 bp in OX948 (Table 2; Figure 2). The “TATA” box occurs between nucleotides 808 and 813 in RG10 and between nucleotides 805 to 810 in OX948. The start codon (ATG) occurs at position 943 in RG10 and 940 in OX948. The stop codon (TGA) is located at 4849 in RG10 and 4850 in OX948. The poly (A) signal is in position 5009 in RG10 and 5010 in OX948 (Figure S2). The OX948 ORF (1143 bp) encodes 380 amino acids with an estimated molecular weight of 44,136 daltons. Because of the mutation at a splicing junction of intron 5 (Reinprecht et al., 2009), the RG10 ORF (1143 bp) encodes a smaller protein of 185 amino acids with an estimated molecular weight of 21,234 daltons.

A comparison of the *FAD3B* genes of the mutant (RG10) line and wild-type line (OX948) with the reference Wm82 sequence identified a single point mutation (T to C) in exon 5 of the sequence (Figure 2), which did not change the amino acid (phenylalanine, F204) encoded in this position in RG10 and OX948. Differences between OX948 and RG10 compared to the reference sequence were also detected in intronic regions including: in intron 1 (4T deletion in RG10 and 2T insertion in OX948), intron 2 (C to T and A to T changes in both RG10 and OX948), intron 5 (G to A change in RG10 and T insertion in both RG10 and OX948), intron 6 [G to A and 2T insertions in RG10, G to T change in OX948 and several changes detected in both RG10 and OX948 (G to A substitution, C to G change, two single nucleotide insertions and one nine-nucleotide insertion)], and in intron 7 (four single nucleotide substitutions and one A insertion in both RG10 and OX948 compared to the reference). In the *FAD3B* 5′UTR of the RG10 sequence a single T to C substitution was found. In the 5′UTR of the OX948 sequence, three substitutions (A to G, A to C, and T to A) and a C deletion were found, compared to the RG10 and reference Wm82 sequences. Both RG10 and OX948 had a single G to A substitution at the 3′UTR of the *FAD3B* gene (Figure 2 and Figure S2).

#### *FAD3C* gene

The sizes of the *FAD3C* gene sequenced in the present study were 6826 bp in RG10 and 6827 bp in OX948 (Table 2; Figure 2). The “TATA” box is located between nucleotides 1663 through 1668 in both RG10 and OX948. The start codon (ATG) occurs at position 2067 in both RG10 and OX948. The stop codon (TAA) is at position 4600 in both RG10 and OX948. The poly (A) is located at position 4961 in both RG10 and OX948 (Figure S3). The ORF (1143 bp) encodes a protein of 380 amino acids with an estimated molecular weight of 43,937 daltons in both RG10 and OX948.

There were no differences in the coding or intronic regions of the *FAD3C* genes in RG10 and OX948 when compared to the Wm82 reference sequence. The only changes detected were two A to G substitutions in the 5′UTR and one T deletion in the 3′UTR in the RG10 sequence compared to the OX948 and the Wm82 reference sequences. In the 3′UTR of the OX948 sequence, a single A to G substitution was found, compared to the RG10 and reference Wm82 sequences (Figure 2 and Figure S3).

#### *FAD3D* gene

The sizes of the *FAD3D* gene sequenced in the present study were 6720 bp in RG10 and 6120 bp in OX948 (Table 2; Figure 2). The “TATA” box is located between nucleotides 2009 through 2014 in RG10 and from 2011 to 2016 in OX948. The start codon (ATG) occurs at position 2077 in RG10 and 2079 in OX948. The stop codon (TAA) is at 5035 in RG10 and 5037 in OX948. The poly (A) is located at position 5481 in RG10 and at 5483 in OX948 (Figure S4). The ORF (1146 bp) encodes a protein of 381 amino acids with an estimated molecular weight of 44,072 daltons in both RG10 and OX948.

Two single nucleotide differences were detected in the coding regions of both the RG10 and OX948 *FAD3D* genes compared to the Wm82 reference sequence (Figure 2). An A to T substitution in exon 2 changed methionine M110 to leucine and the substitution of an A for a G in exon 5 changed the codons for aspartate D232 to glycine in OX948 and RG10. Five nucleotide deletions in intron 3 were detected in RG10 and OX948 compared to the Wm82 reference sequence. One G to A substitution was detected in the intron 5 sequence of RG10 relative to Wm82. Additional G to A substitutions (relative to Wm82) were found in both RG10 and OX948. Further differences were found in the 5′UTR regions. Five single base substitutions and two deletions (A and T) were detected in the RG10 5′UTR when compared to the OX948 and the reference Wm82 sequences. A single G to T substitution was found in the OX948 sequence relative to the other two. Additional A to C substitutions were detected in both the RG10 and OX948 sequences compared to the Wm82. Because of the low quality sequencing, a sequence stretch of 210 bp (position 1524–1733 in Wm82) is missing from RG10 and OX948 5′UTR regions of the *FAD3D* gene (Figure 2 and Figure S4).

The four *FAD3* genes in RG10 and OX948 have similar structures, consisting of eight exons and seven introns (Table 2; Figure 2). While the sizes of exons two to seven are conserved among the four *FAD3* genes, the size of exon 1 is different in all four genes (293 bp in *FAD3A*, 305 bp in *FAD3B*, 296 bp in *FAD3C*, and 299 bp in *FAD3D*). Exon 8 is slightly larger in *FAD3C* and *FAD3D* genes (192 bp) compared to the *FAD3A* and *FAD3B* genes (183 bp). In contrast, intron 7 has a different size in the four *FAD3* genes. Among the four genes, *FAD3A* has the shortest intron 3 and the longest intron 7; *FAD3B* gene has the longest introns 2 and 6 and the shortest introns 4 and 5; *FAD3C* has the longest intron 3 and the shortest introns 1 and 7; and *FAD3D* is characterized by the longest introns 1, 4, and 5 and the shortest introns 2 and 6. *FAD3C* gene has the smallest total intron size (1391 bp). The *FAD3A* and *FAD3B* genes have similar total intron sizes (2736 bp and 2764 bp, respectively), which is almost double that found in *FAD3C* (Table 2). All introns start with GT (except intron 5 in RG10 *FAD3B* gene) and end with AG. The *FAD3A* ORF (OX948) encodes the smallest protein (376 amino acids in wild-type OX948) while the ORFs of other three genes encode slightly larger proteins of similar sizes (*FAD3B*, encodes 380 amino acids in wild-type OX948; *FAD3C* and *FAD3D*, encode 381 amino acids in OX948; Figure S5).

### Positions of the *FAD3* genes in sequence and genetic maps and correspondence to linolenic acid QTL

In soybean genome, four *FAD3* genes are positioned on four different chromosomes. Numerous syntenic regions were found for soybean *FAD3* genes with 47 plant species that are currently available in PGDD (Table S2). Soybean Glyma.14g194300 (*FAD3A* gene) on the chromosome Gm14 was syntenic to 52 regions in 27 different plant species (6 to 248 anchor genes). Forty nine syntenic blocks were found for Glyma.02g227200 (*FAD2B* gene) with 25 plant species (6 to 254 anchors) and 53 for Glyma.18g062000 (*FAD3C* gene) with 27 plant species (6 to 302 anchors). The smallest number of syntenic regions was found for Glyma.11g174100 (*FAD3D* gene). The region on the chromosome Gm11 containing Glyma.11g174100 was syntenic to 36 regions in 21 plant species (7 to 76 anchor genes).

**Figure 3:**
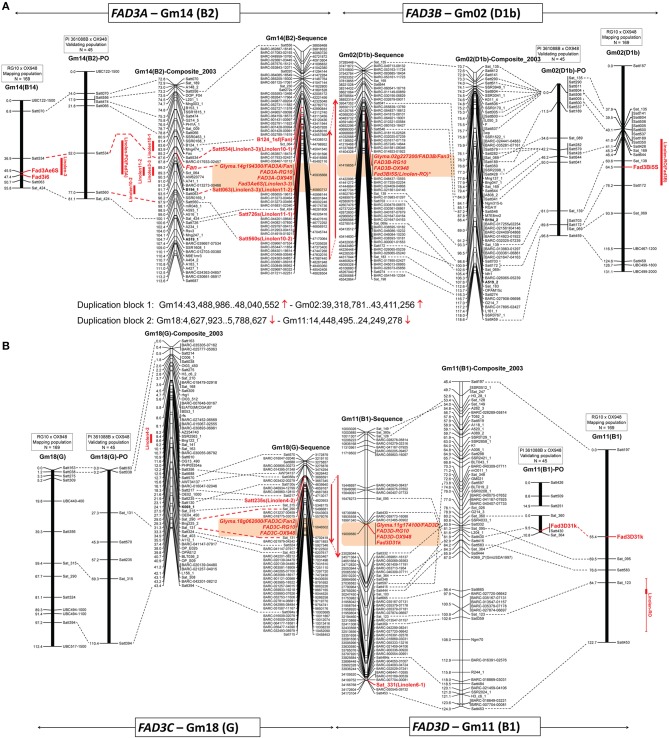
**Positions of the ω-3 fatty acid desaturase (***FAD3***) genes on three linkage maps (mapping population RG10 × OX948, confirmation population PI 361088B × OX948 and GmComposite_2003) and soybean sequence map**. **(A)** *FAD3A* and *FAD3B* genes on chromosomes Gm14 (B2) and Gm02 (D1b); **(B)** *FAD3C* and *FAD3D* genes on chromosomes Gm18 (G2) and Gm11 (B1). *FAD3* genes are shown in red, bold; previously mapped features (Reinprecht et al., 2006) are underlined. The name of each map is indicated on the top of the bar or line. Coordinates of genome features on sequence (bp, sequence start) and genetic GmComposite_2003 (cM) maps (SoyBase) were used to construct the alignments for chromosome pairs (Gm14 and Gm02) containing FAD3A/FAD3B and FAD3C/FAD3D groups of desaturases, respectively. The sequence maps are placed in the middle of each alignment and are connected through duplication blocks.

**Figure 4:**
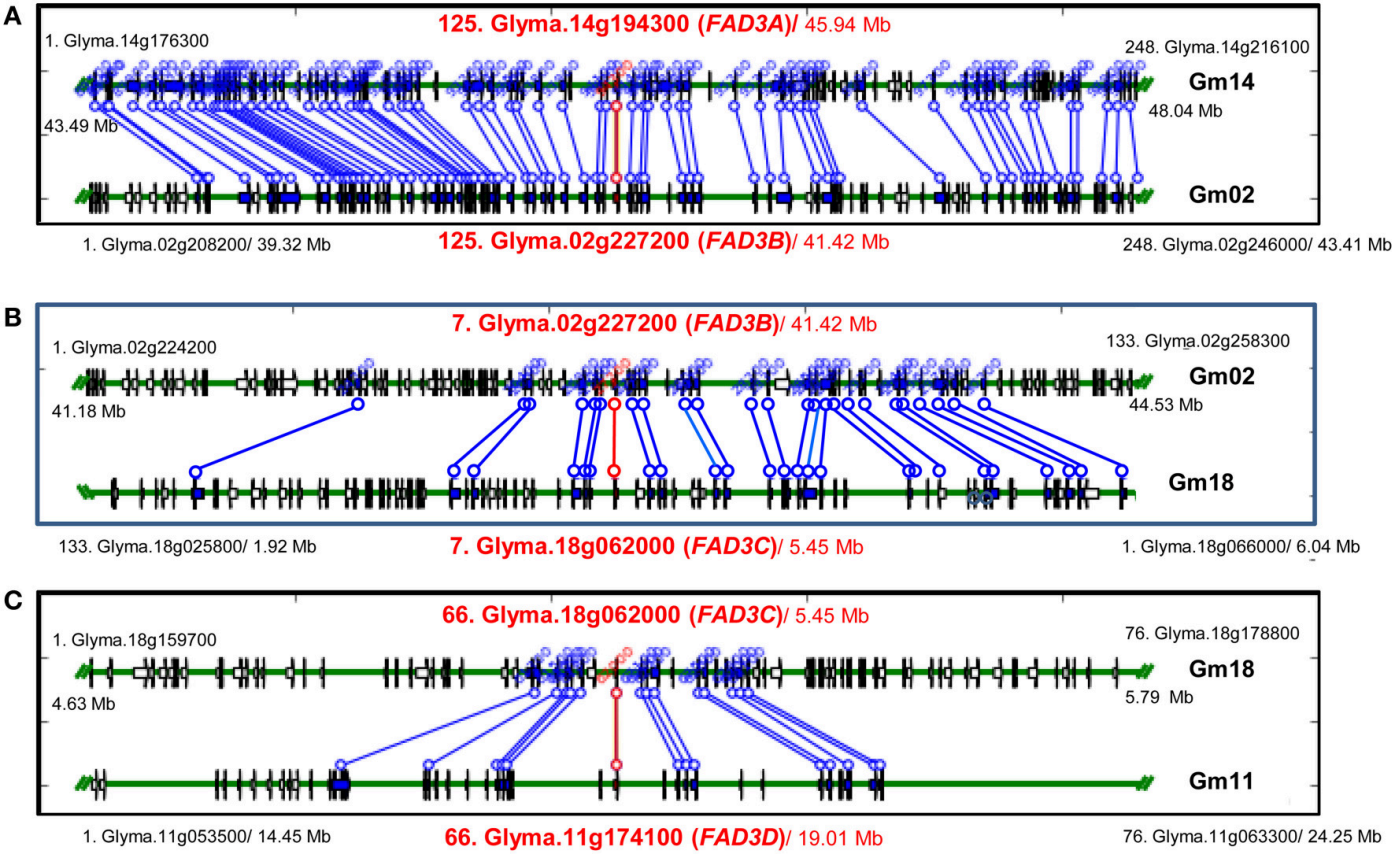
**Syntenic regions containing ***FAD3*** genes in soybean genome. (A)** Regions containing *FAD3A* and *FAD3B* genes; **(B)** Regions containing *FAD3B* and *FAD3C* (in reverse orientation) genes; **(C)** Regions containing *FAD3C* and *FAD3D* genes. Syntenic regions were identified using Plant Genome Duplication Database (PGDD). All blocks for the query locus displayed ± 500 Kb region. Query locus, as well as first and last loci in each block are indicated. Each locus identifier consists of a number, indicating locus order in the syntenic block, locus name and a start position in the block (Mb).

Regions containing *FAD3* genes in soybean genome are syntenic (Table 3). Group I ω-3 FAD genes *FAD3A* and *FAD3B* are found on homeologous regions of chromosomes Gm14 (B2) and Gm02 (D1b), respectively. Large duplication blocks consist of 4,551,566 bp in Gm14 (position Gm14:43,488,986.48,040,552) and 4,093,688 bp in Gm02 (position Gm02:39,317,568.43,411,256) with 248 anchor genes (Figures 3A, 4 and Table S2). The *FAD3A* gene is at the locus Gm14.g194300 on the chromosome Gm14 (position Gm14:45,935,668.45,939,896) and the *FAD3B* gene is at the locus Gm.02g227200 on the chromosome Gm02 (position Gm02:41,419,656.41,423,881; Wm82.a2.v1). Group II desaturase genes, *FAD3C* and *FAD3D*, are found on homeologous regions on the chromosomes Gm18 (G) and Gm11 (D1b), respectively. Duplication blocks consist of 1,160,704 bp (position Gm18:4,627,923.5,788,627) in Gm18 and 9,800,783 bp in Gm11 (position Gm11:14,448,495.24,249,278) with 76 anchor genes (Figures 3B, 4 and Table S2). The *FAD3C* gene is at the locus Gm.18g062000 on the chromosome Gm18 (position Gm18:5,646,502.5,649,337) and the *FAD3D* gene is at the locus Gm.11g174100 on the chromosome Gm11 (position Gm11:19,009,581.19,012,951).

**Table 3:** **Syntenic blocks containing ***FAD3*** loci in soybean (Wm82.a2.v1) genome**.

***FAD3*** **locus**		**Syntenic block**^**a**^			**Position within block**	**Ka^b^**	**Ks^c^**	**Ka/Ks**
**Query**	**Synteny**	**Score**	***E*-value**	**Anchors (# genes)**				
Glyma.14g194300	Glyma.02g227400	9852.0	2e-158	248	125	0.02	0.13	0.154
	Glyma.11g174100	1483.0	8e-179	39	28	0.15	0.96	0.156
	Glyma.18g062000	3013.0	1e-101	78	7	0.14	0.84	0.167
Glyma.02g227200	Glyma.11g174100	1688.0	0.0	44	11	0.15	0.90	0.167
	Glyma.18g062000	5176.0	1e-98	133	7	0.15	0.78	0.192
Glyma.18g062000	Glyma.11g174100	2991.0	1e-43	76	66	0.02	0.12	0.167

a *Related syntenic regions in soybean genome by locus identifier were obtained from the Plant Genome Duplication Database (PGDD, available at: http://chibba.agtec.uga.edu/duplication/index/locus; accessed 2 Oct 2015)*.

b *Nonsynonymous substitution rates*.

c *Synonymous substitution rates*.

### *FAD3A* gene, *Fan* allele, and linolenic acid QTL

BLAST searches against the soybean genome (current annotation Wm82.a2.v1; http://phytozome.jgi.doe.gov/pz/portal.html) placed RG10 and OX948 sequences for the *FAD3A* gene on the chromosome Gm14 at the locus Glyma.14g194300 (*FAD3A*) between SSR markers Satt534 and Satt726 in soybean sequence map (position Gm14:45,935,668.45,939,896). The *Fan* locus and five linolenic acid QTL [Linolen 3-3 (Reinprecht et al., 2006), Linolen 10-1 and Linolen 10-2 (Spencer et al., 2004), Linolen 11-1 and Linolen 11-2 (Xie et al., 2012); SoyBase] were mapped to the same region in the genetic GmComposite_2003 map (Figure 3A).

#### RG10 × OX948

In the current work, to fine map the *FAD3A* gene, six new SSR markers were selected from the *FAD3A* gene region. However, only one marker (Sat_424) was polymorphic between the parents (RG10 and OX948) and its addition to the map did not change position of the *FAD3A* gene (Figure 3A). The current size of the chromosome Gm14-based linkage group (B2) in the RG10 × OX948 population is 81.1 cM.

#### Validation of a major linolenic acid QTL in the PI 361088B × OX948 population

To validate the linolenic acid QTL detected with the RG10 × OX948 population, markers on chromosomes Gm02, Gm11, Gm14, and Gm18 were scored for individuals from the PI 361088B × OX948 population. A relatively small set of SSR markers on four chromosomes was shared between the two populations. In the PI 361088B × OX948 population, chromosome Gm14 (B2) contains eight markers, four of which are shared with the mapping RG10 × OX948 population. A major QTL (LOD 8), which explains 59% variability for the linolenic acid content in the PI 361088B × OX948 population was detected between the markers Satt534 and Satt560. This is the same region as the location of the major linolenic acid QTL Linolen 3-3 (Reinprecht et al., 2006; SoyBase) in the RG10 × OX948 mapping population (Figure 3A) and associated RG10 and OX948 *FAD3A* gene sequences (locus Glyma.14g194300).

### *FAD3B* gene, *Fan3* allele and linolenic acid QTL

The RG10 and OX948 sequences for the *FAD3B* gene were placed on the chromosome Gm02 at the position Gm02:41,419,656.41,423,881 at the locus Glyma.02g227200 (*FAD3B)* in the soybean sequence map (Wm82.a2.v1). There was no linolenic acid QTL on chromosome Gm02 (D1b) of the GmComposite_2003 genetic map corresponding to the *FAD3B* gene location in the sequence map (Figure 3A).

#### RG10 × OX948

A SNP marker FAD3B-i5SNP, developed previously for the mutation in the *FAD3B* gene (Reinprecht et al., 2009), mapped initially to the end of the top portion of the chromosome Gm02 (D1b, 21.4 cM from the SSR marker Sat_135) in the RG10 × OX948 map (not shown). To fine map this region, 21 SSR markers were selected in the region of the *FAD3B* gene and 12 were placed on the RG10 × OX948 map. The addition of new markers allowed fragments of chromosome Gm02 (D1b) to be joined. The current size of this chromosome-based linkage group in the RG10 × OX948 map is 131.1 cM and it contains 21 markers. The *FAD3B* gene/marker FAD3B-i5SNP is positioned between the SSR markers Sat_139 (3.9 cM) and Satt172 (11.7 cM) in the region of the newly identified linolenic acid QTL [Linolen-RO(FAD3B), Figure 4A] and corresponds well with the *in silico* position of the RG10 and OX948 *FAD3B* gene sequences at the locus Glyma.02g227200 (*FAD3B*). This QTL (LOD 6.12) explained approximately 10% variability for linolenic acid content and was consistently expressed in all three Ontario locations (Harrow 8%, Woodslee 9%, and Ridgetown 11%) with an additive effect ranging from −9.74 g kg^−1^ (Harrow) to −11.14 g kg^−1^ (Woodslee).

#### PI 361088B × OX948

Chromosome Gm02 (D1b) in the validating population contains 20 markers, 10 of which are in common with the RG10 × OX948 mapping population. No QTL was identified in the region that corresponds to the region of the minor linolenic acid QTL (markers Sat_139 and Satt172) and the associated *FAD3B* gene (locus Gm.02g227200) in the RG10 × OX948 mapping population (Figure 3A).

### *FAD3C* gene, *Fan2* allele, and linolenic acid QTL

The RG10 and OX948 *FAD3C* gene sequences were mapped to the chromosome Gm18 (G) at the locus Glyma.18g062000 (*FAD3C*, position Gm18:5,646,502.5,649,337) between SSR markers Satt217 and Satt324 on the soybean sequence map (Wm82.a2.v1). QTL Linolen 4-2 (SoyBase; Panthee et al., 2006) was mapped to the same location on the chromosome Gm18 (G) in the GmComposite_2003 genetic map.

#### RG10 × OX948

Nine SSR markers were selected from the *FAD3C* gene region and two markers (Sat_315 and Sat_290) were placed on the RG10 × OX948 map in the region predicted to contain the *FAD3C* gene, between previously mapped markers Satt356 and Satt324 (Figure 4B). A SNP marker for the *FAD3C* gene was developed based on differences in the 5′UTR region between RG10 and OX948 (Figure S3). Optimization of the marker and subsequent mapping are underway. The size of the chromosome Gm18-based linkage group (G) in the RG10 × OX948 population is 112.4 cM. No linolenic acid QTL was detected in the *FAD3C* gene region of the RG10 × OX948 map (Figure 3B).

#### PI 361088B × OX948

Chromosome Gm18 (G) in the PI 361088B × OX948 derived map contains only seven markers. Four SSR markers were common between the mapping RG10 × OX948 population and the validating PI 361088B × OX948 population. No markers were mapped and no linolenic acid QTL were detected in the region corresponding to the *FAD3C* gene (locus Glyma.18g062000) in sequence map (Figure 3B).

### *FAD3D* gene and linolenic acid QTL

The RG10 and OX948 *FAD3D* gene sequences were mapped on the chromosome Gm11 (B1) at the locus Glyma.11g174100 (*FAD3D*, position Gm11:19,009,581.19,012,951) on the soybean sequence map (Wm82.a2.v1). No associations to the *Fan* alleles were reported and there is no linolenic acid QTL on the chromosome Gm11 (B1) of the GmComposite_2003 genetic map (SoyBase), which corresponds to the *FAD3D* gene location in soybean sequence map (Figure 3B).

#### RG10 × OX948

Fourteen SSR markers were selected from the *FAD3D* gene region and screened for polymorphism with the parental genomic DNA. Three SSR markers were mapped on the chromosome Gm11 (B1) in the RG10 × OX948 population. A SNP marker (Fad3D31k) for the *FAD3D* gene was developed, based on the difference in the 3′UTR region in RG10 and OX948 sequences (Figure S4). It mapped to the chromosome Gm11 (B1), 14.1 cM from the SSR marker Sat_095 (Figure 4B). The size of the chromosome Gm11-based linkage group (B1) in the RG10 × OX948 population is 67.3 cM. No linolenic acid QTL was detected in the *FAD3D* gene region of the RG10 × OX948 map. However, a minor QTL associated with the linolenic acid was detected downstream of the *FAD3D* gene-based marker location, which was associated with the marker Sat_123 (Figure 3B).

#### PI 361088B × OX948

In the validating population, chromosome Gm11 (B1) is broken into two segments with three and four markers, respectively. The *FAD3D* gene-based marker (Fad3D31k) mapped to the bottom portion of the chromosome linked to the SSR marker Satt430 and 8.7 cM apart from Satt298. This position corresponds well to the region of the RG10 and OX948 *FAD3D* gene sequences (locus Glyma.11g174100) in the RG10 × OX948 mapping population. Moreover, this is the only marker connection between the two populations. However, no QTL was detected in the area of the *FAD3D* gene in the PI 361088B × OX948 population.

## Discussion

### Linolenic acid content and ω-3 fatty acid desaturase activity in parental lines

The low linolenic acid parental genotypes used in the present study, RG10 and PI 361088B, had significantly different levels of linolenic acid and each had significantly lower level of linolenic acid compared to the wild-type parent, OX948. The linolenic acid level for PI 36188B was higher in this study (54 g kg^−1^) than previously reported (39 g kg^−1^) by Rennie and Tanner (1989). A similar difference between studies was reported for the line C1640 (Brummer et al., 1995), and was probably due to testing in different environments.

It was shown previously that reduced levels of linolenic acid in lines RG10 and PI 361088B are controlled by homozygous recessive alleles with additive effects at the *Fan* locus (Rennie et al., 1988; Stojšin et al., 1998; Reinprecht et al., 2005). This locus encodes ω-3 FAD (Brummer et al., 1995), which catalyzes the desaturation of linoleic to linolenic acid. Wilson et al. (1990) suggested that in *Glycine max*, values of 18:2 desaturation lower than 9% are associated with recessive alleles for ω-3 FAD. Conversely, values equal or higher than this threshold value are associated with dominant allele. According to this criteria, the low linolenic acid lines RG10 and PI 361088B are homozygous recessive for ω-3 FAD. However, the activity of ω-3 FAD in line PI 361088B was more than double that observed in line RG10 (18:2D = 3.4 and 7.8%, respectively). Consistent with this, the level of linolenic acid in RG10 seeds was about half of that in seeds of PI 361088B. In contrast, the high level of linolenic acid in the wild-type parent (OX948) indicates that it contained a pair of dominant alleles for ω-3 FAD. Furthermore, the estimate of 20.5% for the relative 18:2 desaturation value in OX948 was significantly higher than typically found for dominant ω-3 FAD alleles in *G. max*. This estimate is similar to the relatively high levels of 18:2 desaturation found in *Glycine soja* plant introductions. Values of 16.8% in PI 342434 for 18:2 desaturation and 21.1% in PI 424031 have previously been reported by Pantalone et al. (1997). These authors suggested that “different complement or additional copies of genes” determine levels of linolenic acid in *G. soja* and this may apply to the high levels of linolenic acid detected in the line OX948 in the present study.

### Sequences of *FAD3* genes in RG10 and OX948

The whole length genomic DNA sequences we obtained for *FAD3A, FAD3B, FAD3C*, and *FAD3D* genes in RG10 and OX948 confirmed that the microsomal ω-3 FADs in soybean are 78 to 95% similar (ORF). All four *FAD3* genes have a similar structure and contain eight exons, as reported previously (Iba et al., 1993). In general, the sizes of the exons were well conserved among the *FAD3* genes and corresponded well to the sizes of *FAD3* exons in Arabidopsis. The most variable was exon 1, which has different sizes in all four *FAD3* genes. Exon 8 was slightly shorter in *FAD3A* and *FAD3B* (183 bp) compared to the *FAD3C* and *FAD3D* (192 bp). However, compared to exons, intron sizes were more variable. All seven introns have different sizes in the four *FAD3* genes. Based on the sequence similarity and exon/intron structure, the four *FAD3* genes can be separated into two groups consisting of *FAD3A*/*FAD3B* and *FAD3C*/*FAD3D*, respectively. Within each group, the members share 95% sequence similarity (coding region) and they have similarly sized introns. The longer introns in the *FAD3A*/*FAD3B* gene group are 2, 6, and 7, while the longest introns in the *FAD3C*/*FAD3D* group are 3 and 4. There is 78 to 80% sequence similarity between the groups. The *FAD3A* ORF encodes the smallest protein while the ORFs of other three *FAD3* genes encode slightly larger proteins of similar sizes (except for the *FAD3A* and *FAD3B* in mutant RG10; Figure S5).

### *In silico* map of *FAD3* genes in RG10 and OX948

Previously, we mapped *FAD3A* gene to the linkage group B2 (Gm14) in the RG10 × OX948 population (Reinprecht et al., 2006). The position of the *FAD3A* gene was confirmed by *in silico* mapping of the RG10 and OX948 sequences. BLAST searches against soybean genome (Wm82.a2.v1) placed RG10 and OX948 sequences for the *FAD3A* gene on the chromosome Gm14 at the locus Glyma.14g194300 (*FAD3A*, position Gm14:45,935,668.45,939,896), the *FAD3B* gene on the chromosome Gm02 at the locus Glyma.02g227200 (*FAD3B*, position Gm02:41,419,656;;41,423,881), the *FAD3C* gene on the chromosome Gm18 at the locus Glyma.18g062000 (*FAD3C*, position Gm18:5,646,502.5,649,337) and the *FAD3D* gene on the chromosome Gm11 at the locus Glyma.11g174100 (*FAD3D*, position Gm:19,009,581.19,012,951) (Figure 3). The positions of the *FAD3* genes in the soybean sequence map (Wm82.a2.v1) are slightly different from those reported in an earlier version of the soybean genome sequence (Chi et al., 2011) and might change again with the additional genome annotations.

The availability of the complete genome sequences for numerous plant species allows the organization of the individual genomes to be studied, as well as enables comparison of the genomes at the nucleotide level. Synteny analysis performed with soybean *FAD3* genes against complete genome sequences available for 47 flowering plant species in PGDD identified numerous syntenic regions. Syntenic blocks were of various sizes and contained 6 to 302 gene anchors. The multiple copies of genes in soybean arise from its tetraploid nature (Shoemaker et al., 1996; Schlueter et al., 2007; Roulin et al., 2013). Thus, four copies of ω-3 FADs are theoretically expected and in fact, two pairs of homologous ω-3 FAD regions were detected in the soybean genome sequence (Wm82.a2.v) and, by *in silico* mapping. These were located on chromosomes Gm14 (B2), Gm02 (D1b), Gm18 (G), and Gm11 (B1) (Chi et al., 2011; SoyBase). The regions on chromosomes Gm14 (B2, *FAD3A*) and Gm02 (D1b, *FAD3B*) are syntenic, as well as regions on chromosomes Gm18 (G, *FAD3C*) and Gm11 (B1, *FAD3D*).

### Position of the *FAD3* genes in the linolenic acid QTL regions of the RG10 × OX948 linkage map

Five desaturase gene-specific markers (FAD3i1, FAD3i3, FAD3T, FAD3M, and FAD3B) designed earlier based on the L22964 sequence (Yadav et al., 1993), were removed from the RG10 × OX948 map (Reinprecht et al., 2006). The addition of new SSR and *FAD3* gene-specific markers developed in this study to the chromosome Gm14 (B2), Gm02 (D1b), and Gm11 (B1) changed some distances compared to the original RG10 × OX948 map. It also helped to join two segments of chromosome Gm11 (B1), which initially contained only two SSR markers (Sat_123 and Satt453), as well as two segments of chromosome Gm02 (D1b) in the RG10 × OX948 map.

#### FAD3A and FAD3B genes

The addition of a single SSR marker to the chromosome Gm14 (B2) did not change the position of the previously mapped *FAD3A* gene (Reinprecht et al., 2009). This gene was mapped as a *FAD3A* gene-based marker to the region of a major linolenic acid QTL (Linolen 3-3) on the chromosome Gm14 (B2) in the RG10 × OX948 map (Reinprecht et al., 2006; SoyBase), which is the same genomic region as the previously mapped *fan* allele that conditions low linolenic acid content in C1640 (Brummer et al., 1995). This is also consistent with the previous placement of the microsomal ω-3 *FAD* gene on the chromosome Gm14 (B2) (Byrum et al., 1995) and corresponds well with the *in silico* position of the RG10 and OX948 *FAD3A* gene sequences (this study).

The *FAD3B* gene-based marker developed previously (Reinprecht et al., 2009) was mapped on the chromosome Gm02 (D1b) of the RG10 × OX948 map, between SSR markers Sat_139 and Satt172, in the region of the newly detected linolenic acid QTL [Linolen-RO (FAD3B)]. This corresponds well with the *in silico* position of the RG10 and OX948 *FAD3B* gene sequences.

#### FAD3C and FAD3D genes

Because of the problem with amplification, optimization of a SNP marker developed for the *FAD3C* gene (difference in the 5′UTR region in RG10 and OX948 sequences) and subsequent mapping are underway. No linolenic acid QTL was detected in the *FAD3C* gene region with (based on *in silico* map) the RG10 × OX948 derived RILs. The *FAD3D* gene-based marker mapped to the chromosome Gm11 (B1), between SSR markers Sat_095 and Satt197, which corresponds well to the RG10 and OX948 *FAD3D* gene sequence position in the soybean *in silico* map. No linolenic acid QTL was detected in the *FAD3D* gene region of the RG10 × OX948 derived map. However, a minor QTL that was associated with the marker Sat_123, was detected downstream of this region.

### Validation of a major linolenic acid QTL (Linolen 3-3) in the PI 361088B × OX948 population

In soybean, significant work was done on mapping genomic regions associated with the seed linolenic acid content. Currently, 44 linolenic acid QTL (identified in 10 independent studies) have been deposited in SoyBase (23 Sep 2015). However, information for several QTL from these studies are unavailable since the markers associated with them were not present on the consensus and/or composite genetic maps. Xie et al. (2012) developed several *FAD3* gene-based markers and mapped six linolenic acid QTL in a F_5:7_ population derived from the cross He-Feng 25 × Dongnong L-5 over several environments. However, QTL Linolen 11-3 (marker FAD3b-1) and Linolen 11-4 (marker FAD3c-2) were not reported in SoyBase since these markers are not present on the GmComposite_2003 map. In addition, QTL Linolen 11-2 was associated with SSR marker Satt063 (SoyBase) but not FAD3a-4 marker that was reported originally by Xie et al. (2012). In the current study, sequences of these markers were BLASTed against soybean genome, but because of absence of any significant hits they were not placed on the sequence map. There are two additional linolenic acid QTL studies not cataloged in SoyBase. Brummer et al. (1995) mapped the *Fan* locus between RFLP markers B124_1 and B194_1 on linkage group B2 (Gm14) in an F_2_ population developed from the *G. max* (C1694) × *G. soja* (PI 479750) cross. The QTL at this position explained most of the variation (85%) for the linolenic acid content in that population. Recently, Akond et al. (2014) identified six QTL associated with the levels of linolenic acid in an F_5:7_ population developed from the MD96-5722 × Spencer cross. These QTL were mapped on four chromosomes (Gm13, Gm14, Gm15, and Gm16) and explained 5 to 23% variability for the linolenic acid content in this population. However, linolenic acid QTL reported in both studies as well as three linolenic acid QTL (Linolen 8-1, Linolen 8-2, and Linolen 8-3; SoyBase) reported by Kim et al. (2010) should be considered as putative QTL since their identification was based on a single environment data.

Linolenic acid QTL deposited in SoyBase were identified using diverse populations (type and size), which were evaluated in different environments and analyzed with various methods. Using a meta analysis of publicly available QTL information, Hu et al. (2011) integrated major QTL for numerous important agronomic traits including fatty acids. They identified three regions containing linolenic acid QTL: three overlapping linolenic acid QTL on chromosome Gm14 (B2) between markers Sat_230 and Satt560, a single QTL on chromosome Gm18 (G) between markers Satt356 and Sat_290 and two regions each with two overlapping QTL on chromosome Gm15 (E). However, to be useful for plant breeding published QTL need to be confirmed in an independent population. QTL confirmation will provide new insight into the strengths as well as limitations of QTL implementation in MAS (Young, 1999).

Although numerous QTL for different traits were identified in soybean, very few of them were verified in independent populations. For example, QTL for soybean cyst nematode resistance, initially identified in an F_2:3_ population, were confirmed in F_6:7_ RILs developed from the same Magellan (susceptible) × PI 438489B (resistant) cross (Vuong et al., 2011), while QTL for seed weight, protein and oil contents were confirmed in different genetic backgrounds by two research groups (Fasoula et al., 2004; Pathan et al., 2013). Seven QTL for linolenic acid were mapped on the chromosome Gm14 (B2) in five different populations by four research groups (SoyBase). Four of these QTL (Linolen 10-1, Linolen 10-2, Linolem 11-2) were identified in the region (86.3–98.9 cM, GmComposite_2003) of the *Fan* locus. The major linolenic acid QTL (Linolen 3-3) was detected in all three testing locations (Harrow, Ridgetown, and Woodslee, ON) and explained very similar amounts of the total phenotypic variability (*R*^2^ = 0.76) for linolenic acid content in the RG10 × OX948 mapping population (Reinprecht et al., 2006). This is an example of the most important type of QTL for breeders because it was associated with the *FAD3A* gene (Reinprecht et al., 2009) and confirmed in the PI 361088B × OX948 population (*R*^2^ = 0.59), which may lead to the stability in breeding for low linolenic acid trait. However, because of the small size of the PI 361088B × OX948 confirmation population (*N* = 45) additional work is required to verify that. In this study, we identified a new QTL associated with linolenic acid content in the RG10 × OX948 population. It mapped to the chromosome Gm02 (D1b), which explained ~10% variation for the trait and was associated with the *FAD3B* gene. The usefulness of the previously developed *FAD3A* and *FAD3B* gene mutation-based markers was confirmed with a number of RG10-derived low linolenic acid lines and cultivars with normal level of linolenic acid (Reinprecht et al., 2009). In the current work, we sequenced all four microsomal *FAD3* genes and identified numerous variations between parental lines of mapping population (RG10 and OX948) as well as reference Wm82. In the future, these polymorphisms could be used to develop additional *FAD3* gene-based markers. To be useful for the soybean breeders globally, these markers need to be validated in diverse germplasm.

## Author contributions

All authors listed, have made substantial, direct, and intellectual contribution to the work, and approved it for publication.

### Conflict of interest statement

The authors declare that the research was conducted in the absence of any commercial or financial relationships that could be construed as a potential conflict of interest. The reviewer VS and Handling Editor declared their shared affiliation, and the Handling Editor states that the process nevertheless met the standards of a fair and objective review.
