# Supplementary material for: Microsomal Omega-3 Fatty Acid Desaturase Genes in Low Linolenic Acid Soybean Line RG10 and Validation of Major Linolenic Acid QTL
Source: Front Genet. 2016 Mar 29;7:38. doi: 10.3389/fgene.2016.00038 (PMC4810023; doi:10.3389/fgene.2016.00038)
Supplement: Supplementary file 1 [file DataSheet1.PDF]

Microsomal omega-3 fatty acid desaturase genes in low linolenic acid soybean line RG10 and validation of major linolenic acid QTL

Yarmilla Reinprecht* and K. Peter Pauls

*Correspondence: Yarmilla Reinprecht: yreinpre@uoguelph.ca

FIGURE S1 | Comparison of *FAD3A* gene sequences in Williams 82 (Glyma.14g194300, Wm82.a2.v1), RG10 (KU310962) and OX948 (KU310958); exon sequences are shown in capital letters and intron sequences are shown in small letters; sequence differences are highlighted; mutation (*M) in *Fad3A* gene in line RG10 is shown in red.

```
                                                                                  5'UTR
Glyma.14g194300    TGCTAGGAAGCAATTTGCATGCTAGGAAGCAAAGTAAAATTCTCAAACTGTATAACTTAT 60
OX948              ------------------------------------------------TGTATAACTTAT 12
RG10               ------------------------------------------------TGTATAACTTAT 12
                                                                   ************

Glyma.14g194300    TTTCTCTTGTTGTATATAAAACTAGTCATTTTTCATTAAAAAGCATTGTATAATAGTTTA 120
OX948              TTTCTCTTGTTGTATGTAAAACTAGTCATTTTTCATTAAAAAGCATTGTATAATAGTTTA 72
RG10               TTTCTCTTGTTGTATATAAAACTAGTCATTTTTCATTAAAAAGCATTGTATAATAGTTTA 72
                   *************** ********************************************

Glyma.14g194300    ATGGGTCATTGAAATTATTATAATTAATGTCATTCTATTTTTAATACTCCTTTTGTTTGA 180
OX948              ATAGGTCATTGAAATTATTATAATTAATGTCATTCTATTTTTAATACTCCTTTTGTTTGA 132
RG10               ATGGGTCATTGAAATTATTATAATTAATGTCATTCTATTTTTAATACTCCTTTTGTTTGA 132
                   ** *********************************************************

Glyma.14g194300    TAATGATTATCGTTTCATGTTATTTTCTATACATATCAAGACAAATTAATAAATGGATAA 240
OX948              TAATGATTATCGTTTCATGTTATTATGTATACATATCAAGACAAATTAATAAATGGATAA 192
RG10               TAATGATTATCGTTTCATGTTATTTTCTATACATATCAAGACAAATTAATAAATGGATAA 192
                   ************************ * *********************************

Glyma.14g194300    AAAAGTATCAATTTTATAAAATTAATATTATTATTATTAATTTATTTATAATTTTTTGTT 300
OX948              AAA-GTATCAATTTTATAAAATTAACATTAATATTATTAATTTATTTATAATTTTTTGTT 251
RG10               AAAAGTATCAATTTTATAAAATTAATATTATTATTATTAATTTATTTATAATTTTTTGTT 252
                   *** ********************* **** *****************************

Glyma.14g194300    ATCATTTATATTATAAGTAATATATTATTGGCAAAAATAATTTTTGATCAATTATATTTA 360
OX948              ATCATTTATATTATAAGTAATATATTATTGACAGAAATAATTTTTGATCAATTATACTTA 311
RG10               ATCATTTATATTATAAGTAATATATTATTGGCAAAAATAATTTTTGATCAATTATATTTA 312
                   ****************************** ** ********************** ***

Glyma.14g194300    CCTGTCGGTCGAACTCTAGATTATGCTGGGTATCTTCTCCAAATGAATCCAAAGATTAAA 420
OX948              CCTCTCGATCGAACTCTAGATTAT-TTAGATACCTTCTCCAGATGAACCCAAAGATTAAA 370
RG10               CCTGTCGGTCGAACTCTAGATTATGCTGGGTATCTTCTCCAAATGAATCCAAAGATTAAA 372
                   *** *** ****************  * * ** ******** ***** ************

Glyma.14g194300    ATAAAATAAAATTATAAGTAATATAAATAAAAAACAATTAATACTAGATTAACAAGACTA 480
OX948              ATAAAATAAAATTATAAGTAATATAAATAAAAAACAATTAATTCTACGTTAACAAGACTA 430
RG10               ATAAAATAAAATTATAAGTAATATAAATAAAAAACAATTAATACTAGATTAACAAGACTA 432
                   ****************************************** ***  ***********

Glyma.14g194300    AAATAATAATTATTTTATAATTTATTTTCTTCAATAATTGTAGAATACAAGGAGTAATAT 540
OX948              AAATAATAATTATTTTATAATTTATTTTCTTCAATAATTGTAGAATACAAGGAGTAATAT 490
RG10               AAATAATAATTATTTTATAATTTATTTTCTTCAATAATTGTAGAATACAAGGAGTAATAT 492
                   ************************************************************
```

```
Glyma.14g194300    TTAATGTTGTTTAATTCTTGTTTCAATAATTGAGATGTTTTGAACAAATTAAATAATTAT 600
OX948              TTAATGTTTTTTAATTCTTGTTTCAATAATTGAGATGTTTTGAACAAATTAAATAATTAT 550
RG10               TTAATGTTGTTTAATTCTTGTTTCAATAATTGAGATGTTTTGAACAAATTAAATAATTAT 552
                   ******** ***************************************************

Glyma.14g194300    TGTAAATAGAATAACATTAATTACAATAATAAAATCATTTTAACGATCCATTAAACTTAA 660
OX948              TGTAAATAGAATAACATTAATTACAATAATAAAATCATTTTAACGATCCATTAAACTTAA 610
RG10               TGTAAATAGAATAACATTAATTACAATAATAAAATCATTTTAACGATCCATTAAACTTAA 612
                   ************************************************************

Glyma.14g194300    ATGATAAAATTCAACTAACTAATTTGGAGTAATTAAGAAAAATAGTTAATTTAGACAACA 720
OX948              ATGATAAAATTCAACTAACTAATTTGGAGTAATTAAGAAAAATAGTTAATTTAGACAACA 670
RG10               ATGATAAAATTCAACTAACTAATTTGGAGTAATTAAGAAAAATAGTTAATTTAGACAACA 672
                   ************************************************************
Glyma.14g194300    ATATTAAATTTTTGCTAAATTATATGTTTTTCTCAAAATTACCTATAACATTAATAAGAC 780
OX948              ATATTAAAGTTTTGCTAAATTATATGTTTTTCTCAAAATTACCTATAACATTAATAAGAC 730
RG10               ATATTAAATTTTTGCTAAATTATATGTTTTTCTCAAAATTACCTATAACATTAATAAGAC 732
                   ******** ***************************************************

Glyma.14g194300    ATACTTTTATTTTTCAAAGATTTCTACTTAATTAACCGCCACAAATTCATCCTCGCTGGT 840
OX948              ATACTTTTATTTTTCAAAGATTTCTACTTAATTAACCGCCACAAATTCATCCTCGCTGGT 790
RG10               ATACTTTTATTTTTCAAAGATTTCTACTTAATTAACCGCCACAAATTCATCCTCGCTGGT 792
                   ************************************************************

Glyma.14g194300    TTGTCCTACACCGTATGTTTTTTGACGTCAGCTAGGCAAACCAACATAAATAGGAAGCAG 900
OX948              TTGTCCTACACCGTATGTTTTTTGACGTCAGCTAGGCAAACCAACATAAATAGGAAGCAG 850
RG10               TTGTCCTACACCGTATGTTTTTTGACGTCAGCTAGGCAAACCAACATAAATAGGAAGCAG 852
                   ************************************************************

Glyma.14g194300    TAGAAGTAAAAGTAGAATGTGGTAGTGTTATTATTATTTACTACTGTTTCACCTTGGTGT 960
OX948              TGGAAGTAAAAGTAGAATGTGGTAGTGTTATTATTATTTACTACTGTTTCACCTTGGTGT 910
RG10               TAGAAGTAAAAGTAGAATGTGGTAGTGTTATTATTATTTACTACTGTTTCACCTTGGTGT 912
                   * **********************************************************

                     TATA-box
Glyma.14g194300    TATATAAATGCACTACCCCATAATTGAATTTTTCAAAGATTTCATTCTTCCTCTTCTAGG 1020 963-969
OX948              TATATAAATGCACTACCCCATAATTGAATTTTTCAAAGATTTCATTCTTCCTCTTCTAGG 970  913-917
RG10               TATATAAATGCACTACCCCATAATTGAATTTTTCAAAGATTTCATTCTTCCTCTTCTAGG 972  915-919
                   ************************************************************

Glyma.14g194300    TTATTACGCACCACCCACCACGTATCCCTGAAAAGAGAGAAAAACACACTAAGCCAAAGC 1080
OX948              TTATTACGCACCACCCACCACGTATCCCTGAAAAGAGAGAAAAACACACTAAGCCAAAGC 1030
RG10               TTATTACGCACCACCCACCACGTATCCCTGAAAAGAGAGAAAAACACACTAAGCCAAAGC 1032
                   ************************************************************

                             ATG                                                 EXON 1
Glyma.14g194300    CAAAGCAGCAATGGTTAAAGACACAAAGCCTTTAGCCTATGCTGCTAATAATGGATACCA 1140 1091
OX948              CAAAGCAGCAATGGTTAAAGACACAAAGCCTTTAGCCTATGCTGCTAATAATGGATACCA 1090 1041
RG10               CAAAGCAGCAATGGTTAAAGACACAAAGCCTTTAGCCTATGCTGCTAATAATGGATACCA 1092 1043
                   ************************************************************

Glyma.14g194300    AAAGGAAGCTTTTGATCCCAGTGCTCCTCCACCGTTTAAGATTGCAGAAATCAGAGTTGC 1200
OX948              AAAGGAAGCTTTTGATCCCAGTGCTCCTCCACCGTTTAAGATTGCAGAAATCAGAGTTGC 1150
RG10               AAAGGAAGCTTTTGATCCCAGTGCTCCTCCACCGTTTAAGATTGCAGAAATCAGAGTTGC 1152
                   ************************************************************

Glyma.14g194300    AATACCAAAACATTGCTGGGTCAAGAATCCATGGAGATCCCTCAGTTATGTTCTCAGGGA 1260
OX948              AATACCAAAACATTGCTGGGTCAAGAATCCATGGAGATCCCTCAGTTATGTTCTCAGGGA 1210
RG10               AATACCAAAACATTGCTGGGTCAAGAATCCATGGAGATCCCTCAGTTATGTTCTCAGGGA 1212
                   ************************************************************

Glyma.14g194300    TGTGCTTGTAATTGCTGCATTGATGGCTGCTGCAAGTCACTTCAACAACTGGCTTCTCTG 1320
OX948              TGTGCTTGTAATTGCTGCATTGATGGCTGCTGCAAGTCACTTCAACAACTGGCTTCTCTG 1270
RG10               TGTGCTTGTAATTGCTGCATTGATGGCTGCTGCAAGTCACTTCAACAACTGGCTTCTCTG 1272
                   ************************************************************
```

```
                                                                     H
Glyma.14g194300    GCTAATCTATTGGCCCATTCAAGGAACAATGTTCTGGGCTCTGTTTGTTCTTGGACATGA 1380
OX948              GCTAATCTATTGGCCCATTCAAGGAACAATGTTCTGGGCTCTGTTTGTTCTTGGACATGA 1330 H-box1
RG10               GCTAATCTATTGGCCCATTCAAGGAACAATGTTCTGGGCTCTGTTTGTTCTTGGACATGA 1332
                   ************************************************************

                                                                                 INTRON I
Glyma.14g194300    TTGgtaattaattaatttgttgttacttttttgttataatatgaatctcacacactgctt 1440
OX948              TTGgtaattaattaatttgttgttacttttttgttataatatgaatctcacacattgctt 1390
RG10               TTGgtaattaattaatttgttgttacttttttgttataatatgaatctcacacattgctt 1392
                   ****************************************************** *****

Glyma.14g194300    tgttatgcctacctcatttcatttggctttagacaacttaaatttgagatctttattatg 1500
OX948              tgttatgcctacctcatttcatttggctttagacaacttaaatttgagatctttattatg 1450
RG10               tgttatgcctacctcatttcatttggctttagacaacttaaatttgagatctttattatg 1452
                   ************************************************************

                                                                    H                EXON 2
Glyma.14g194300    ttttttgcttatatggtaaagtgattcattcttcacattgaattgaacagTGGCCATGGA 1560
OX948              ttttttgcttatatggtaaagtgattcattcttcacattgaattgaacagTGGCCATGGA 1510 H-box1
RG10               ttttttgcttatatggtaaagtgattcattcttcacattgaattgaacagTGGCCATGGA 1512
                   ************************************************************

Glyma.14g194300    AGCTTTTCAGACAGCCCTTTTCTAAATAGCCTGGTGGGACACATCTTGCATTCCTCAATT 1620
OX948              AGCTTTTCAGACAGCCCTTTTCTGAATAGCCTGGTGGGACACATCTTGCATTCCTCAATT 1570
RG10               AGCTTTTCAGACAGCCCTTTTCTAAATAGCCTGGTGGGACACATCTTGCATTCCTCAATT 1572
                   *********************** ************************************

                                                                                 INTRON 2
Glyma.14g194300    CTTGTGCCATACCATGGATGgttagttcatcccggctttttttgtttgtcattggaagttc 1680
OX948              CTTGTGCCATACCATGGATGgttagttcatcccggctttttttgtttgtcattggaagttc 1630
RG10               CTTGTGCCATACCATGGATGgttagttcatcccggctttttttgtttgtcattggaagttc 1632
                   ************************************************************

Glyma.14g194300    ttttattgattcaatttttatagcgtgttcggaaacgcgtttcagaaaataatgaaatac 1740
OX948              ttttattgattcaatttttatagcgtgttcggaaacgcgtttcagaaaataatgaaatac 1690
RG10               ttttattgattcaatttttatagcgtgttcggaaacgcgtttcagaaaataatgaaatac 1692
                   ************************************************************

Glyma.14g194300    atcttgaatctgaaagttataacttttagcttcattgtcattgaaagttcttttattaat 1800
OX948              atcttgaatctgaaagttataacttttagcttcattgtcattgaaagttcttttattaat 1750
RG10               atcttgaatctgaaagttataacttttagcttcattgtcattgaaagttcttttattaat 1752
                   ************************************************************

Glyma.14g194300    tatatttttattgcgtgtttggaatcccatttgagaaataagaaatcacgtttaaaatgt 1860
OX948              tatatttttattgcgtgtttggaatcccatttgagaaataagaaatcacgtttaaaatgt 1810
RG10               tatatttttattgcgtgtttggaatcccatttgagaaataagaaatcacgtttaaaatgt 1812
                   ************************************************************

Glyma.14g194300    gaaagttataactattaacttttgactaaacttgaaaaaatcacattttttgatgtggaac 1920
OX948              gaaagttataactattaacttttgactaaacttgaaaaaatcacattttttgatgtggaac 1870
RG10               gaaagttataactattaacttttgactaaacttgaaaaaatcacattttttgatgtggaac 1872
                   ************************************************************

                                                                    H                EXON 3
Glyma.14g194300    caaatctgatttgagaaccaagttgattttgatggattttgcagGAGAATTAGCCACAGA 1980
OX948              caaatctgatttgagaaccaagttgattttgatggattttgcagGAGAATTAGCCACAGA 1930 H-box2
RG10               caaatctgatttgagaaccaagttgattttgatggattttgcagGAGAATTAGCCACAGA 1932
                   ************************************************************

                       H  H                                                      INTRON 3
Glyma.14g194300    ACTCACCATCAAAATCATGGACACATTGAGAAGGATGAATCCTGGGTTCCAgtatgtgat 2040
OX948              ACTCACCATCAAAATCATGGACACATTGAGAAGGATGAATCCTGGGTTCCAgtatgtgat 1990
RG10               ACTCACCATCAAAATCATGGACACATTGAGAAGGATGAATCCTGGGTTCCAgtatgtgat 1992
                   ************************************************************
```

```
Glyma.14g194300    taactacttcctctatagttatttttgattcaattaaatttatttatttaataagttcaa 2100
OX948              taactacttcctctatagttatttttgattcaattaaatttatttatttaataagttcaa 2050
RG10               taactacttcctctatagttatttttgattcaattaaatttatttatttaataagttcaa 2052
                   ************************************************************

Glyma.14g194300    gaaaaaaggaatctttatacttca-gataaagctgttcttgaacattttttttt-gtcat 2158
OX948              gaaaaaaggaatctttatacttcatgataaagctgttcttgaacatttttttttttgtcat 2110
RG10               gaaaaaaggaatctttataccctca-gataaagctgttcttgaacattttttttt-gtcat 2110
                   ******************** *** ***************************** *****
                                                                                EXON 4
Glyma.14g194300    tatcttagTTAACCGAGAAGATTTACAAGAATCTAGACAACATGACAAGACTTGTTAGAT 2218
OX948              tatcttagTTAACCGAGAAGATTTACAAGAATCTAGACAACATGACAAGACTTGTTAGAT 2170
RG10               tatcttagTTAACCGAGAAGATTTACAAGAATCTAGACAACATGACAAGACTTGTTAGAT 2170
                   ************************************************************
                                                                               INTRON 4
Glyma.14g194300    TCACTGTGCCATTTCCATTGTTTGTGTATCCAATTTATTTGgtgagtgcttttttttttt 2278
OX948              TCACTGTGCCATTTCCATTGTTTGTGTATCCAATTTATTTGgtgagtgcttttttttttt 2230
RG10               TCACTGTGCCATTTCCATTGTTTGTGTATCCAATTTATTTGgtgagtgcttttttttttt 2230
                   ************************************************************

Glyma.14g194300    tacttggaagactacaacacattattattattataatatggttcaaatcaatgactttta 2338
OX948              tacttggaagactacaacacattattattattataatatggttcaaatcaatgactttta 2290
RG10               tacttggaagactacaacacattattattattataatatggttcaaatcaatgactttta 2290
                   ************************************************************
                                                                                EXON 5
Glyma.14g194300    atttctttgtgatgtgcactccattttcagTTCTCAAGAAGCCCCGGAAAGGAAGGTTCT 2398
OX948              atttctttgtgatgtgcactccattttcagTTCTCAAGAAGCCCCGGAAAGGAAGGTTCT 2350
RG10               atttctttgtgatgtgcactccattttcagTTCTCAAGAAGCCCCGGAAAGGAAGGTTCT 2350
                   ************************************************************

Glyma.14g194300    CACTTCAATCCCTACAGCAATCTGTTCCCACCCAGTGAGAGAAAGGGAATAGCAATATCA 2458
OX948              CACTTCAATCCCTACAGCAATCTGTTCCCACCCAGTGAGAGAAAGGGAATAGCAATATCA 2410
RG10               CACTTCAATCCCTACAGCAATCTGTTCCCACCCAGTGAGAGAAAGGGAATAGCAATATCA 2410
                   ************************************************************

Glyma.14g194300    ACACTGTGTTGGGTTACCATGTTTTCTATGCTTATCTATCTCTCCTTCATAACTAGTCCA 2518
OX948              ACACTGTGTTGGGTTACCATGTTTTCTATGCTTATCTATCTCTCCTTCATAACTAGTCCA 2470
RG10               ACACTGTGTTGGGTTACCATGTTTTCTATGCTTATCTATCTCTCCTTCATAACTAGTCCA 2470
                   ************************************************************
                                                                               INTRON 5
Glyma.14g194300    GTTCTATTGCTCAAGCTCTATGGAATTCCATATTGGgtaattaaattactcttacattac 2578
OX948              GTTCTATTGCTCAAGCTCTATGGAATTCCATATTGGgtaattaaattactcttacattac 2530
RG10               GTTCTATTGCTCAAGCTCTATGGAATTCCATATTGGgtaattaaattactcttacattac 2530
                   ************************************************************

Glyma.14g194300    tttttcctcttttttttttatgggtcttaactagtatcacaaaaatattggttaaaaaatt 2638
OX948              tttttcctcttttttttttatgggtcttaactagtatcacaaaaatattggttaaaaaatt 2590
RG10               tttttcctcttttttttttatgggtcttaactagtatcacaaaaatattggttaaaaaatt 2590
                   ************************************************************

Glyma.14g194300    ttaaaaaaatatttattatgtaaatcataaaagaacataaaaaaaatgatgaataacata 2698
OX948              ttaaaaaaatatttattatgtaaatcataaaagaacataaaaaaaatgatgaataacata 2650
RG10               ttaaaaaaatatttattatgtaaatcataaaagaacataaaaaaaatgatgaataacata 2650
                   ************************************************************

Glyma.14g194300    attttcgtctcttattaaaaatatttttatttaaatttcttaatcaatatatttagaat 2758
OX948              attttcgtctcttattaaaaatatttttatttaaatttcttaatcaatatatttagaat 2710
RG10               attttcgtctcttattaaaaatatttttatttaaatttcttaatcaatatatttagaat 2710
                   ************************************************************

Glyma.14g194300    ctggttaacatttttttgaatatttcaattctccaattaaaaatttgaaatagtcaccatt 2818
OX948              ctggttaacatttttttgaatatttcaattctccaattaaaaatttgaaatagtcaccatt 2770
RG10               ctggttaacatttttttgaatatttcaattctccaattaaaaatttgaaatagtcaccatt 2770
                   ************************************************************
```

```
                                                                                  EXON 6
Glyma.14g194300    aattatgtaattgtttgaacacgtgcagATATTTGTTATGTGGCTGGACTTTGTCACATA 2878
OX948              aattatgtaattgtttgaacacgtgcagATATTTGTTATGTGGCTGGACTTTGTCACATA 2830
RG10               aattatgtaattgtttgaacacgtgcagATATTTGTTATGTGGCTGGACTTTGTCACATA 2830
                   ************************************************************

                                                   *M Fad3A mutation             INTRON 6
Glyma.14g194300    CTTGCATCACCATGGTCATCATCAGAAACTGCCTTGGTATCGCGGCAAGgtaacaaaaat 2938
OX948              CTTGCATCACCATGGTCATCATCAGAAACTGCCTTGGTATCGCGGCAAGgtaacaaaaat 2890
RG10               CTTGCATCACCATGGTCATCATCAGAAACTGCCTTGATATCGCGGCAAGgtaacaaaaat 2890
                   ************************************ ***********************

Glyma.14g194300    aaatagaaaatagtgagtgaacacttaaatgttagatactaccttcttcttcttttttttt 2998
OX948              aaatagaaaatagtgagtgaacacttaaatgttagatactaccttcttcttcttttttttt 2950
RG10               aaatagaaaatagtgagtgaacacttaaatgttagatactaccttcttcttcttttttttt 2950
                   ************************************************************

glyma.14g194300    ttttttttgaggttaatgctagataatagctagaaagagaaagaaagacaaatataggta 3058
ox948              ttt---ttgaggttaatgctagataatagctagaaagagaaagaaagacaaatataggta 3007
rg10               ttttt-ttgaggttaatgctagataatagctagaaagagaaagaaagacaaatataggta 3009
                   ***   ******************************************************

Glyma.14g194300    aaaataaataatataacctgggaagaagaaaacataaaaaaagaaataatagagtctacg 3118
OX948              aaaataaataatataacctgggaagaagaaaacataaaaaaagaaataatagagtctacg 3067
RG10               aaaataaataatataacctgggaagaagaaaacataaaaaaagaaataatagagtctacg 3069
                   ************************************************************

Glyma.14g194300    taatgtttggatttttgagtgaaatggtgttcacctaccattactcaaagattctgttgt 3178
OX948              taatgtttggatttttgagtgaaatggtgttcacctaccattactcaaagattctgttgt 3127
RG10               taatgtttggatttttgagtgaaatggtgttcacctaccattactcaaagattctgttgt 3129
                   ************************************************************

Glyma.14g194300    ctacgtagtgtttggactttggagtgaaatggtgttcacctaccattactcagattctgt 3238
OX948              ctacgtagtgtttggactttggagtgaaatggtgttcacctaccattactcagattctgt 3187
RG10               ctacgtagtgtttggactttggagtgaaatggtgttcacctaccattactcagattctgt 3189
                   ************************************************************

Glyma.14g194300    tgtgtcccttagttactgtcttatattcttagggtatattctttattttacatccttttc 3298
OX948              tgtgtcccttagttactgtcttatattcttagggtatattctttattttacatccttttc 3247
RG10               tgtgtcccttagttactgtcttatattcttagggtatattctttattttacatccttttc 3249
                   ************************************************************

Glyma.14g194300    acatcttacttgaaaagattttaattattcattgaaatattaacgtgacagttaaatta 3358
OX948              acatcttacttgaaaagattttaattattcattgaaatattaacgtgacagttaaatta 3307
RG10               acatcttacttgaaaagattttaattattcattgaaatattaacgtgacagttaaatta 3309
                   ************************************************************

Glyma.14g194300    aaataataaaaaattcgttaaaacttcaaataaataagagtgaaaggatcatcatttttc 3418
OX948              aaataataaaaaattcgttaaaacttcaaataaataagagtgaaaggatcatcatttttc 3367
RG10               aaataataaaaaattcgttaaaacttcaaataaataagagtgaaaggatcatcatttttc 3369
                   ************************************************************

Glyma.14g194300    ttctttcttttattgcgttattaatcatgcttctcttcttttttttcttcgctttccacc 3478
OX948              ttctttcttttattgcgttattaatcatgcttctcttcttttttttcttcgctttccacc 3427
RG10               ttctttcttttattgcgttattaatcatgcttctcttcttttttttcttcgctttccacc 3429
                   ************************************************************

Glyma.14g194300    catatcaaattcatgtgaagtatgagaaaatcacgattcaatggaaagctacaggaactt 3538
OX948              catatcaaattcatgtgaagtatgagaaaatcacgattcaatggaaagctacaggaactt 3487
RG10               catatcaaattcatgtgaagtatgagaaaatcacgattcaatggaaagctacaggaactt 3489
                   ************************************************************

Glyma.14g194300    tttttgtttgtttttataatcggaattaatttatactccatttttcacaataaatgtt 3598
OX948              tttttgtttgtttttataatcggaattaatttatactccatttttcacaataaatgtt 3547
RG10               tttttgtttgtttttataatcggaattaatttatactccatttttcacaataaatgtt 3549
                   ************************************************************
```

```
Glyma.14g194300    acttagtgccttaaagataatatttgaaaaattaaaaaaattattaatacactgtactac 3658
OX948              acttagtgccttaaagataatatttgaaaaattaaaaaaattattaatacactgtactac 3607
RG10               acttagtgccttaaagataatatttgaaaaattaaaaaaattattaatacactgtactac 3609
                   ************************************************************

Glyma.14g194300    tatataatatttgacatatatttaacatgattttctattgaaaatttgtatttattattt 3718
OX948              tatataatatttgacatatatttaacatgattttctattgaaaatttgtatttattattt 3667
RG10               tatataatatttgacatatatttaacatgattttctattgaaaatttgtatttattattt 3669
                   ************************************************************

Glyma.14g194300    tttaatcaaaacccataaggcattaatttacaagacccatttttcatttatagctttacc 3778
OX948              tttaatcaaaacccataaggcattaatttacaagacccatttttcatttatagctttacc 3727
RG10               tttaatcaaaacccataaggcattaatttacaagacccatttttcatttatagctttacc 3729
                   ************************************************************

Glyma.14g194300    tgtgatcatttatagctttaagggacttagatgttacaatcttaattacaagtaaatatt 3838
OX948              tgtgatcatttatagctttaagggacttagatgttacaatcttaattacaagtaaatatt 3787
RG10               tgtgatcatttatagctttaagggacttagatgttacaatcttaattacaagtaaatatt 3789
                   ************************************************************

Glyma.14g194300    tatgaaaaacatgtgtcttaccccttaaccttacctcaacaaagaaagtgtgataagtgg 3898
OX948              tatgaaaaacatgtgtcttaccccttaaccttacctcaacaaagaaagtgtgataagtgg 3847
RG10               tatgaaaaacatgtgtcttaccccttaaccttacctcaacaaagaaagtgtgataagtgg 3849
                   ************************************************************

Glyma.14g194300    caacacacgtgttgctttttttggcccagcaataacacgtgtttttgtggtgtacaaaaat 3958
OX948              caacacacgtgttgctttttttggcccagcaataacacgtgtttttgtggtgtacaaaaat 3907
RG10               caacacacgtgttgctttttttggcccagcaataacacgtgtttttgtggtgtacaaaaat 3909
                   ************************************************************
                                                                                    EXON 7
Glyma.14g194300    ggacagGAATGGAGTTATTTAAGAGGTGGTCTCACAACTGTGGATCGTGACTATGGTTGG 4018
OX948              ggacagGAATGGAGTTATTTAAGAGGTGGTCTCACAACTGTGGATCGTGACTATGGTTGG 3967
RG10               ggacagGAATGGAGTTATTTAAGAGGTGGTCTCACAACTGTGGATCGTGACTATGGTTGG 3969
                   ************************************************************

                                                 H         H  H
Glyma.14g194300    ATCAATAACATTCACCATGACATTGGCACCCATGTTATTCACCATCTTTTCCCTCAAATT 4078
OX948              ATCAATAACATTCACCATGACATTGGCACCCATGTTATTCACCATCTTTTCCCTCAAATT 4027 H-box3
RG10               ATCAATAACATTCACCATGACATTGGCACCCATGTTATTCACCATCTTTTCCCTCAAATT 4029
                   ************************************************************
                                                                                   INTRON 7
Glyma.14g194300    CCTCATTATCACCTCGTTGAAGCGgtatattttactattattactcacctaaaaagaatg 4138
OX948              CCTCATTATCACCTCGTTGAAGCGgtatattttactattattactcacctaaaaagaatg 4087
RG10               CCTCATTATCACCTCGTTGAAGCGgtatattttactattattactcacctaaaaagaatg 4089
                   ************************************************************

Glyma.14g194300    caattagtacatttgttttatctcttggaagttagtcattttcagttgcatgattgtaat 4198
OX948              caattagtacatttgttttatctcttggaagttagtcattttcagttgcatgattgtaat 4147
RG10               caattagtacatttgttttatctcttggaagttagtcattttcagttgcatgattgtaat 4149
                   ************************************************************

Glyma.14g194300    gttctctctatttttaaaccatgttttcacacctacttcgtttaaaataagaatgtggat 4258
OX948              gttctctctatttttaaaccatgttttcacacctacttcgtttaaaataagaatgtggat 4207
RG10               gttctctctatttttaaaccatgttttcacacctacttcgtttaaaataagaatgtggat 4209
                   ************************************************************

Glyma.14g194300    actattctaatttctattaacttcttttaaaaaataatgtaaaactagtattaaaaaaga 4318
OX948              actattctaatttctattaacttcttttaaaaaataatgtaaaactagtattaaaaaaga 4267
RG10               actattctaatttctattaacttcttttaaaaaataatgtaaaactagtattaaaaaaga 4269
                   ************************************************************

Glyma.14g194300    ggaaatagattacactctactaatactaatagtataaaaaaaattacattgttattttat 4378
OX948              ggaaatagattacactctactaatactaatagtataaaaaaaattacattgttattttat 4327
RG10               ggaaatagattacactctactaatactaatagtataaaaaaaattacattgttattttat 4329
                   ************************************************************
```

```
Glyma.14g194300    cacaaataattatatataattaatttttacaatcattatcttaaaagtcatgtatgatat 4438
OX948              cacaaataattatatataattaatttttacaatcattatcttaaaagtcatgtatgatat 4387
RG10               cacaaataattatatataattaatttttacaatcattatcttaaaagtcatgtatgatat 4389
                   ************************************************************

Glyma.14g194300    acagtttttacatgctttggtacttattgtaaagttagtgatttattcattatttatgtt 4498
OX948              acagtttttacatgctttggtacttattgtaaagttagtgatttattcattatttatgtt 4447
RG10               acagtttttacatgctttggtacttattgtaaagttagtgatttattcattatttatgtt 4449
                   ************************************************************

Glyma.14g194300    atataattggcataaatatcatgtaaccagctcactatactataatgggaacttggtggt 4558
OX948              atataattggcataaatatcatgtaaccagctcactatactataatgggaacttggtggt 4507
RG10               atataattggcataaatatcatgtaaccagctcactatactataatgggaacttggtggt 4509
                   ************************************************************

Glyma.14g194300    gaaaggggtttacaaccctcttttctaggtgtaggtgctttgatacttctggtccctttt 4618
OX948              gaaaggggtttacaaccctcttttctaggtgtaggtgctttgatacttctggtccctttt 4567
RG10               gaaaggggtttacaaccctcttttctaggtgtaggtgctttgatacttctggtccctttt 4569
                   ************************************************************

Glyma.14g194300    tatatcaatataaattatattttgctgataaaaaaaacattattaatatataatcattaa 4678
OX948              tatatcaatataaattatattttgctgataaaaaaaacattattaatatataatcattaa 4627
RG10               tatatcaatataaattatattttgctgataaaaaaaacattattaatatataatcattaa 4629
                   ************************************************************

Glyma.14g194300    cttctttaaaaaccgtacctaaaactttatattattaaaaagaagattgagatcagcaaa 4738
OX948              cttctttaaaaaccgtacctaaaactttatattattaaaaagaagattgagatcagcaaa 4687
RG10               cttctttaaaaaccgtacctaaaactttatattattaaaaagaagattgagatcagcaaa 4689
                   ************************************************************
                                                                                  EXON 8
Glyma.14g194300    agaaaaaaaaaattaacagtcatttgaattcactgcagACACAAGCAGCAAAATCAGTTCT 4798
OX948              agaaaaaaaaaattaacagtcatttgaattcactgcagACACAAGCAGCAAAATCAGTTCT 4747
RG10               agaaaaaaaaaattaacagtcatttgaattcactgcagACACAAGCAGCAAAATCAGTTCT 4749
                   ************************************************************

Glyma.14g194300    TGGAGAGTATTACCGTGAGCCAGAAAGATCTGCACCATTACCATTTCATCTAATAAAGTA 4858
OX948              TGGAGAGTATTACCGTGAGCCAGAAAGATCTGCACCATTACCATTTCATCTAATAAAGTA 4807
RG10               TGGAGAGTATTACCGTGAGCCAGAAAGATCTGCACCATTACCATTTCATCTAATAAAGTA 4809
                   ************************************************************

Glyma.14g194300    TTTAATTCAGAGTATGAGACAAGACCACTTCGTAAGTGACACTGGAGATGTGGTTTATTA 4918
OX948              TTTAATTCAGAGTATGAGACAAGACCACTTCGTAAGTGACACTGGAGATGTGGTTTATTA 4867
RG10               TTTAATTCAGAGTATGAGACAAGACCACTTCGTAAGTGACACTGGAGATGTGGTTTATTA 4869
                   ************************************************************
                                                                                 3'UTR
Glyma.14g194300    TCAGACTGATTCTCTGCACCTTCACTCGCACCGAGACTGAGTTTCAATTTTTGGGTTATT 4978 4958
OX948              TCAGACTGATTCTCTGCACCTTCACTCGCACCGAGACTGAGTTTCAATTTTTGGGTTATT 4927 4907
RG10               TCAGACTGATTCTCTGCACCTTCACTCGCACCGAGACTGAGTTTCAATTTTTGGGTTATT 4929 4909
                   ************************************************************

Glyma.14g194300    TATTGGATTCTAGCTACTCAAATTACTTTTTTTTTAATGTTACGTTTTTGGAGTTTTAAC 5038
OX948              TATTGGATTCTAGCTACTCAAATTACTTTTTTTTTAATGTTACGTTTTTGGAGTTTTAAC 4987
RG10               TATTGGATTCTAGCTACTCAAATTACTTTTTTTTTAATGTTACGTTTTTGGAGTTTTAAC 4989
                   ************************************************************

Glyma.14g194300    GTTTTCTGAACAACTTGCAAATTACATGCATAGAGAGACAGGAATTCATAGTGGGCCTCA 5098
OX948              GTTTTCTGAACAACTTGCAAATTACATGCATAGAGAGACAGGAATTCATAGTGGGCCTCA 5047
RG10               GTTTTCTGAACAACTTGCAAATTACATGCATAGAGAGACAGGAATTCATAGTGGGCCTCA 5049
                   ************************************************************

                                                        Poly (A)
Glyma.14g194300    ATGGAATATTTATTTGAAATTAGTAAGGTGGTAATTAATAAATATTGAATTGTCAGTTTC 5158 5135-5140
OX948              ATGGAATATTTATTTGAAATTAGTAAGGTGGTAATTAATAAATATTGAATTGTCAGTTTC 5107 5084-5089
RG10               ATGGAATATTTATTTGAAATTAGTAAGGTGGTAATTAATAAATATTGAATTGTCAGTTTC 5109 5086-5091
                   ************************************************************
```

```
Glyma.14g194300    ATTTGTAATGATTTCTACCACTTGCACAGAGCATTCACATGGGCAACATACATTTAAAAA 5218
OX948              ATTTGTAATGATTTCTACCACTTGCACAGAGCATTCACATGGGCAACATACATTTAAAAA 5167
RG10               ATTTGTAATGATTTCTACCACTTGCACAGAGCATTCACATGGGCAACATACATTTAAAAA 5169
                   ************************************************************

Glyma.14g194300    AATTATTACATAAGGCAGTGTTTCTATTATTTAATCGAGAATTTAACAGCAACTATGCAA 5278
OX948              AATTATTACATAAGGCAGTGTTTCTATTATTTAATCGAGAATTTAACAGCAACTATGCAA 5227
RG10               AATTATTACATAAGGCAGTGTTTCTATTATTTAATCGAGAATTTAACAGCAACTATGCAA 5229
                   ************************************************************

Glyma.14g194300    GAAAATTACGTGATTTTGGGAAAGTTTCAACCCAAAGTTTGAACAAAAATTTGTCTCTAA 5338
OX948              GAAAATTACGTGATTTTGGGAAAGTTTCAACCCAAAGTTTGAACAAAAATTTGTCTCTAA 5287
RG10               GAAAATTACGTGATTTTGGGAAAGTTTCAACCCAAAGTTTGAACAAAAATTTGTCTCTAA 5289
                   ************************************************************

Glyma.14g194300    GTTATGATTTAGAACTAATTCTATAGGTGAAACTATCAGAAACAGAGTTCCACCGTTGAA 5398
OX948              GTTATGATTTAGAACTAATTCTATAGGTGAAACTATCAGAAACAGAGTTCCACCGTTGAA 5347
RG10               GTTATGATTTAGAACTAATTCTATAGGTGAAACTATCAGAAACAGAGTTCCACCGTTGAA 5349
                   ************************************************************

Glyma.14g194300    GATCTGTTGTACCTAACTGTCAAAAAAAAAAGATCCGTTGACCTGTAAGTAAAGTCACTG 5458
OX948              GATCTGTTGTACCTAACTGTCAAAAAAAAAAGATCCGTTGACCTGTAAGTAAAGTCACTG 5407
RG10               GATCTGTTGTACCTAACTGTCAAAAAAAAAAGATCCGTTGACCTGTAAGTAAAGTCACTG 5409
                   ************************************************************

Glyma.14g194300    TAGAAGAAGTGGGTCAAGTTGTACAGTAAAGGCAAAAATAAAAATAAGTCTTGATGCATA 5518
OX948              TANAAAAAGTGGGTCAAGTTGTACAGTAAAGGCAAAAATAAAAATAANTCTNGANGCATA 5467
RG10               TAGAAGAAGTGGGTCAAGTTGTACAGTAAAGGCAAAAATAAAAATAAGTCTTGATGCATA 5469
                   ** ** ***************************************** *** ** *****

Glyma.14g194300    AATATAAGAAATTTATTGTCAAAGTTTTTATTATTTTTTTTAAATTGTATAATTAAATGA 5578
OX948              AATATAANAAATTNATTGNCAAAGTTTTTATTATTTTTTTTAAATTGNATAATTAAATGA 5527
RG10               AATATAAGAAATTTATTGTCAAAGTTTTTATTATTTTTTTTAAATTGTATAATTAAATGA 5529
                   ******* ***** **** **************************** ************

Glyma.14g194300    AAAAATTGCTTAAAATATAAAAAATGTACGTTATAAGATCTATTTAATAATTATTAAATA 5638
OX948              AAAAATTGCTTAAAATATAAAAAATGNNCGTTATAAGATCTATTTAATAATTATTAAATA 5587
RG10               AAAAATTGCTTAAAATATAAAAAATGTACGTTATAAGATCTATTTAATAATTATTAAATA 5589
                   **************************  ********************************

Glyma.14g194300    ATGTACGGATATAATATCTATTTAATAATTATTTAAATAATTAAGTAATATGGTGGTTGG 5698
OX948              ATGTACGGATATAATATCTATTTAATAATTATTTAAANAATTAAGTAATATGGNGGTTGG 5647
RG10               ATGTACGGATATAATATCTATTTAATAATTATTTAAATAATTAAGTAATATGGTGGTTGG 5649
                   ************************************* *************** ******

Glyma.14g194300    ATGTGCGCATTTAAGGTAGTAGTGTAGTAGAAAATTAAGAGCATGGTGACTATTCATGCA 5758
OX948              ATGTGCGCATTTAAGGTAGTAGNGNAGTANAAAATTANNANCATGGTGACTATTCATGCA 5707
RG10               ATGTGCGCATTTAAGGTAGTAGTGTAGTAGAAAATTAAGAGCATGGTGACTATTCATGCA 5709
                   ********************** * **** *******  * *******************

Glyma.14g194300    TAACTAGGTTTCTGAAGAAAGACTCCGAAATGAACACAAGCCAAATTCATTGAATGAAAG 5818
OX948              TAACTAGGTTTCTGAAAAAAGACTCCGAAATGANNNNNAGCNANNTTCNTTGAATGA--- 5764
RG10               TAACTAGGTTTCTGAAGAAAGACTCCGAAATGAACACAAGCCAAATTCATTGAATGA--- 5766
                   **************** ****************     *** *  *** ********

Glyma.14g194300    AAGTGTTCCAAAAGAACACGTTTGTTGGTATGCTCACCCTGAGGTCAATAATGATGGTAG 5878
OX948              ------------------------------------------------------------
RG10               ------------------------------------------------------------

Glyma.14g194300    GATGGAATATTTTATTTTTCTCACATATTTTTTTAATGGCCTTGTATAATTAAACAATAA 5938
OX948              ------------------------------------------------------------
RG10               ------------------------------------------------------------
```

```
Glyma.14g194300    ATATAATATTTTTTTTAACAAAAAATTGAATATTAATTCATCTCATAAAGGGTCTTAGGG 5998
OX948              ------------------------------------------------------------
RG10               ------------------------------------------------------------

Glyma.14g194300    AGGAAGTCAAGACGCTTGCTTGTTCCCTT 6027
OX948              -----------------------------
RG10               -----------------------------
```

FIGURE S2 | Comparison of *FAD3B* gene sequences in Williams 82 (Glyma.02g227200, Wm82.a2.v1), RG10 (KU310963) and OX948 (KU310959); exon sequences are shown in capital letters and intron sequences are shown in small letters; sequence differences are highlighted; mutation (*M) in *Fad3B* gene in line RG10 is shown in red.

```
                                                                                 5'UTR
Glyma.02g227200    TAAATTACAAAGTGAGTTTTACACTGTATCTATTCATAAAACACCATGATAAGTTTGAGA 60
OX948              ------------------------------------------------------------
RG10               ------------------------------------------------------------

Glyma.02g227200    TCATGACTGTCTACAGTGGAAAAGCAACCGCAGGGTCCTACAGTCACAATCCAAAGGTTG 120
OX948              ---TGACTGTCTACAGTGGAAAAGCAACCGCAGGGTCCTACAGTCACAATCCAAAGGTTG 57
RG10               -CATGACTGTCTACAGTGGAAAAGCAACCGCAGGGTCCTACAGTCACAATCCAAAGGTTG 59
                      *********************************************************

Glyma.02g227200    ATTTTTTTTTTCACTTATATTTTGTTTAATTATTTAATTTTTATATTTTACTTTTTAAAG 180
OX948              ATTTTTTTTTTCACTTATATTTTGTTTAATTATTTAATTTTTATATTTTACTTTTTAAAG 117
RG10               ATTTTTTTTTTCACTTATATTTTGTTTAATTATTTAATTTTTATATTTTACTTTTTAAAG 119
                   ************************************************************

Glyma.02g227200    GTGGAAATTTCATGCTATCACTTTTGTGACGATAAAGAATTCATTTTATAAGTTTATTAA 240
OX948              GTGGAGATTTCATGCTATCACTTTTGTGACGATAAAGAATTCATTTTATAAGTTTATTAA 177
RG10               GTGGAAATTTCATGCTATCACTTTTGTGACGATAAAGAATTCATTTTATAAGTTTATTAA 179
                   ***** ******************************************************

Glyma.02g227200    TTTTTATAATAATTATCTAAAAAATTACATCAATAATAATTTTTGTTTTTTTAGAGAAAA 300
OX948              TTTTTATAATAATTATCTAAAAAATTACATCAATAATAATTTTTGTTTTTTTAGAGAAAA 237
RG10               TTTTTATAATAATTATCTAAAAAATTACATCAATAATAATTTTTGTTTTTTTAGAGAAAA 239
                   ************************************************************

Glyma.02g227200    ATAATTTTCTATTAGCTGATAGCTTAAAATAATTTTATACATAATATAATATATGACTAT 360
OX948              ATAATTTTCTATTAGCTGATAGCTTAAAATAATTTTATACATAATATAATATATGACTAT 297
RG10               ATAATTTTCTATTAGCTGATAGCTTAAAATAATTTTATACATAATATAATATATGACTAT 299
                   ************************************************************

Glyma.02g227200    TAAATTTTAAAAAATAAACACATTTCAATATCTTCTAAAGTACTAGAGAAATAATTCGAA 420
OX948              TAAATTTTAAAAAATAAACACATTTCAATATCTTCTAAAGTACTAGAGAAATAATTCGAA 357
RG10               TAAATTTTAAAAAATAAACACACTTCAATATCTTCTAAAGTACTAGAGAAATAATTCGAA 359
                   ********************** *************************************

Glyma.02g227200    TTGAATTTAACGAAAAAGTTTTATAATTTTTTTTAGTGCATACTCTCACAATTGTTTCAT 480
OX948              TTGAATTTAACGAAAAAGTTTTATAATTTTTTTTAGTGCATACTCTCACAATTGTTTCAT 417
RG10               TTGAATTTAACGAAAAAGTTTTATAATTTTTTTTAGTGCATACTCTCACAATTGTTTCAT 419
                   ************************************************************

Glyma.02g227200    TATTTTTGTTGGAAGCAATTAATTACTTGCACAATTTTTATTTGGGGGTCATAGTTAATT 540
OX948              TATTTTTGTTGGAAGCAATTAATTACTTGCACAATTTTTATTTGGGGGTCATAGTTAATT 477
RG10               TATTTTTGTTGGAAGCAATTAATTACTTGCACAATTTTTATTTGGGGGTCATAGTTAATT 479
                   ************************************************************

Glyma.02g227200    ACCACTACAACCAACCGTAATTGGGTTGTTCCATTCCGTGACTAAAGTACTAAATAATAA 600
OX948              ACCACTACAACCAACCGTAATTGGGTTGTTCCATTCCGTGACTACAGTACTAAATAATAA 537
RG10               ACCACTACAACCAACCGTAATTGGGTTGTTCCATTCCGTGACTAAAGTACTAAATAATAA 539
                   ******************************************** ***************

Glyma.02g227200    ATAAATAAATAAAACAAAACAAGATAATTCAACTTCAATTATTAATTTAGTTCTTAGTAA 660
OX948              ATAAATAAAAAAAA-AAAACAAGATAATTCAACTTCAATTATTAATTTAGTTCTTAGTAA 596
RG10               ATAAATAAATAAAACAAAACAAGATAATTCAACTTCAATTATTAATTTAGTTCTTAGTAA 599
                   ********* **** *********************************************

Glyma.02g227200    AACTTAAGAGCTCAAGATCAAATTAAAATAATTTTGATATTTTTGTTTAATTATATGATA 720
OX948              AACTTAAGAGCTCAAGATCAAATTAAAATAATTTTGATATTTTTGTTTAATTATATGATA 656
RG10               AACTTAAGAGCTCAAGATCAAATTAAAATAATTTTGATATTTTTGTTTAATTATATGATA 659
                   ************************************************************
```

```
Glyma.02g227200    GGGATTTAATTTTTCTGTCTAAGATATATTCCTGATTCTTTTTTTGTGACGTTAAATAGG 780
OX948              GGGATTTAATTTTTCTGTCTAAGATATATTCCTGATTCTTTTTTTGTGACGTTAAATAGG 716
RG10               GGGATTTAATTTTTCTGTCTAAGATATATTCCTGATTCTTTTTTTGTGACGTTAAATAGG 719
                   ************************************************************

Glyma.02g227200    AAATAGGGGGAGGAAGCAGTAGAAGTAAAACAAACTCTAATGTGGTAGTGTTGTTATTAT 840
OX948              AAATAGGGGGAGGAAGCAGTAGAAGTAAAACAAACTCTAATGTGGTAGTGTTGTTATTAT 776
RG10               AAATAGGGGGAGGAAGCAGTAGAAGTAAAACAAACTCTAATGTGGTAGTGTTGTTATTAT 779
                   ************************************************************

                                               TATA-box
Glyma.02g227200    TTACTGCCAGTGTTTCACCCTGCGGTTATATAAATGCACTATCCCATAAGAGTATTTTTC 900  869-874
OX948              TTACTGCCAGTGTTTCACCCTGCGGTTATATAAATGCACTATCCCATAAGAGTATTTTTC 836  805-810
RG10               TTACTGCCAGTGTTTCACCCTGCGGTTATATAAATGCACTATCCCATAAGAGTATTTTTC 839  808-813
                   ************************************************************

Glyma.02g227200    GAAGATTTCCTTCTTCCTATTCTAGGTTTTTACGCACCACGTATCCCTGAGAAAAGAGAG 960
OX948              GAAGATTTCCTTCTTCCTATTCTAGGTTTTTACGCACCACGTATCCCTGAGAAAAGAGAG 896
RG10               GAAGATTTCCTTCTTCCTATTCTAGGTTTTTACGCACCACGTATCCCTGAGAAAAGAGAG 899
                   ************************************************************

                                                           ATG                 EXON 1
Glyma.02g227200    GAACCACACTCTCTAAGCCAAAGCAAAAGCAGCAGCAGCAGCAATGGTTAAAGACACAAA 1020 1004
OX948              GAACCACACTCTCTAAGCCAAAGCAAAAGCAGCAGCAGCAGCAATGGTTAAAGACACAAA 956  940
RG10               GAACCACACTCTCTAAGCCAAAGCAAAAGCAGCAGCAGCAGCAATGGTTAAAGACACAAA 959  943
                   ************************************************************

Glyma.02g227200    GCCTTTAGCCTATGCTGCCAATAATGGATACCAACAAAAGGGTTCTTCTTTTGATTTTGA 1080
OX948              GCCTTTAGCCTATGCTGCCAATAATGGATACCAACAAAAGGGTTCTTCTTTTGATTTTGA 1016
RG10               GCCTTTAGCCTATGCTGCCAATAATGGATACCAACAAAAGGGTTCTTCTTTTGATTTTGA 1019
                   ************************************************************

Glyma.02g227200    TCCTAGCGCTCCTCCACCGTTTAAGATTGCAGAAATCAGAGCTTCAATACCAAAACATTG 1140
OX948              TCCTAGCGCTCCTCCACCGTTTAAGATTGCAGAAATCAGAGCTTCAATACCAAAACATTG 1076
RG10               TCCTAGCGCTCCTCCACCGTTTAAGATTGCAGAAATCAGAGCTTCAATACCAAAACATTG 1079
                   ************************************************************

Glyma.02g227200    CTGGGTCAAGAATCCATGGAGATCCCTCAGTTATGTTCTCAGGGATGTGCTTGTAATTGC 1200
OX948              CTGGGTCAAGAATCCATGGAGATCCCTCAGTTATGTTCTCAGGGATGTGCTTGTAATTGC 1136
RG10               CTGGGTCAAGAATCCATGGAGATCCCTCAGTTATGTTCTCAGGGATGTGCTTGTAATTGC 1139
                   ************************************************************

Glyma.02g227200    TGCATTGGTGGCTGCAGCAATTCACTTCGACAACTGGCTTCTCTGGCTAATCTATTGCCC 1260
OX948              TGCATTGGTGGCTGCAGCAATTCACTTCGACAACTGGCTTCTCTGGCTAATCTATTGCCC 1196
RG10               TGCATTGGTGGCTGCAGCAATTCACTTCGACAACTGGCTTCTCTGGCTAATCTATTGCCC 1199
                   ************************************************************

                                                        H                      INTRON 1
Glyma.02g227200    CATTCAAGGCACAATGTTCTGGGCTCTCTTTGTTCTTGGACATGATTGgtaataattttt 1320
OX948              CATTCAAGGCACAATGTTCTGGGCTCTCTTTGTTCTTGGACATGATTGgtaataattttt 1256 H-box1
RG10               CATTCAAGGCACAATGTTCTGGGCTCTCTTTGTTCTTGGACATGATTGgtaataattttt 1259
                   ************************************************************

Glyma.02g227200    gtgtttcttactcttttttttttttt--ttgtttatgatatgaatctcacacattgttc 1378
OX948              gtgtttcttactctttttttttttttttttgtttatgatatgaatctcacacattgttc 1316
RG10               gtgtttcttactcttttttttttt------ttgtttatgatatgaatctcacacattgttc 1313
                   ***********************      *******************************

Glyma.02g227200    tgttatgtcatttcttcttcatttggctttagacaacttaaatttgagatctttattatg 1438
OX948              tgttatgtcatttcttcttcatttggctttagacaacttaaatttgagatctttattatg 1376
RG10               tgttatgtcatttcttcttcatttggctttagacaacttaaatttgagatctttattatg 1373
                   ************************************************************
```

```
Glyma.02g227200    tttttgcttatatggtaaagtgattcttcattatttcattcttcattgattgaattgaac 1498
OX948              tttttgcttatatggtaaagtgattcttcattatttcattcttcattgattgaattgaac 1436
RG10               tttttgcttatatggtaaagtgattcttcattatttcattcttcattgattgaattgaac 1433
                   ************************************************************

                        H                                                        EXON 2
Glyma.02g227200    agTGGCCATGGAAGCTTTTCAGATAGCCCTTTGCTGAATAGCCTGGTGGGACACATCTTG 1558
OX948              agTGGCCATGGAAGCTTTTCAGATAGCCCTTTGCTGAATAGCCTGGTGGGACACATCTTG 1496 H-box1
RG10               agTGGCCATGGAAGCTTTTCAGATAGCCCTTTGCTGAATAGCCTGGTGGGACACATCTTG 1493
                   ************************************************************
                                                                                 INTRON 2
Glyma.02g227200    CATTCCTCAATTCTTGTGCCATACCATGGATGgttagttcatactggctttttttgtttgt 1618
OX948              CATTCCTCAATTCTTGTGCCATACCATGGATGgttagttcatactggctttttttgtttgt 1556
RG10               CATTCCTCAATTCTTGTGCCATACCATGGATGgttagttcatactggctttttttgtttgt 1553
                   ************************************************************

Glyma.02g227200    tcatttgtcattgaaaaaaaatcttttgttgattcaattatttttatagtgtgtttggaa 1678
OX948              tcatttgtcattgaaaaaaaatcttttgttgattcaattatttttatagtgtgtttggaa 1616
RG10               tcatttgtcattgaaaaaaaatcttttgttgattcaattatttttatagtgtgtttggaa 1613
                   ************************************************************

Glyma.02g227200    gcccgtttgagaaaataagaaatcgcatctggaatgtgaaagttataactatttagcttc 1738
OX948              gcccgtttgagaaaataagaaatcgcatctggaatgtgaaagttataactatttagcttc 1676
RG10               gcccgtttgagaaaataagaaatcgcatctggaatgtgaaagttataactatttagcttc 1673
                   ************************************************************

Glyma.02g227200    atctgtcgttgcaagttcttttattggttaaatttttatagcgtgctaggaaacccattc 1798
OX948              atttgtcgttgcaagttcttttattggttaaatttttatagcgtgctaggaaacccattc 1736
RG10               atttgtcgttgcaagttcttttattggttaaatttttatagcgtgctaggaaacccattc 1733
                   ** *********************************************************

Glyma.02g227200    gagaaaataagaaatcacatctggaatgtgaaagttataactgttagcttctgagtaaac 1858
OX948              gagaaaataagaaatcacatctggaatgtgaaagttattactgttagcttctgagtaaac 1796
RG10               gagaaaataagaaatcacatctggaatgtgaaagttattactgttagcttctgagtaaac 1793
                   ************************************** *********************

Glyma.02g227200    gtggaaaaaccacattttggatttggaaccaaattttatttgataaatgacaaccaaatt 1918
OX948              gtggaaaaaccacattttggatttggaaccaaattttatttgataaatgacaaccaaatt 1856
RG10               gtggaaaaaccacattttggatttggaaccaaattttatttgataaatgacaaccaaatt 1853
                   ************************************************************

                                               H        H  H                     EXON 3
Glyma.02g227200    gattttgatggattttgcagGAGAATTAGCCACAGAACTCACCATCAAAACCATGGACAC 1978
OX948              gattttgatggattttgcagGAGAATTAGCCACAGAACTCACCATCAAAACCATGGACAC 1916 H-box2
RG10               gattttgatggattttgcagGAGAATTAGCCACAGAACTCACCATCAAAACCATGGACAC 1913
                   ************************************************************
                                                                                 INTRON 3
Glyma.02g227200    ATTGAGAAGGATGAGTCATGGGTTCCAgtatgtgattaattgcttctcctatagttgttc 2038
OX948              ATTGAGAAGGATGAGTCATGGGTTCCAgtatgtgattaattgcttctcctatagttgttc 1976
RG10               ATTGAGAAGGATGAGTCATGGGTTCCAgtatgtgattaattgcttctcctatagttgttc 1973
                   ************************************************************

Glyma.02g227200    ttgattcaattacattttatttatttggtaggtccaagaaaaaagggaatctttatgctt 2098
OX948              ttgattcaattacattttatttatttggtaggtccaagaaaaaagggaatctttatgctt 2036
RG10               ttgattcaattacattttatttatttggtaggtccaagaaaaaagggaatctttatgctt 2033
                   ************************************************************
                                                                                 EXON 4
Glyma.02g227200    cctgaggctgttcttgaacatggctctttttttatgtgtcattatcttagTTAACAGAGAA 2158
OX948              cctgaggctgttcttgaacatggctctttttttatgtgtcattatcttagTTAACAGAGAA 2096
RG10               cctgaggctgttcttgaacatggctctttttttatgtgtcattatcttagTTAACAGAGAA 2093
                   ************************************************************

Glyma.02g227200    GATTTACAAGAATCTAGACAGCATGACAAGACTCATTAGATTCACTGTGCCATTTCCATT 2218
OX948              GATTTACAAGAATCTAGACAGCATGACAAGACTCATTAGATTCACTGTGCCATTTCCATT 2156
RG10               GATTTACAAGAATCTAGACAGCATGACAAGACTCATTAGATTCACTGTGCCATTTCCATT 2153
                   ************************************************************
```

```
                                                                                   INTRON 4
Glyma.02g227200    GTTTGTGTATCCAATTTATTTGgtgagtgatttttgacttggaagacaacaacacatta 2278
OX948              GTTTGTGTATCCAATTTATTTGgtgagtgatttttgacttggaagacaacaacacatta 2216
RG10               GTTTGTGTATCCAATTTATTTGgtgagtgatttttgacttggaagacaacaacacatta 2213
                   ************************************************************

Glyma.02g227200    ttattataatatggttcaaaacaatgactttttctttatgatgtgaactccatttttag 2338
OX948              ttattataatatggttcaaaacaatgactttttctttatgatgtgaactccatttttag 2276
RG10               ttattataatatggttcaaaacaatgactttttctttatgatgtgaactccatttttag 2273
                   ************************************************************
                                                                                   EXON 5
Glyma.02g227200    TTTTCAAGAAGCCCCGGAAAGGAAGGCTCTCACTTCAATCCCTACAGCAATCTGTTTCCA 2398
OX948              TTTTCAAGAAGCCCCGGAAAGGAAGGCTCTCACTTCAATCCCTACAGCAATCTGTTCCCA 2336
RG10               TTTTCAAGAAGCCCCGGAAAGGAAGGCTCTCACTTCAATCCCTACAGCAATCTGTTCCCA 2333
                   ******************************************************** ***

Glyma.02g227200    CCCAGTGAGAGAAAAGGAATAGCAATATCAACACTGTGTTGGGCTACCATGTTTTCTCTG 2458
OX948              CCCAGTGAGAGAAAAGGAATAGCAATATCAACACTGTGTTGGGCTACCATGTTTTCTCTG 2396
RG10               CCCAGTGAGAGAAAAGGAATAGCAATATCAACACTGTGTTGGGCTACCATGTTTTCTCTG 2393
                   ************************************************************

Glyma.02g227200    CTTATCTATCTCTCATTCATAACTAGTCCACTTCTAGTGCTCAAGCTCTATGGAATTCCA 2518
OX948              CTTATCTATCTCTCATTCATAACTAGTCCACTTCTAGTGCTCAAGCTCTATGGAATTCCA 2456
RG10               CTTATCTATCTCTCATTCATAACTAGTCCACTTCTAGTGCTCAAGCTCTATGGAATTCCA 2453
                   ************************************************************

                       *M Fad3B mutation                                            INTRON 5
Glyma.02g227200    TATTGGgtaactaaattactcctacattgttactttttcctcctttttttt-attatttc 2577
OX948              TATTGGgtaactaaattactcctacattgttactttttcctccttttttttattatttc 2516
RG10               TATTGGataactaaattactcctacattgttactttttcctccttttttttattatttc 2513
                   ****** ******************************************** ********

Glyma.02g227200    aattctccaattggaaatttgaaatagttaccataattatgtaattgtttgatcatgtgc 2637
OX948              aattctccaattggaaatttgaaatagttaccataattatgtaattgtttgatcatgtgc 2576
RG10               aattctccaattggaaatttgaaatagttaccataattatgtaattgtttgatcatgtgc 2573
                   ************************************************************
                                                                                   EXON 6
Glyma.02g227200    agATATTTGTTATGTGGCTGGACTTTGTCACATACTTGCATCACCATGGTCACCACCAGA 2697
OX948              agATATTTGTTATGTGGCTGGACTTTGTCACATACTTGCATCACCATGGTCACCACCAGA 2636
RG10               agATATTTGTTATGTGGCTGGACTTTGTCACATACTTGCATCACCATGGTCACCACCAGA 2633
                   ************************************************************
                                                                                   INTRON 6
Glyma.02g227200    AACTGCCTTGGTACCGCGGCAAGgtaacaaaaataaatagaaaatagtgggtgaacactt 2757
OX948              AACTGCCTTGGTACCGCGGCAAGgtaacaaaaataaatagaaaatagtgggtgaacactt 2696
RG10               AACTGCCTTGGTACCGCGGCAAGgtaacaaaaataaatagaaaatagtgggtgaacactt 2693
                   ************************************************************

Glyma.02g227200    aaatgcgagatagtaatacctaaaaaaagaaaaaaatataggtataataaataatataac 2817
OX948              aaatgcgagatagtaatacctaaaaaaagaaaaaaatataggtataataaataatataac 2756
RG10               aaatgcgagatagtaatacctaaaaaaagaaaaaaatataggtataataaataatataac 2753
                   ************************************************************

Glyma.02g227200    tttcaaaataaaaagaaatcatagagtctagcgtagtgtttggagtgaaatgatgttcac 2877
OX948              tttcaaaataaaaagaaatcatagagtctagcgtagtgtttggagtgaaatgatgttcac 2816
RG10               tttcaaaataaaaagaaatcatagagtctagcatagtgtttggagtgaaatgatgttcac 2813
                   ******************************** ***************************

Glyma.02g227200    ctaccattactcaaagatttgttgtgtcccttagttcattcttattattttacat---- 2933
OX948              ctaccattactcaaagatttgttgtgtcccttagttcattcttattattttacattctt 2876
RG10               ctaccattactcaaagatttgttgtgtcccttagttcattcttattattttacattctt 2873
                   ********************************************************

Glyma.02g227200    -----atcttacttgaaaagacttttaattattcattgagatcttaaagtgactgttaa 2988
OX948              tacacatcttacttgaaaagacttttaattattcattgagatcttaaagtgactgttaa 2936
RG10               tacacatcttacttgaaaagacttttaattattcattgagatcttaaagtgactgttaa 2933
                        *******************************************************
```

```
Glyma.02g227200    attaaaataaaaaacaagtttgttaaaacttcaaataaataagagtgaagggagtgtcat 3048
OX948              attaaaataaaaaacaagtttgttaaaacttcaaataaataagagtgaagggagtgtcat 2996
RG10               attaaaataaaaaacaagtttgttaaaacttcaaataaataagagtgaagggagtgtcat 2993
                   ************************************************************

Glyma.02g227200    ttgtcttctttcttttattgcgttattaatcacgtttctcttctcttttttttttttt-c 3107
OX948              ttgtcttctttcttttattgcgttattaatcacgtttctcttctcttttttttttttt-c 3055
RG10               ttgtcttctttcttttattgcgttattaatcacgtttctcttctctttttttttttttttc 3053
                   ********************************************************** *

Glyma.02g227200    ttctctgctttccacccattatcaagttcatgtgaagcagtggcggatctatgtaaatga 3167
OX948              ttctctgctttccacccattatcaagttcatgtgaagcagtggcggatctatgtaaatga 3115
RG10               ttctctgctttccacccattatcaagttcatgtgaagcagtggcggatctatgtaaatga 3113
                   ************************************************************

Glyma.02g227200    gtggggggcaattgcacccacaagatttatttttttatttgtacaggaataataaaataa 3227
OX948              gtggggggcaatttcacccacaagatttatttttttatttgtacaggaataataaaataa 3175
RG10               gtggggggcaattgcacccacaagatttatttttttatttgtacaggaataataaaataa 3173
                   ************* **********************************************

Glyma.02g227200    aactttgccccccataaaaaataaatatttttttcttaaaataatgcaaaataaatataaga 3287
OX948              aactttgccccccataaaaaataaatatttttttcttaaaataatgcaaaataaatataaga 3235
RG10               aactttgccccccataaaaaataaatatttttttcttaaaataatgcaaaataaatataaga 3233
                   ************************************************************

Glyma.02g227200    aataaaaagagaataaattattattaattttattattttgtacttttttatttagtttttt 3347
OX948              aataaaaagaaaataaattattattaattttattattttgtacttttttatttagtttttt 3295
RG10               aataaaaagaaaataaattattattaattttattattttgtacttttttatttagtttttt 3293
                   ********** *************************************************

Glyma.02g227200    tagcggttagatttttttttcatgacattatgtaatctttt-aaaagcatgtaatatttt 3406
OX948              tagcggttagatttttttttcatgacattatgtaatctttt-aaaagcatgtaatatttt 3354
RG10               tagcggttagatttttttttcatgacattatgtaatcttttt aaaagcatgtaatatttt 3353
                   ***************************************** ******************

Glyma.02g227200    tattttgtgaaaataaatataaatgatcatattagtctcagaatgtataaactaataata 3466
OX948              tattttgtgaaaataaatataaatgatcatattagtctcagaatgtataaactaataata 3414
RG10               tattttgtgaaaataaatataaatgatcatattagtctcagaatgtataaactaataata 3413
                   ************************************************************

Glyma.02g227200    attttatcactaaaagaaattctaatttagtccataaataagtaaaacaagtgacaatta 3526
OX948              attttatcactaaaagaaattctaatttagtccataaataagtaaaacaagtgacaatta 3474
RG10               attttatcactaaaagaaattctaatttagtccataaataagtaaaacaagtgacaatta 3473
                   ************************************************************

Glyma.02g227200    tattttatatttacttaatgtgaaataatacttgaacattataataaaacttaatgacag 3586
OX948              tattttatatttacttaatgtgaaataatacttgaacattataataaaacttaatgacag 3534
RG10               tattttatatttacttaatgtgaaataatacttgaacattataataaaacttaatgacag 3533
                   ************************************************************

Glyma.02g227200    gagatattacatagtgccataaagatattttaaaaaataaaatcattaatacactgtact 3646
OX948              gagatattacatagtgccataaagatattttaaaaaataaaatcattaatacactgtact 3594
RG10               gagatattacatagtgccataaagatattttaaaaaataaaatcattaatacactgtact 3593
                   ************************************************************

Glyma.02g227200    actatataatattcgatatatatttttaacatgattctcaatagaaaaattgtattgatt 3706
OX948              actatataatattcgatatatatttttaacatgattctcaatagaaaaattgtattgatt 3654
RG10               actatataatattcgatatatatttttaacatgattctcaatagaaaaattgtattgatt 3653
                   ************************************************************

Glyma.02g227200    atattttattagacatgaatttacaagccccgtttttcatttatagctcttacctgtgat 3766
OX948              atattttattagacatgaatttagaagccccgtttttcatttatagctcttacctgtgat 3714
RG10               atattttattagacatgaatttagaagccccgtttttcatttatagctcttacctgtgat 3713
                   ********************** *************************************
```

```
Glyma.02g227200     ctattgttttgcttcgctgtttttgttggtcaagggacttagatgtcacaatattaatac 3826
OX948               ctattgttttgcttcgctgtttttgttggtcaagggacttagatgtcacaatattaatac 3774
RG10                ctattgttttgcttcgctgtttttgttggtcaagggacttagatgtcacaatattaatac 3773
                    ************************************************************

Glyma.02g227200     tagaagtaaatatttatgaaaacatgtaccttacctc-aacaaagaaa-gtgtggtaagt 3884
OX948               tagaagtaaatatttatgaaaacatgtaccttacctcgaacaaagaaaagtgtggtaagt 3834
RG10                tagaagtaaatatttatgaaaacatgtaccttacctcgaacaaagaaaagtgtggtaagt 3833
                    ************************************* ********** ***********

Glyma.02g227200     ggcaacacacgtgttgcatttttggcccagcaataacacgtgtttttgtggtgtactaaa 3944
OX948               ggcaacacacgtgttgcatttttggcccagcaataacacgtgtttttgtggtgtactaaa 3894
RG10                ggcaacacacgtgttgcatttttggcccagcaataacacgtgtttttgtggtgtactaaa 3893
                    ************************************************************
                                                                                      EXON 7
Glyma.02g227200     atggacagGAATGGAGTTATTTAAGAGGTGGCCTCACCACTGTGGATCGTGACTATGGTT 4004
OX948               atggacagGAATGGAGTTATTTAAGAGGTGGCCTCACCACTGTGGATCGTGACTATGGTT 3954
RG10                atggacagGAATGGAGTTATTTAAGAGGTGGCCTCACCACTGTGGATCGTGACTATGGTT 3953
                    ************************************************************

                                                     H        H  H
Glyma.02g227200     GGATCAATAACATTCACCATGACATTGGCACCCATGTTATCCACCATCTTTTCCCCCAAA 4064
OX948               GGATCAATAACATTCACCATGACATTGGCACCCATGTTATCCACCATCTTTTCCCCCAAA 4014 H-box3
RG10                GGATCAATAACATTCACCATGACATTGGCACCCATGTTATCCACCATCTTTTCCCCCAAA 4013
                    ************************************************************
                                                                                      INTRON 7
Glyma.02g227200     TTCCTCATTATCACCTCGTTGAAGCGgtacattttattgcttattcacctaaaaacaata 4124
OX948               TTCCTCATTATCACCTCGTTGAAGCGgtacattttattgcttattcacctaaaaacaata 4074
RG10                TTCCTCATTATCACCTCGTTGAAGCGgtacattttattgcttattcacctaaaaacaata 4073
                    ************************************************************

Glyma.02g227200     caattagtacatttgttttatctcttggaagttagtcattttcagttgcatgattctaat 4184
OX948               caattagtacatttgttttatctcttggaagttagtcattttcagttgcatgattctaat 4134
RG10                caattagtacatttgttttatctcttggaagttagtcattttcagttgcatgattctaat 4133
                    ************************************************************

Glyma.02g227200     gctctctccattcttaaatcatgttttcacacccacttcatttaaaataagaacgtgggt 4244
OX948               gctctctccattcttaaatcatgttttcacacccacttcatttaaaataagaacgtgggt 4194
RG10                gctctctccattcttaaatcatgttttcacacccacttcatttaaaataagaacgtgggt 4193
                    ************************************************************

Glyma.02g227200     gttattttaatttctattcactaacatgagaaattaacttatttcaagtaataattttaa 4304
OX948               gttattttaatttctattcactaacatgagaaattaacttatttcaagtaataattttaa 4254
RG10                gttattttaatttctattcactaacatgagaaattaacttatttcaagtaataattttaa 4253
                    ************************************************************

Glyma.02g227200     aatattttatgctattattttattacaaataattatgtatattaagtttattgatttta 4364
OX948               aatagttttatgttattattttattacaaataattgtgtatattaagtttattaatttta 4314
RG10                aatagttttatgttattattttattacaaataattgtgtatattaagtttattaatttta 4313
                    **** ******* ********************** ***************** ******

Glyma.02g227200     taataattatattaaaa-ttatatcgatattaatttttgattcactgatagtgttttata 4423
OX948               taataattatattaaaaattatatcgatattaatttttgattcactgatagtgttttata 4374
RG10                taataattatattaaaaattatatcgatattaatttttgattcactgatagtgttttata 4373
                    ***************** ******************************************

Glyma.02g227200     ttgttagtactgtgcatttattttaaaattggcataaataatatatgtaaccagctcact 4483
OX948               ttgttagtactgtgcatttattttaaaattggcataaataatatatgtaaccagctcact 4434
RG10                ttgttagtactgtgcatttattttaaaattggcataaataatatatgtaaccagctcact 4433
                    ************************************************************

Glyma.02g227200     atactatactgggagcttggtggtgaaaggggttcccaaccctcctttctaggtgtacat 4543
OX948               atactatactgggagcttggtggtgaaaggggttcccaaccctcctttctaggtgtacat 4494
RG10                atactatactgggagcttggtggtgaaaggggttcccaaccctcctttctaggtgtacat 4493
                    ************************************************************
```

```
Glyma.02g227200    gctttgatacttctggtaccttcttatatcaatataaattatattttgctgataaaaaaa 4603
OX948              gctttgatacttctggtaccttcttatatcaatataaattatattttgctgataaaaaaa 4554
RG10               gctttgatacttctggtaccttcttatatcaatataaattatattttgctgataaaaaaa 4553
                   ************************************************************

Glyma.02g227200    catggttaaccattaaattcttttttttaaaaaaaaaaactgtatctaaactttgtattatt 4663
OX948              catggttaaccattaaattcttttttttaaaaaaaaaaactgtatctaaactttgtattatt 4614
RG10               catggttaaccattaaattcttttttttaaaaaaaaaaactgtatctaaactttgtattatt 4613
                   ************************************************************
                                                                                    EXON 8
Glyma.02g227200    aaaaagaagtctgagattaacaataaactaacactcatttgaattcactgcagACACAAG 4723
OX948              aaaaagaagtctgagattaacaataaactaacactcatttgaattcactgcagACACAAG 4674
RG10               aaaaagaagtctgagattaacaataaactaacactcatttgaattcactgcagACACAAG 4673
                   ************************************************************

Glyma.02g227200    CAGCAAAACCAGTTCTTGGAGATTACTACCGTGAGCCAGAAAGATCTGCGCCATTACCAT 4783
OX948              CAGCAAAACCAGTTCTTGGAGATTACTACCGTGAGCCAGAAAGATCTGCGCCATTACCAT 4734
RG10               CAGCAAAACCAGTTCTTGGAGATTACTACCGTGAGCCAGAAAGATCTGCGCCATTACCAT 4733
                   ************************************************************

Glyma.02g227200    TTCATCTAATAAAGTATTTAATTCAGAGTATGAGACAAGACCACTTCGTAAGTGACACTG 4843
OX948              TTCATCTAATAAAGTATTTAATTCAGAGTATGAGACAAGACCACTTCGTAAGTGACACTG 4794
RG10               TTCATCTAATAAAGTATTTAATTCAGAGTATGAGACAAGACCACTTCGTAAGTGACACTG 4793
                   ************************************************************

                                                                  TGA           3’UTR
Glyma.02g227200    GAGATGTTGTTTATTATCAGACTGATTCTCTGCTCCTCCACTCGCAACGAGACTGAGTTT 4903 4899
OX948              GAGATGTTGTTTATTATCAGACTGATTCTCTGCTCCTCCACTCGCAACGAGACTGAGTTT 4854 4850
RG10               GAGATGTTGTTTATTATCAGACTGATTCTCTGCTCCTCCACTCGCAACGAGACTGAGTTT 4853 4849
                   ************************************************************

Glyma.02g227200    CAAACTTTTTGGGTTATTATTTATTGGATTCTAGCTACTCAAATTACTTTTTTTTTAATG 4963
OX948              CAAACTTTTTGGGTTATTATTTATTGGATTCTAGCTACTCAAATTACTTTTTTTTTAATG 4914
RG10               CAAACTTTTTGGGTTATTATTTATTGGATTCTAGCTACTCAAATTACTTTTTTTTTAATG 4913
                   ************************************************************

Glyma.02g227200    TTATGTTTTTTGGAGTTTAACGTTTTCTGAACAACTTGCAAATTACTTGCATAGAGAGAC 5023
OX948              TTATGTTTTTTGGAGTTTAACGTTTTCTGAACAACTTGCAAATTACTTGCATAGAGAGAC 4974
RG10               TTATGTTTTTTGGAGTTTAACGTTTTCTGAACAACTTGCAAATTACTTGCATAGAGAGAC 4973
                   ************************************************************

                                            Poly (A)
Glyma.02g227200    ATGGAATATTTATTTGAAATTAGTAAGGTAGTAATAATAAATTTTGAATTGTCAGTTTCA 5083 5059-5064
OX948              ATGGAATATTTATTTGAAATTAGTAAGGTAGTAATAATAAATTTTGAATTGTCAGTTTCA 5034 5010-5015
RG10               ATGGAATATTTATTTGAAATTAGTAAGGTAGTAATAATAAATTTTGAATTGTCAGTTTCA 5033 5009-5014
                   ************************************************************

Glyma.02g227200    TTTGTGATGTTTTCTACCACTTGCACAGAGCATTCACATGGGCAACATACATTTCTAAAA 5143
OX948              TTTGTGATGTTTTCTACCACTTGCACAGAGCATTCACATGGGCAACATACATTTCTAAAA 5094
RG10               TTTGTGATGTTTTCTACCACTTGCACAGAGCATTCACATGGGCAACATACATTTCTAAAA 5093
                   ************************************************************

Glyma.02g227200    TTAAATTATTGCATAAGGCAGTGTTTCTATTATTTAATTAATCGAAAATTTAACAGCAAT 5203
OX948              TTAAATTATTGCATAAGGCAGTGTTTCTATTATTTAATTAATCGAAAATTTAACAGCAAT 5154
RG10               TTAAATTATTGCATAAGGCAGTGTTTCTATTATTTAATTAATCGAAAATTTAACAGCAAT 5153
                   ************************************************************

Glyma.02g227200    TATGCAAGAAATTTACGTGATTTTGGGAAAGTTTCAACCCAAAGTTTGAACAATAATTTG 5263
OX948              TATGCAAGAAATTTACGTGATTTTGGGAAAGTTTCAACCCAAAGTTTGAACAATAATTTG 5214
RG10               TATGCAAGAAATTTACGTGATTTTGGGAAAGTTTCAACCCAAAGTTTGAACAATAATTTG 5213
                   ************************************************************

Glyma.02g227200    TCTCTTATGATTTATATCCAATTTATGATTTAGAACTAACTCTATAGGTGTAAGTAAGTG 5323
OX948              TCTCTTATGATTTATATCCAATTTATGATTTAGAACTAACTCTATAGGTGTAAGTAAGTG 5274
RG10               TCTCTTATGATTTATATCCAATTTATGATTTAGAACTAACTCTATAGGTGTAAGTAAGTG 5273
                   ************************************************************
```

```
Glyma.02g227200    TGAAACTATCACAACAGATTTCAACACCGCTGAAGATCTGTTGTATCTGTAAGTAAAGTC 5383
OX948              TGAAACTATCACAACAGATTTCAACACCGCTGAAGATCTGTTGTATCTGTAAGTAAAGTC 5334
RG10               TGAAACTATCACAACAGATTTCAACACCGCTGAAGATCTGTTGTATCTGTAAGTAAAGTC 5333
                   ************************************************************

Glyma.02g227200    ACTATAGAAGGAGTGGGTCTAAATTGTAAAATAAAGGCAAAAATAGAAATAAGTCTTGAT 5443
OX948              ACTATAGAAGGAGTGGGTCTAAATTGTAAAATAAAGGCAAAAATAGAAATAAGTCTTGAT 5394
RG10               ACTATAGAAGGAGTGGGTCTAAATTGTAAAATAAAGGCAAAAATAGAAATAAGTCTTGAT 5393
                   ************************************************************

Glyma.02g227200    ACAAAAATATAAGAAATTTATTGTCAATTTTATTTAATATTTTAAAAAATCGTACAATTA 5503
OX948              ACAAAAATATAAGAAATTTATTGTCAATTTTATTTAATATTTTAAAAAATCGTACAATTA 5454
RG10               ACAAAAATATAAGAAATTTATTGTCAATTTTATTTAATATTTTAAAAAATCGTACAATTA 5453
                   ************************************************************

Glyma.02g227200    AATAGCAAAATTAGTTAAAATATAAAAATGTACGGTTGTAAGATCTGTTTAATAATTATT 5563
OX948              AATAGCAAAATTAGTTAAAATATAAAAATGTACGGTTATAAGATCTGTTTAATAATTATT 5514
RG10               AATAGCAAAATTAGTTAAAATATAAAAATGTACGGTTATAAGATCTGTTTAATAATTATT 5513
                   ************************************* **********************

Glyma.02g227200    AAATAATGTACGGATATAATATCTAGTACTTAATAATTATTAAAGTAATGAATTATTAAG 5623
OX948              AAATAATGTACGGATATAATATCTAGTACTTAATAATTATTAAAGTAATGAATTATTAAG 5574
RG10               AAATAATGTACGGATATAATATCTAGTACTTAATAATTATTAAAGTAATGAATTATTAAG 5573
                   ************************************************************

Glyma.02g227200    TGAGATTGTGGTTGGAGATGCGCTTTAAGGTGGAGGTACGACTGTTTGTTGTGATGTAGC 5683
OX948              TGAGATTGTGGTTGGAGATGCGCTTTAAGGTGGAGGTACGACTGTTTGTTGTGATGTAGC 5634
RG10               TGAGATTGTGGTTGGAGATGCGCTTTAAGGTGGAGGTACGACTGTTTGTTGTGATGTAGC 5633
                   ************************************************************

Glyma.02g227200    AGAAAAATTAAGAGCATGGTCACTATTCATGCAAGGAATTGATGTAGGGCCGTTAAATGAA 5743
OX948              AGAAAAATTAAGAGCATGGTCACTATTCATGCAAGGAATTGATGTAGGGCCGTTAAATGAA 5694
RG10               AGAAAAATTAAGAGCATGGTCACTATTCATGCAAGGAATTGATGTAGGGCCGTTAAATGAA 5693
                   ************************************************************

Glyma.02g227200    TAACTAGGGTTCTGAAGAAAGACTCTGAAATGAACACAAGCCAAATTCATTGAGTGAAAG 5803
OX948              TAACTAGGGTTCTGAAGAAAGACTCTGAAATGAACACAAGCCAAATTCATTGAGTGAAAG 5754
RG10               TAACTAGGGTTCTGAAGAAAGACTCTGAAATGAACACAAGCCAAATTCATTGAGTGAAAG 5753
                   ************************************************************

Glyma.02g227200    AACTATTCCAAAAAAACACATTTGTTGGTATGCTCACTCTGAGGTCAATAATGGATAGAA 5863
OX948              AACTATTCCAAAAAAACACATTTGTTGGTATGCTCACTCTGAGGTCAATAATGGATAGAA 5814
RG10               AACTATTCCAAAAAAACACATTTGTTGGTATGCTCACTCTGAGGTCAATAATGGATAGAA 5813
                   ************************************************************

Glyma.02g227200    TAAGTTTTATTTTCTCAGATAATTTGTTTTAGGTGATCTTGTTGTATAATTAAACATTAA 5923
OX948              TAAGTTTTATTTTCTCAGATAATTTGTTTTAGGTGATCTTGTTGTATAATTAAACATTAA 5874
RG10               TAAGTTTTATTTTCTCAGATAATTTGTTTTAGGTGATCTTGTTGTATAATTAAACATTAA 5873
                   ************************************************************

Glyma.02g227200    ATATAGATTGTTTTCTAACAAATATTTCAATCTTAATTCGTCACATAAAAAGATCCTGAG 5983
OX948              ATATAGATTGTTTTCTAACAAATATTTCAATCTTAATTCGTCACATAAAAAGATCCTGAG 5934
RG10               ATATAGATTGTTTTCTAACAAATATTTCAATCTTAATTCGTCACATAAAAAGATCCTGAG 5933
                   ************************************************************

Glyma.02g227200    GGAAGGGATTTGGGCCCTTGCTCTGCTTCAATATGTCCTTGTTCCTATCATTATTGCCTT 6043
OX948              GGAAGGGATTTGGGCCCTTGCTCTGCTTCAATATGTCCTTGTTCCTATCAT--------- 5985
RG10               GGAAGGGATTTGGGCCCTTGCTCTGCTTCAATATGTCCTTGTTCCTATCAT--------- 5984
                   ***************************************************

Glyma.02g227200    TGCTTCCATAGTTCCACCTCTATTGTCTGGTTGTTCCAA 6082
OX948              ---------------------------------------
RG10               ---------------------------------------
```

FIGURE S3 | Comparison of *FAD3C* gene sequences in Williams 82 (Glyma.18g062000, Wm82.a2.v1), RG10 (KU310964) and OX948 (KU310960); exon sequences are shown in capital letters and intron sequences are shown in small letters; sequence differences are highlighted.

```
                                                                             5'UTR
Glyma.18g062000    TGAGTTCCATGTATGTCCATGACAACACAAGCCATTCCTCCACCACCAAGTTTGAATCAA 60
OX948              -----------------CATGACAACACAAGCCATTCCTCCACCACCAAGTTTGAATCAA 43
RG10               -----------------CATGACAACACAAGCCATTCCTCCACCACCAAGTTTGAATCAA 43
                                    *******************************************

Glyma.18g062000    CTTCATATGAGAACACACCATAACCCATACCTTTTAAATGTTGAGCATCATTGTTTCCTC 120
OX948              CTTCATATGAGAACACACCATAACCCATACCTTTTAAATGTTGAGCATCATTGTTTCCTC 103
RG10               CTTCATATGAGAACACACCATAACCCATACCTTTTAAATGTTGAGCATCATTGTTTCCTC 103
                   ************************************************************

Glyma.18g062000    GTGATCCCAACACCTAGATATGTTGTTGTTGGGGTTGGGTTTGGGTTTGGGTTTCTGGAA 180
OX948              GTGATCCCAACACCTAGATATGTTGTTGTTGGGGTTGGGTTTGGGTTTGGGTTTCTGGAA 163
RG10               GTGATCCCAACACCTAGATATGTTGTTGTTGGGGTTGGGTTTGGGTTTGGGTTTCTGGAA 163
                   ************************************************************

Glyma.18g062000    GTTATGGTGGGTGCTGACGGAGGTAGCAGGGGAGAAGGAAAAAACTTTTGTTTACTTTTC 240
OX948              GTTATGGTGGGTGCTGACGGAGGTAGCAGGGGAGAAGGAAAAAACTTTTGTTTACTTTTC 223
RG10               GTTATGGTGGGTGCTGACGGAGGTAGCAGGGGAGAAGGAAAAAACTTTTGTTTACTTTTC 223
                   ************************************************************

Glyma.18g062000    TTGTATTTTTAATTTTAAAATAAAAATAAATATAGTGATGGTTTTTTAATCGTTACTATT 300
OX948              TTGTATTTTTAATTTTAAAATAAAAATAAATATAGTGATGGTTTTTTAATCGTTACTATT 283
RG10               TTGTATTTTTAATTTTAAAATAAAAATAAATATAGTGATGGTTTTTTAATCGTTACTATT 283
                   ************************************************************

Glyma.18g062000    CATAATTTAAACCACCACAAATTATGATTTAATGTTATGAAAATAATTAAATTAATATAT 360
OX948              CATAATTTAAACCACCACAAATTATGATTTAATGTTATGAAAATAATTAAATTAATATAT 343
RG10               CATAATTTAAACCACCACAAATTATGATTTAATGTTATGAAAATAATTAAATTAATATAT 343
                   ************************************************************

Glyma.18g062000    TTTTAAGAATTTAGAAATCAAATAAAATCTTTTAAAAATTTAGAAATCAAATTAAATTTT 420
OX948              TTTTAAGAATTTAGAAATCAAATAAAATCTTTTAAAAATTTAGAAATCAAATTAAATTTT 403
RG10               TTTTAAGAATTTAGAAATCAAATAAAATCTTTTAAAAATTTAGAAATCAAATTAAATTTT 403
                   ************************************************************

Glyma.18g062000    TTAAAATAATTTAAAGACTAAATTAATAATTAAGTATCTAAAAACTTGAATAAGCTGGCT 480
OX948              TTAAAATAATTTAAAGACTAAATTAATAATTAAGTATCTAAAAACTTGAATAAGCTGGCT 463
RG10               TTAAAATAATTTAAAGACTAAATTAATAATTAAGTATCTAAAAACTTGAATAAGCTGGCT 463
                   ************************************************************

Glyma.18g062000    TAGAAGTCAAATATAACTGAATTTGGTATAAAGATACATGTGCTCATTTATTGTGATACT 540
OX948              TAGAAGTCAAATATAACTGAATTTGGTATAAAGATACATGTGCTCATTTATTGTGATACT 523
RG10               TAGAAGTCAAATATAACTGAATTTGGTATAAAGATACATGTGCTCATTTATTGTGATACT 523
                   ************************************************************

Glyma.18g062000    AAAAAAAAAATTACGAAGTTCATTTATTTTACTTGTTAAAAAACATGAAACAGAAGCAGAA 600
OX948              AAAAAAAAAATTACGAAGTTCATTTATTTTACTTGTTAAAAAACATGAAACAGAAGCAGAA 583
RG10               AAAAAAAAAATTACGAAGTTCATTTATTTTACTTGTTAAAAAACATGAAACAGAAGCAGAA 583
                   ************************************************************

Glyma.18g062000    ATGAGGTAAAGTTGCAAAAAAAAAGTCATAAAATAATATGATGTGAGGAAAAAGTAACATG 660
OX948              ATGAGGTAAAGTTGCAAAAAAAAAGTCATAAAATAATATGATGTGAGGAAAAAGTAACATG 643
RG10               ATGAGGTAAAGTTGCAAAAAAAAAGTCATAAAATAATATGATGTGAGGAAAAAGTAACATG 643
                   ************************************************************

Glyma.18g062000    TATGCGTTAGGAAGGAATAATTAATCAGTTAAAGTTCAACAACAAAAAAGAAAAGATAGG 720
OX948              TATGCGTTAGGAAGGAATAATTAATCAGTTAAAGTTCAACAACAAAAAAGAAAAGATAGG 703
RG10               TATGCGTTAGGAAGGAATAATTAATCAGTTAAAGTTCAACAACAAAAAAGAAAAGATAGG 703
                   ************************************************************
```

```
Glyma.18g062000    TATTTCAACAAGAAATTAACCGTACTTGTGAAATGCTAAGTTTGTTCAATATAATAGTAA 780
OX948              TATTTCAACAAGAAATTAACCGTACTTGTGAAATGCTAAGTTTGTTCAATATAATAGTAA 763
RG10               TATTTCAACAAGAAATTAACCGTACTTGTGAAATGCTAAGTTTGTTCAATATAATAGTAA 763
                   ************************************************************

Glyma.18g062000    TACTATGTGGACAGTACTAGCATTCAACTTCAGGGAAACGATACAGGAAGTCGAGCTGTC 840
OX948              TACTATGTGGACAGTACTAGCATTCAACTTCAGGGAAACGATACAGGAAGTCGAGCTGTC 823
RG10               TACTATGTGGACAGTACTAGCATTCAACTTCAGGGAAACGATACAGGAAGTCGAGCTGTC 823
                   ************************************************************

Glyma.18g062000    GTGATATTAGATTGGTCAGTTTCAAAGACAAAAAATGTTTTTTTAGTAAAGAGAAAAAAT 900
OX948              GTGATATTAGATTGGTCAGTTTCAAAGACAAAAAATGTTTTTTTAGTAAAGAGAAAAAAT 883
RG10               GTGATATTAGATTGGTCAGTTTCAAAGACAAAAAATGTTTTTTTAGTAAAGAGAAAAAAT 883
                   ************************************************************

Glyma.18g062000    GTTCGAATCAATATAATTTATTTTCTTATTTTATCATTCAATTAACTATAATTGGATATG 960
OX948              GTTCGAATCAATATAATTTATTTTCTTATTTTATCATTCAATTAACTATAATTGGATATG 943
RG10               GTTCGAATCAATATAATTTATTTTCTTATTTTATCATTCAATTAACTATAATTGGATATG 943
                   ************************************************************

Glyma.18g062000    ACTTGACTAAATCTAGTTTAAAGTTATTTAAATTTGAATAATTTTTTTTTTTAGAATGTA 1020
OX948              ACTTGACTAAATCTAGTTTAAAGTTATTTAAATTTGAATAATTTTTTTTTTTAGAATGTA 1003
RG10               ACTTGACTAAATCTAGTTTAAAGTTATTTAAATTTGAATAATTTTTTTTTTTAGAATGTA 1003
                   ************************************************************

Glyma.18g062000    GTAAATAAATCTTTGAAAATATAATAAATATTATAAAACTAATTTAATAACAAAACTCTT 1080
OX948              GTAAATAAATCTTTGAAAATATAATAAATATTATAAAACTAATTTAATAACAAAACTCTT 1063
RG10               GTAAATAAATCTTTGAAAGTATAATAAATATTATAAAACTAATTTAATAGCAAAACTCTT 1063
                   ****************** ****************************** **********

Glyma.18g062000    ATTCCAGAAGTCATAAAATAATCTGCATTGGTATATATTTATGTTATTCATCAGAATACA 1140
OX948              ATTCCAGAAGTCATAAAATAATCTGCATTGGTATATATTTATGTTATTCATCAGAATACA 1123
RG10               ATTCCAGAAGTCATAAAATAATCTGCATTGGTATATATTTATGTTATTCATCAGAATACA 1123
                   ************************************************************

Glyma.18g062000    AAAATCATAAGTCCAAGTTATCAATTAGTAATAAAAAAAATTCAAGATTTCCAGGTATAG 1200
OX948              AAAATCATAAGTCCAAGTTATCAATTAGTAATAAAAAAAATTCAAGATTTCCAGGTATAG 1183
RG10               AAAATCATAAGTCCAAGTTATCAATTAGTAATAAAAAAAATTCAAGATTTCCAGGTATAG 1183
                   ************************************************************

Glyma.18g062000    AAAATGAACTAATGTAAAAGAACTTAAGGAATTATTTTGTCAAAATTAAAATTCTTTCTT 1260
OX948              AAAATGAACTAATGTAAAAGAACTTAAGGAATTATTTTGTCAAAATTAAAATTCTTTCTT 1243
RG10               AAAATGAACTAATGTAAAAGAACTTAAGGAATTATTTTGTCAAAATTAAAATTCTTTCTT 1243
                   ************************************************************

Glyma.18g062000    CTTTTCTTGATTATAAAAAAACATTCTTCATATTTATTAAAAAAATTCGTTAATTTCATT 1320
OX948              CTTTTCTTGATTATAAAAAAACATTCTTCATATTTATTAAAAAAATTCGTTAATTTCATT 1303
RG10               CTTTTCTTGATTATAAAAAAACATTCTTCATATTTATTAAAAAAATTCGTTAATTTCATT 1303
                   ************************************************************

Glyma.18g062000    AAATTACATAATTTTTAATTGAAAAATAAACATATTCCTAAATTATCTTTTATTTTATTG 1380
OX948              AAATTACATAATTTTTAATTGAAAAATAAACATATTCCTAAATTATCTTTTATTTTATTG 1363
RG10               AAATTACATAATTTTTAATTGAAAAATAAACATATTCCTAAATTATCTTTTATTTTATTG 1363
                   ************************************************************

Glyma.18g062000    AAATTTGATATCAAACATAAAAAAGTCTTTTTAATGAAGAATATTTTAAAAAGAATCTTA 1440
OX948              AAATTTGATATCAAACATAAAAAAGTCTTTTTAATGAAGAATATTTTAAAAAGAATCTTA 1423
RG10               AAATTTGATATCAAACATAAAAAAGTCTTTTTAATGAAGAATATTTTAAAAAGAATCTTA 1423
                   ************************************************************

Glyma.18g062000    TTAAATAAAATATAGATAGTTGAGATTTATTTATATTTGAGATAAAAAAAAATATAAAAA 1500
OX948              TTAAATAAAATATAGATAGTTGAGATTTATTTATATTTGAGATAAAAAAAAATATAAAAA 1483
RG10               TTAAATAAAATATAGATAGTTGAGATTTATTTATATTTGAGATAAAAAAAAATATAAAAA 1483
                   ************************************************************
```

```
Glyma.18g062000    AAACTTTTATTGTTTGTATTTAAGACCAAAAAAAGTATATTTTTGATACTAAAATATATA 1560
OX948              AAACTTTTATTGTTTGTATTTAAGACCAAAAAAAGTATATTTTTGATACTAAAATATATA 1543
RG10               AAACTTTTATTGTTTGTATTTAAGACCAAAAAAAGTATATTTTTGATACTAAAATATATA 1543
                   ************************************************************

Glyma.18g062000    TTATTTTTTTATTTTAGTTCTTATAAAACTTTCATTGTAATTTTAGTCTTTTATTAAAGA 1620
OX948              TTATTTTTTTATTTTAGTTCTTATAAAACTTTCATTGTAATTTTAGTCTTTTATTAAAGA 1603
RG10               TTATTTTTTTATTTTAGTTCTTATAAAACTTTCATTGTAATTTTAGTCTTTTATTAAAGA 1603
                   ************************************************************

                                                                          TATA-box
Glyma.18g062000    CTTTGAGTGTTACTAGATTCGAAATTCTTAAACATGATTTCAAATGTGAATAAAAAAAAT 1680 1680-1685
OX948              CTTTGAGTGTTACTAGATTCGAAATTCTTAAACATGATTTCAAATGTGAATAAAAAAAAT 1663 1663-1668
RG10               CTTTGAGTGTTACTAGATTCGAAATTCTTAAACATGATTTCAAATGTGAATAAAAAAAAT 1663 1663-1668
                   ************************************************************

Glyma.18g062000    ATAAAAAGCGATATTCAACCAAATAAATCTGGTCAGCCACGATATTTTCACAAAATCAGT 1740
OX948              ATAAAAAGCGATATTCAACCAAATAAATCTGGTCAGCCACGATATTTTCACAAAATCAGT 1723
RG10               ATAAAAAGCGATATTCAACCAAATAAATCTGGTCAGCCACGATATTTTCACAAAATCAGT 1723
                   ************************************************************

Glyma.18g062000    GAAATATTTTGCTCATGTAACGTGTTAGTCCTACTCGTCCTGATATAACCAAGGCATTGA 1800
OX948              GAAATATTTTGCTCATGTAACGTGTTAGTCCTACTCGTCCTGATATAACCAAGGCATTGA 1783
RG10               GAAATATTTTGCTCATGTAACGTGTTAGTCCTACTCGTCCTGATATAACCAAGGCATTGA 1783
                   ************************************************************

Glyma.18g062000    GGTGACATGTATGAATGATGTATCTTGTATTTAGGAGTTTTTCATTCTCAAAGTTACGAA 1860
OX948              GGTGACATGTATGAATGATGTATCTTGTATTTAGGAGTTTTTCATTCTCAAAGTTACGAA 1843
RG10               GGTGACATGTATGAATGATGTATCTTGTATTTAGGAGTTTTTCATTCTCAAAGTTACGAA 1843
                   ************************************************************

Glyma.18g062000    AGCAATGCAATACAAGTAAAGGGTATAAAGGTAAATTAATTAATAAAAAGTATTATGCCG 1920
OX948              AGCAATGCAATACAAGTAAAGGGTATAAAGGTAAATTAATTAATAAAAAGTATTATGCCG 1903
RG10               AGCAATGCAATACAAGTAAAGGGTATAAAGGTAAATTAATTAATAAAAAGTATTATGCCG 1903
                   ************************************************************

Glyma.18g062000    TTACTATTAATATGATTACTGTTACTTTATGCTGTTACATAAATGTGACCCCATGTGTCC 1980
OX948              TTACTATTAATATGATTACTGTTACTTTATGCTGTTACATAAATGTGACCCCATGTGTCC 1963
RG10               TTACTATTAATATGATTACTGTTACTTTATGCTGTTACATAAATGTGACCCCATGTGTCC 1963
                   ************************************************************

Glyma.18g062000    TTCTGAGAGAATTCACCACCATCTGCTCTACACTCATCTTATATATAGCTATCACCCCTC 2040
OX948              TTCTGAGAGAATTCACCACCATCTGCTCTACACTCATCTTATATATAGCTATCACCCCTC 2023
RG10               TTCTGAGAGAATTCACCACCATCTGCTCTACACTCATCTTATATATAGCTATCACCCCTC 2023
                   ************************************************************

                                                               ATG                 EXON 1
Glyma.18g062000    TCCAAAGCAGCATTGTAACAGAGAAAGAAACATTTGAGCAAAAATGGTTCAAGCACAGCC 2100 2084
OX948              TCCAAAGCAGCATTGTAACAGAGAAAGAAACATTTGAGCAAAAATGGTTCAAGCACAGCC 2083 2067
RG10               TCCAAAGCAGCATTGTAACAGAGAAAGAAACATTTGAGCAAAAATGGTTCAAGCACAGCC 2083 2067
                   ************************************************************

Glyma.18g062000    TCTACAACATGTTGGTAATGGTGCAGGGAAAGAAGATCAAGCTTATTTTGATCCAAGTGC 2160
OX948              TCTACAACATGTTGGTAATGGTGCAGGGAAAGAAGATCAAGCTTATTTTGATCCAAGTGC 2143
RG10               TCTACAACATGTTGGTAATGGTGCAGGGAAAGAAGATCAAGCTTATTTTGATCCAAGTGC 2143
                   ************************************************************

Glyma.18g062000    TCCACCACCCTTCAAGATTGCAAATATCAGAGCAGCAATTCCAAAACATTGCTGGGAGAA 2220
OX948              TCCACCACCCTTCAAGATTGCAAATATCAGAGCAGCAATTCCAAAACATTGCTGGGAGAA 2203
RG10               TCCACCACCCTTCAAGATTGCAAATATCAGAGCAGCAATTCCAAAACATTGCTGGGAGAA 2203
                   ************************************************************

Glyma.18g062000    GAACACATTGAGATCTCTGAGTTATGTTCTGAGGGATGTGTTGGTAGTGACTGCATTGGT 2280
OX948              GAACACATTGAGATCTCTGAGTTATGTTCTGAGGGATGTGTTGGTAGTGACTGCATTGGT 2263
RG10               GAACACATTGAGATCTCTGAGTTATGTTCTGAGGGATGTGTTGGTAGTGACTGCATTGGT 2263
                   ************************************************************
```

```
Glyma.18g062000    AGCTGCAGCAATCGGCTTCAATAGCTGGTTCTTCTGGCCACTCTATTGGCCTGCACAAGG 2340
OX948              AGCTGCAGCAATCGGCTTCAATAGCTGGTTCTTCTGGCCACTCTATTGGCCTGCACAAGG 2323
RG10               AGCTGCAGCAATCGGCTTCAATAGCTGGTTCTTCTGGCCACTCTATTGGCCTGCACAAGG 2323
                   ************************************************************

                                                H                                 INTRON 1
Glyma.18g062000    CACAATGTTTTGGGCACTTTTTGTTCTTGGACATGATTGgtaactaattattattacaaa 2400
OX948              CACAATGTTTTGGGCACTTTTTGTTCTTGGACATGATTGgtaactaattattattacaaa 2383 H-box1
RG10               CACAATGTTTTGGGCACTTTTTGTTCTTGGACATGATTGgtaactaattattattacaaa 2383
                   ************************************************************

Glyma.18g062000    ttgttatgttatgttatgttatgttgttgtgccttttttctcagtgatgctttagtcattt 2460
OX948              ttgttatgttatgttatgttatgttgttgtgccttttttctcagtgatgctttagtcattt 2443
RG10               ttgttatgttatgttatgttatgttgttgtgccttttttctcagtgatgctttagtcattt 2443
                   ************************************************************

Glyma.18g062000    catttcacttggttatgcatgattgttcgttcatatgttctgtcatggtgagttctaatt 2520
OX948              catttcacttggttatgcatgattgttcgttcatatgttctgtcatggtgagttctaatt 2503
RG10               catttcacttggttatgcatgattgttcgttcatatgttctgtcatggtgagttctaatt 2503
                   ************************************************************

                                         H                                        EXON 2
Glyma.18g062000    tgattgatgcatggaacagTGGTCATGGAAGTTTTTCAAACAGTCCTTTGTTGAACAGCA 2580
OX948              tgattgatgcatggaacagTGGTCATGGAAGTTTTTCAAACAGTCCTTTGTTGAACAGCA 2563 H-box1
RG10               tgattgatgcatggaacagTGGTCATGGAAGTTTTTCAAACAGTCCTTTGTTGAACAGCA 2563
                   ************************************************************
                                                                                  INTRON 2
Glyma.18g062000    TTGTGGGCCACATCTTGCACTCTTCAATTCTTGTACCATACCATGGATGgtcggttcctt 2640
OX948              TTGTGGGCCACATCTTGCACTCTTCAATTCTTGTACCATACCATGGATGgtcggttcctt 2623
RG10               TTGTGGGCCACATCTTGCACTCTTCAATTCTTGTACCATACCATGGATGgtcggttcctt 2623
                   ************************************************************

Glyma.18g062000    ttagcaacttttcatgttcactttgtccttaaattttttttttatgtttgttaaaaaaatct 2700
OX948              ttagcaacttttcatgttcactttgtccttaaattttttttttatgtttgttaaaaaaatct 2683
RG10               ttagcaacttttcatgttcactttgtccttaaattttttttttatgtttgttaaaaaaatct 2683
                   ************************************************************
                                                                                  EXON 3
Glyma.18g062000    ttggtctgatttaacaacctaaccatttttacaactcatggatttttttgcagGAGAATTA 2760
OX948              ttggtctgatttaacaacctaaccatttttacaactcatggatttttttgcagGAGAATTA 2743
RG10               ttggtctgatttaacaacctaaccatttttacaactcatggatttttttgcagGAGAATTA 2743
                   ************************************************************

                     H      H  H                                                  INTRON 3
Glyma.18g062000    GCCACAGGACTCACCATCAGAACCATGGCCATGTTGAGAAGGATGAATCATGGGTTCCGg 2820
OX948              GCCACAGGACTCACCATCAGAACCATGGCCATGTTGAGAAGGATGAATCATGGGTTCCGg 2803 H-box2
RG10               GCCACAGGACTCACCATCAGAACCATGGCCATGTTGAGAAGGATGAATCATGGGTTCCGg 2803
                   ************************************************************

Glyma.18g062000    tattactatgagtttgcttgattaatttccacatttttctttcttcttaattttaatca 2880
OX948              tattactatgagtttgcttgattaatttccacatttttctttcttcttaattttaatca 2863
RG10               tattactatgagtttgcttgattaatttccacatttttctttcttcttaattttaatca 2863
                   ************************************************************

Glyma.18g062000    gtggttagatttggttgtgttccaatagaagaaaagggggtatctagagagatgtgaatt 2940
OX948              gtggttagatttggttgtgttccaatagaagaaaagggggtatctagagagatgtgaatt 2923
RG10               gtggttagatttggttgtgttccaatagaagaaaagggggtatctagagagatgtgaatt 2923
                   ************************************************************
                                                                                  EXON 4
Glyma.18g062000    tcatgaagtggttcatgattatgtgtctttatgcctttatgtcagCTTACAGAGAAAGTT 3000
OX948              tcatgaagtggttcatgattatgtgtctttatgcctttatgtcagCTTACAGAGAAAGTT 2983
RG10               tcatgaagtggttcatgattatgtgtctttatgcctttatgtcagCTTACAGAGAAAGTT 2983
                   ************************************************************
```

```
Glyma.18g062000    TACAAGAATCTAGACAACATGACAAGAATGATGAGATTCACTCTTCCTTTCCCCATCTTT 3060
OX948              TACAAGAATCTAGACAACATGACAAGAATGATGAGATTCACTCTTCCTTTCCCCATCTTT 3043
RG10               TACAAGAATCTAGACAACATGACAAGAATGATGAGATTCACTCTTCCTTTCCCCATCTTT 3043
                   ************************************************************

                                                                                 INTRON 4
Glyma.18g062000    GCATACCCCTTTTATTTGgtgagaccctctttttccagaatgacagcattattttactat 3120
OX948              GCATACCCCTTTTATTTGgtgagaccctctttttccagaatgacagcattattttactat 3103
RG10               GCATACCCCTTTTATTTGGTGAGACCCTCTTTTTCCAGAATGACAGCATTATTTTACTAT 3103
                   ************************************************************

Glyma.18g062000    atagtacctcaatttttatatttctaaaattttgaattcttgaaattgaaaggaaaggac 3180
OX948              atagtacctcaatttttatatttctaaaattttgaattcttgaaattgaaaggaaaggac 3163
RG10               atagtacctcaatttttatatttctaaaattttgaattcttgaaattgaaaggaaaggac 3163
                   ************************************************************
                                                                                 EXON 5
Glyma.18g062000    tttattgggtctagcatctcactctctctttgtgatatgaaccatatatttcagTGGAGC 3240
OX948              tttattgggtctagcatctcactctctctttgtgatatgaaccatatatttcagTGGAGC 3223
RG10               tttattgggtctagcatctcactctctctttgtgatatgaaccatatatttcagTGGAGC 3223
                   ************************************************************

Glyma.18g062000    AGAAGCCCTGGAAAAGAAGGCTCTCATTTCAACCCTTACAGCAACTTGTTCTCTCCTGGT 3300
OX948              AGAAGCCCTGGAAAAGAAGGCTCTCATTTCAACCCTTACAGCAACTTGTTCTCTCCTGGT 3283
RG10               AGAAGCCCTGGAAAAGAAGGCTCTCATTTCAACCCTTACAGCAACTTGTTCTCTCCTGGT 3283
                   ************************************************************

Glyma.18g062000    GAGAGAAGAGATGTGCTAACTTCAACTCTGTGTTGGGGCATCATGCTTTCTGTGCTTCTC 3360
OX948              GAGAGAAGAGATGTGCTAACTTCAACTCTGTGTTGGGGCATCATGCTTTCTGTGCTTCTC 3343
RG10               GAGAGAAGAGATGTGCTAACTTCAACTCTGTGTTGGGGCATCATGCTTTCTGTGCTTCTC 3343
                   ************************************************************

Glyma.18g062000    TATCTTTCCCTCACAATGGGTCCACTTTTTATGCTCAAGCTCTATGGGGTTCCCTATTTG 3420
OX948              TATCTTTCCCTCACAATGGGTCCACTTTTTATGCTCAAGCTCTATGGGGTTCCCTATTTG 3403
RG10               TATCTTTCCCTCACAATGGGTCCACTTTTTATGCTCAAGCTCTATGGGGTTCCCTATTTG 3403
                   ************************************************************
                                                                                 INTRON 5
Glyma.18g062000    gtaatctcactctcacactttctttatacatcgcacaccagtgtgggttatttgcaacct 3480
OX948              gtaatctcactctcacactttctttatacatcgcacaccagtgtgggttatttgcaacct 3463
RG10               gtaatctcactctcacactttctttatacatcgcacaccagtgtgggttatttgcaacct 3463
                   ************************************************************

Glyma.18g062000    acaccgaagtaatgccctataattaatggggttaacacatgtccaagtccaatattttgt 3540
OX948              acaccgaagtaatgccctataattaatggggttaacacatgtccaagtccaatattttgt 3523
RG10               acaccgaagtaatgccctataattaatggggttaacacatgtccaagtccaatattttgt 3523
                   ************************************************************
                                                                                 EXON 6
Glyma.18g062000    tcacttatttgaacttgaacatgtgtagATCTTCGTCATGTGGCTGGATTTCGTCACGTA 3600
OX948              tcacttatttgaacttgaacatgtgtagATCTTCGTCATGTGGCTGGATTTCGTCACGTA 3583
RG10               tcacttatttgaacttgaacatgtgtagATCTTCGTCATGTGGCTGGATTTCGTCACGTA 3583
                   ************************************************************
                                                                                 INTRON 6
Glyma.18g062000    CTTGCATCATCATGGTTACAAGCAGAAACTGCCTTGGTACCGTGGCCAGgtatcccattt 3660
OX948              CTTGCATCATCATGGTTACAAGCAGAAACTGCCTTGGTACCGTGGCCAGgtatcccattt 3643
RG10               CTTGCATCATCATGGTTACAAGCAGAAACTGCCTTGGTACCGTGGCCAGgtatcccattt 3643
                   ************************************************************

Glyma.18g062000    aacacaatttgtttcattaacattttaagagaattttttttcaaaatagttttcgaaat 3720
OX948              aacacaatttgtttcattaacattttaagagaattttttttcaaaatagttttcgaaat 3703
RG10               aacacaatttgtttcattaacattttaagagaattttttttcaaaatagttttcgaaat 3703
                   ************************************************************

Glyma.18g062000    taagcaaataccaagcaaattgttagatctacgcttgtacttgttttaaagtcaaattca 3780
OX948              taagcaaataccaagcaaattgttagatctacgcttgtacttgttttaaagtcaaattca 3763
RG10               taagcaaataccaagcaaattgttagatctacgcttgtacttgttttaaagtcaaattca 3763
                   ************************************************************
```

```
Glyma.18g062000     tgaccaaattgtcctcacaagtccaaaccgtccactattttattttcacctactttatag 3840
OX948               tgaccaaattgtcctcacaagtccaaaccgtccactattttattttcacctactttatag 3823
RG10                tgaccaaattgtcctcacaagtccaaaccgtccactattttattttcacctactttatag 3823
                    ************************************************************

Glyma.18g062000     cccaatttgtcatttggttacttcagaaaagagaaccccatttgtagtaaatatattatt 3900
OX948               cccaatttgtcatttggttacttcagaaaagagaaccccatttgtagtaaatatattatt 3883
RG10                cccaatttgtcatttggttacttcagaaaagagaaccccatttgtagtaaatatattatt 3883
                    ************************************************************

Glyma.18g062000     tatgaattatggtagtttcaacataaaacatatttatgtgcagttttgccatccttcaaa 3960
OX948               tatgaattatggtagtttcaacataaaacatatttatgtgcagttttgccatccttcaaa 3943
RG10                tatgaattatggtagtttcaacataaaacatatttatgtgcagttttgccatccttcaaa 3943
                    ************************************************************
                                                                                      EXON 7
Glyma.18g062000     agaagatagaaacttactccatgttactctgtctatatgtaatttcacagGAATGGAGTT 4020
OX948               agaagatagaaacttactccatgttactctgtctatatgtaatttcacagGAATGGAGTT 4003
RG10                agaagatagaaacttactccatgttactctgtctatatgtaatttcacagGAATGGAGTT 4003
                    ************************************************************

Glyma.18g062000     ATCTAAGGGGTGGTCTTACAACAGTAGATCGCGACTATGGTTGGATCAACAACATTCACC 4080
OX948               ATCTAAGGGGTGGTCTTACAACAGTAGATCGCGACTATGGTTGGATCAACAACATTCACC 4063
RG10                ATCTAAGGGGTGGTCTTACAACAGTAGATCGCGACTATGGTTGGATCAACAACATTCACC 4063
                    ************************************************************

                                   H        H  H
Glyma.18g062000     ATGACATTGGCACCCATGTTATCCATCACCTTTTCCCTCAAATTCCACATTATCATTTAA 4140
OX948               ATGACATTGGCACCCATGTTATCCATCACCTTTTCCCTCAAATTCCACATTATCATTTAA 4123 H-box3
RG10                ATGACATTGGCACCCATGTTATCCATCACCTTTTCCCTCAAATTCCACATTATCATTTAA 4123
                    ************************************************************
                                                                                      INTRON 7
Glyma.18g062000     TCGAAGCGgtattaattctctatttcacaagaaattattgtatgtctgcctatgtgatct 4200
OX948               TCGAAGCGgtattaattctctatttcacaagaaattattgtatgtctgcctatgtgatct 4183
RG10                TCGAAGCGgtattaattctctatttcacaagaaattattgtatgtctgcctatgtgatct 4183
                    ************************************************************

Glyma.18g062000     aagtcaattttcacataacacatgatcaaactttcttaattctttcttctaaattgaaaa 4260
OX948               aagtcaattttcacataacacatgatcaaactttcttaattctttcttctaaattgaaaa 4243
RG10                aagtcaattttcacataacacatgatcaaactttcttaattctttcttctaaattgaaaa 4243
                    ************************************************************

Glyma.18g062000     agtggattatatgtcaattgaaaattggtcaagaccacaaacatgtgatgatctcccacc 4320
OX948               agtggattatatgtcaattgaaaattggtcaagaccacaaacatgtgatgatctcccacc 4303
RG10                agtggattatatgtcaattgaaaattggtcaagaccacaaacatgtgatgatctcccacc 4303
                    ************************************************************

Glyma.18g062000     ttacatataataatttctcctattctacaatcaataatccttctatggtcctgaattgtt 4380
OX948               ttacatataataatttctcctattctacaatcaataatccttctatggtcctgaattgtt 4363
RG10                ttacatataataatttctcctattctacaatcaataatccttctatggtcctgaattgtt 4363
                    ************************************************************
                                                                                      EXON 8
Glyma.18g062000     cctttctttttttcatttcttattctttttgttgtcccacaatagACTAAAGCAGCAAAG 4440
OX948               cctttctttttttcatttcttattctttttgttgtcccacaatagACTAAAGCAGCAAAG 4423
RG10                cctttctttttttcatttcttattctttttgttgtcccacaatagACTAAAGCAGCAAAG 4423
                    ************************************************************

Glyma.18g062000     GCAGTGCTAGGAAAGTATTATCGTGAGCCTCAGAAATCTGGGCCATTGCCACTTCATCTA 4500
OX948               GCAGTGCTAGGAAAGTATTATCGTGAGCCTCAGAAATCTGGGCCATTGCCACTTCATCTA 4483
RG10                GCAGTGCTAGGAAAGTATTATCGTGAGCCTCAGAAATCTGGGCCATTGCCACTTCATCTA 4483
                    ************************************************************

Glyma.18g062000     ATAAAGTACTTGCTCCACAGCATAAGTCAGGATCACTTCGTTAGCGACTCTGGCGACATT 4560
OX948               ATAAAGTACTTGCTCCACAGCATAAGTCAGGATCACTTCGTTAGCGACTCTGGCGACATT 4543
RG10                ATAAAGTACTTGCTCCACAGCATAAGTCAGGATCACTTCGTTAGCGACTCTGGCGACATT 4543
                    ************************************************************
```

```
                                                                             TAA          3'UTR
Glyma.18g062000    GTGTACTACCAGACTGATTCCCAGCTCCACAAAGATTCTTGGACCCAGTCCAACTAAAGT 4620 4613
OX948              GTGTACTACCAGACTGATTCCCAGCTCCACAAAGATTCTTGGACCCAGTCCAACTAAAGT 4603 4600
RG10               GTGTACTACCAGACTGATTCCCAGCTCCACAAAGATTCTTGGACCCAGTCCAACTAAAGT 4603 4600
                   ************************************************************

Glyma.18g062000    TTTTGATGCTACATTTACCTATTTCACTCTTAAATACTATTTCCTATGTAATATGTAATT 4680
OX948              TTTTGATGCTACATTTACCTATTTCACTCTTAAATACTATTTCCTATGTAATATGTAATT 4663
RG10               TTTTGATGCTACATTTACCTATTTCACTCTTAAATACTATTTCCTATGTAATATGTAATT 4663
                   ************************************************************

Glyma.18g062000    TAGAATATGTTACCTACTCAAATCAATTAGGTGACATGTATAAGCTTTCATAAATTATGC 4740
OX948              TAGAATATGTTACCTACTCAAATCAATTAGGTGACATGTATAAGCTTTCATAAATTATGC 4723
RG10               TAGAATATGTTACCTACTCAAATCAATTAGGTGACATGTATAAGCTTTCATAAATTATGC 4723
                   ************************************************************

Glyma.18g062000    TAGAAATGCACTTACTTTTCAAAGCATGCTATGTTAGAATTGAATCTACTTTTCAATACC 4800
OX948              TAGAAATGCACTTACTTTTCAAAGCATGCTATGTTAGAATTGAATCTACTTTTCAATACC 4783
RG10               TAGAAATGCACTTACTTTTCAAAGCATGCTATGTTAGAATTGAATCTACTTTTCAATACC 4783
                   ************************************************************

Glyma.18g062000    CAGATCATCAATTAGTTGACTTCTATAAGCTTTTATGTGCTTTGTCAGAATTGCACGCGT 4860
OX948              CAGATCATCAATTAGTTGACTTCTATAAGCTTTTATGTGCTTTGTCAGAATTGCACGCGT 4843
RG10               CAGATCATCAATTAGTTGACTTCTATAAGCTTTTATGTGCTTTGTCAGAATTGCACGCGT 4843
                   ************************************************************

Glyma.18g062000    CAAGAATCCTCTTACGATGGCTTAACCAACTATTGAATGAATTTTAAAGAGAAAAGTACA 4920
OX948              CAAGAATCCTCTTACGATGGCTTAACCAACTATTGAATGAATTTTAAAGAGAAAAGTACA 4903
RG10               CAAGAATCCTCTTACGATGGCTTAACCAACTATTGAATGAATTTTAAAGAGAAAAGTACA 4903
                   ************************************************************

                                                                                Poly (A)
Glyma.18g062000    GAATAATAACTATTAGTTGAATAATTAATAGTTAAAATTATGATAATATATATGTATAAT 4980 4978-4983
OX948              GAATAATAACTATTAGTTGAATAATTAATAGTTAAAATTATGATAATATATATGTATAAT 4963 4961-4966
RG10               GAATAATAACTATTAGTTGAATAATTAATAGTTAAAATTATGATAATATATATGTATAAT 4963 4961-4966
                   ************************************************************

Glyma.18g062000    AAATTATAAAATTGAAGTTTCAACCGTTTCAAAGTAACAATTATTGGTGAGATTTTTACT 5040
OX948              AAATTATAAAATTGAAGTTTCAACCGTTTCAAAGTAACAATTATTGGTGAGATTTTTACT 5023
RG10               AAATTATAAAATTGAAGTTTCAACCGTTTCAAAGTAACAATTATTGGTGAGATTTTTACT 5023
                   ************************************************************

Glyma.18g062000    CTTTAAAGTATATCTTGCTCTTTAGACTTTATTAAACTGGAAATCAAATTTTTTTTCTCC 5100
OX948              CTTTAAAGTATATCTTGCTCTTTAGACTTTATTAAACTGGAAATCAAATTTTTTTTCTCC 5083
RG10               CTTTAAAGTATATCTTGCTCTTTAGACTTTATTAAACTGGAAATCAAATTTTTTTTCTCC 5083
                   ************************************************************

Glyma.18g062000    TTATTCGATGGTGTTCGGAATCAGAATGTTAGCATAGTTTAAAAGCAAAATTACAAAGGA 5160
OX948              TTATTCGATGGTGTTCGGAATCAGAATGTTAGCATAGTTTAAAAGCAAAATTACAAAGGA 5143
RG10               TTATTCGATGGTGTTCGGAATCAGAATGTTAGCATAGTTTAAAAGCAAAATTACAAAGGA 5143
                   ************************************************************

Glyma.18g062000    AAGCCATACATAACCCAATCCTTTTTGACATTGTCGTTTTAACAGTACAAAGACAGGTTG 5220
OX948              AAGCCATACATAACCCAATCCTTTTTGACATTGTCGTTTTAACAGTACAAAGACAGGTTG 5203
RG10               AAGCCATACATAACCCAATCCTTTTTGACATTGTCGTTTTAACAGTACAAAGACAGGTTG 5203
                   ************************************************************

Glyma.18g062000    TGAGATGACGTTATAACTAACATAGTAATTCATGTCTGCATCAAGCTCTGCCATGAATCA 5280
OX948              TGAGATGACGTTATAACTAACATAGTAATTCATGTCTGCATCAAGCTCTGCCATGAATCA 5263
RG10               TGAGATGACGTTATAACTAACATAGTAATTCATGTCTGCATCAAGCTCTGCCATGAATCA 5263
                   ************************************************************

Glyma.18g062000    CGTTACAGCAATGTCACTTTTATTTCAACCACACAAAGTGCTCGGTTGGGATACCAAGTG 5340
OX948              CGTTACAGCAATGTCACTTTTATTTCAACCACACAAAGTGCTCGGTTGGGATACCAAGTG 5323
RG10               CGTTACAGCAATGTCACTTTTATTTCAACCACACAAAGTGCTCGGTTGGGATACCAAGTG 5323
                   ************************************************************
```

```
Glyma.18g062000    TATGTTTTGTTATGTTTGATTCAGGAAAGGAATCAAAATGAAAGAAAGAGAGAAAAGAAA 5400
OX948              TATGTTTTGTTATGTTTGATTCAGGAAAGGAATCAAAATGAAAGAAAGAGAGAAAAGAAA 5383
RG10               TATGTTTTGTTATGTTTGATTCAGGAAAGGAATCAAAATGAAAGAAAGAGAGAAAAGAAA 5383
                   ************************************************************

Glyma.18g062000    ATGAGAACAGTATTATAATTTTTAGATTATTTGATAAAAAAAAGTTAAACTTTTTCTTAT 5460
OX948              ATGAGAACAGTATTATAATTTTTAGATTATTTGATAAAAAAAAGTTAAACTTTTTCTTAT 5443
RG10               ATGAGAACAGTATTATAATTTTTAGATTATTTGATAAAAAAAAGTTAAACTTTTTCTTAT 5443
                   ************************************************************

Glyma.18g062000    TTAATTTATAAAAAGTAAAAATAACGTATATTAAAATAATATAAACATCTCTCATAAAAT 5520
OX948              TTAATTTATAAAAAGTAAAAATAACGTATATTAAAATAATATAAACATCTCTCATAAAAT 5503
RG10               TTAATTTATAAAAAGTAAAAATAACGTATATTAAAATAATATAAACATCTCTCATAAAAT 5503
                   ************************************************************

Glyma.18g062000    AGAAAGATATGCAACAATAAATATTTTTTTGTCTTTTTATTAGATTTTTCTCTTCTTTCC 5580
OX948              AGAAAGATATGCAACAATAAATATTTTTTTGTCTTTTTATTAGATTTTTCTCTTCTTTCC 5563
RG10               AGAAAGATATGCAACAATAAATATTTTTTTGTCTTTTTATTAGATTTTTCTCTTCTTTCC 5563
                   ************************************************************

Glyma.18g062000    ATCCTCTCCTTTTAAAAAGATACCAAAAGGCGGAAGTTCGGATTTTTCTACCTCATTTCC 5640
OX948              ATCCTCTCCTTTTAAAAAGATACCAAAAGGCGGAAGTTCGGATTTTTCTACCTCATTTCC 5623
RG10               ATCCTCTCCTTTTAAAAAGATACCAAAAGGCGGAAGTTCGGATTTTTCTACCTCATTTCC 5623
                   ************************************************************

Glyma.18g062000    ATCCTTTCTCATTCACGGTTAACAACAAAAAAAGCATGCATTTTGTTTAACTGTTTTTGG 5700
OX948              ATCCTTTCTCATTCACGGTTAACAACAAAAAAAGCATGCATTTTGTTTAACTGTTTTTGG 5683
RG10               ATCCTTTCTCATTCACGGTTAACAACAAAAAAAGCATGCATTTTGTTTAACTGTTTTTGG 5683
                   ************************************************************

Glyma.18g062000    TCTTCATATTTTTTAAACCATTTTATTCTTACTCTTAATAATATTAAAGTAGTAATAATT 5760
OX948              TCTTCATATTTTTTAAACCATTTTATTCTTACTCTTAATAATATTAAAGTAGTAATAATT 5743
RG10               TCTTCATATTTTTTAAACCATTTTATTCTTACTCTTAATAATATTAAAGTAGTAATAATT 5743
                   ************************************************************

Glyma.18g062000    AATCTTCTTTAATTACGTAAATTAAAACTGACCCATTAATCAAAATTAGATAATCACCCA 5820
OX948              AATCTTCTTTAATTACGTAAATTAAAACTGACCCATTAATCAAAATTAGATAATCACCCA 5803
RG10               AATCTTCTTTAATTACGTAAATTAAAACTGACCCATTAATCAAAATTAGATAATCACCCA 5803
                   ************************************************************

Glyma.18g062000    CTAAATTATTCTTCACTTTTATTGGAGAATTTTACTTTTAAATTATTTCTTAAATTTTTA 5880
OX948              CTAAATTATTCTTCACTTTTATTGGAGAATTTTACTTTTAAATTATTTCTTAAATTTTTA 5863
RG10               CTAAATTATTCTTCACTTTTATTGGAGAATTTTACTTTTAAATTATTTCTTAAATTTTTA 5863
                   ************************************************************

Glyma.18g062000    TTTTTTTTAATTTACATTAACTTCTCATTACTTTTATATTATTTTTTTTTATTCTATTCT 5940
OX948              TTTTTTTTAATTTACATTAACTTCTCATTACTTTTATATTATTTTTTTTTATTCTATTCT 5923
RG10               TTTTTTTTAATTTACATTAACTTCTCATTACTTTTATATTATTTTTTTTTATTCTATTCT 5923
                   ************************************************************

Glyma.18g062000    TTTTTATTAATTATATTTAACTTCTATTTTTTTATCATTTAAAAAATTTAGAGAGGCAAG 6000
OX948              TTTTTATTAATTATATTTAACTTCTATTTTTTTATCATTTAAAAAATTTAGAGAGGCAAG 5983
RG10               TTTTTATTAATTATATTTAACTTCTATTTTTTTATCATTTAAAAAATTTAGAGAGGCAAG 5983
                   ************************************************************

Glyma.18g062000    AACTTCCCCTATTGTCCATAATGTAAAAACAAATAAAAAAAAATCAGGACCATAAGTAATT 6060
OX948              AACTTCCCCTATTGTCCATAATGTAAAAACAAATAAAAAAAAATCAGGACCATAAGTAATT 6043
RG10               AACTTCCCCTATTGTCCATAATGTAAAAACAAATAAAAAAAAATCAGGACCATAAGTAATT 6043
                   ************************************************************

Glyma.18g062000    CTATTAGTTGTAACAATTATTTTTTTTTGTTTTTCTTGTCATATGTCATATGTTGATCTC 6120
OX948              CTATTAGTTGTAACAATTATTTTTTTTTGTTTTTCTTGTCATATGTCATATGTTGATCTC 6103
RG10               CTATTAGTTGTAACAATTATTTTTTTT-GTTTTTCTTGTCATATGTCATATGTTGATCTC 6102
                   *************************** ********************************
```

```
Glyma.18g062000    TTTTGACTTCTTTAATAATTTAGAATAAATACTTTTCCAAAAAATATATATATTCAACCT 6180
OX948              TTTTGACTTCTTTAATAATTTAGAATAAATACTTTTCCAAAAAATATATATATTCAACCT 6163
RG10               TTTTGACTTCTTTAATAATTTAGAATAAATACTTTTCCAAAAAATATATATATTCAACCT 6162
                   ************************************************************

Glyma.18g062000    AGTTTAGTTCTTAAAGGAAAAATATCAAATAGTCCTTTTAATTATTTCGGTGCTATAAAT 6240
OX948              AGTTTAGTTCTTAAAGGAAAAATATCAAATAGTCCTTTTAATTATTTCGGTGCTATAAAT 6223
RG10               AGTTTAGTTCTTAAAGGAAAAATATCAAATAGTCCTTTTAATTATTTCGGTGCTATAAAT 6222
                   ************************************************************

Glyma.18g062000    TAAAATGATACTTTAAGTTATAGTATCAAAATAATTAAGTTTTTATAGTATTAAATTAAA 6300
OX948              TAAAATGATACTTTAAGTTATAGTATCAAAATAATTAAGTTTTTATAGTATTAAATTAAA 6283
RG10               TAAAATGATACTTTAAGTTATAGTATCAAAATAATTAAGTTTTTATAGTATTAAATTAAA 6282
                   ************************************************************

Glyma.18g062000    ATGTCTTATACTTATAATCTTGAAGGATTGGTTAGCTAATTCTTTTAAAGATTAAAAGTC 6360
OX948              ATGTCTTATACTTATAATCTTGAAGGATTGGTTAGCTAATTCTTTTAAAGATTAAAAGTC 6343
RG10               ATGTCTTATACTTATAATCTTGAAGGATTGGTTAGCTAATTCTTTTAAAGATTAAAAGTC 6342
                   ************************************************************

Glyma.18g062000    CCAATATTTTTTTTCAGGGTTCAGTATGGGTATTCACCCAAAAAATTACCACTATGCTTC 6420
OX948              CCAATATTTTTTTTCAGGGTTCAGTATGGGTATTCACCCAAAAAATTACCACTATGCTTC 6403
RG10               CCAATATTTTTTTTCAGGGTTCAGTATGGGTATTCACCCAAAAAATTACCACTATGCTTC 6402
                   ************************************************************

Glyma.18g062000    CAGTCCTCTCAAGGCTAATTGTTAATTAATGACCTAGGTGTCAGGACCTAACTAATGATA 6480
OX948              CAGTCCTCTCAAGGCTAATTGTTAATTAATGACCTAGGTGTCAGGACCTAACTAATGATA 6463
RG10               CAGTCCTCTCAAGGCTAATTGTTAATTAATGACCTAGGTGTCAGGACCTAACTAATGATA 6462
                   ************************************************************

Glyma.18g062000    TATTCTTGCAACTTTGGATGAAACAAAGATCACAGGTTCAGAACTAATTAAGGATATATG 6540
OX948              TATTCTTGCAACTTTGGATGAAACAAAGATCACAGGTTCAGAACTAATTAAGGATATATG 6523
RG10               TATTCTTGCAACTTTGGATGAAACAAAGATCACAGGTTCAGAACTAATTAAGGATATATG 6522
                   ************************************************************

Glyma.18g062000    CTTTGGTGGGATTCAACACATGTTGTCAGTGTCTCCATTTTTTTAATATTAAAAATAGAA 6600
OX948              CTTTGGTGGGATTCAACACATGTTGTCAGTGTCTCCATTTTTTTAATATTAAAAATAGAA 6583
RG10               CTTTGGTGGGATTCAACACATGTTGTCAGTGTCTCCATTTTTTTAATATTAAAAATAGAA 6582
                   ************************************************************

Glyma.18g062000    AAAATAAATGTTGATATTTTGAATTTCCTGATAATGGTATGGAGTATGGACACAGAAAAA 6660
OX948              AAAATAAATGTTGATATTTTGAATTTCCTGATAATGGTATGGAGTATGGACACAGAAAAA 6643
RG10               AAAATAAATGTTGATATTTTGAATTTCCTGATAATGGTATGGAGTATGGACACAGAAAAA 6642
                   ************************************************************

Glyma.18g062000    GTGTGTGACCACTTACCCATTAATTCTCTCTCGTTGGAGAGAATCCTGATATGTACCCTA 6720
OX948              GTGTGTGACCACTTACCCATTAATTCTCTCTCGTTGGAGAGAATCCTGATATGTACCCTA 6703
RG10               GTGTGTGACCACTTACCCATTAATTCTCTCTCGTTGGAGAGAATCCTGATATGTACCCTA 6702
                   ************************************************************

Glyma.18g062000    CTTTCTTTTTGTAGTTATGTGAAGTTAAAACTACTCTTACACAGAGGGAAGAAGAAAAAT 6780
OX948              CTTTCTTTTTGTAGTTATGTGAAGTTAAAACTACTCTTACACAGAGGGAAGAAGAAGAAT 6763
RG10               CTTTCTTTTTGTAGTTATGTGAAGTTAAAACTACTCTTACACAGAGGGAAGAAGAAAAAT 6762
                   ******************************************************** ***

Glyma.18g062000    AACTAAAATAAAACACTTATGCTAAAGATCATTATTGTGCATATAAGGAATAAATATTGG 6840
OX948              AACTAAAATAAAACACTTATGCTAAAGATCATTATTGTGCATATAAGGAATAAATATTGG 6823
RG10               AACTAAAATAAAACACTTATGCTAAAGATCATTATTGTGCATATAAGGAATAAATATTGG 6822
                   ************************************************************

Glyma.18g062000    AAATTTTTTACCAATATAATGGATTACGAGTCCATTACATGAATGAA 6887
OX948              AAAT------------------------------------------- 6827
RG10               AAAT------------------------------------------- 6826
                   ****
```

Figure S4 | Comparison of *Fad3D* gene sequences in Williams 82 (Glyma.11g174100, Wm82.a2.v1), RG10 (KU310965) and OX948 (KU310961); exon sequences are shown in capital letters and intron sequences are shown in small letters; sequence differences are highlighted.

```
                                                                                    5'UTR
Glyma.11g174100     CATGAAACAGAACCAGAAAATGAGGTAAAGTTGCAACAAAAGTCATAAAAAATATGAGGA 60
OX948               -------------------------------------------------------GAGGA 5
RG10                -------------------------------------------------------GAGGA 5
                                                                           *****

Glyma.11g174100     AAAAGTAACATGTATACGTTACGTCAAAAAAAAATGCATACGTTAGGAAGGAATAATTCA 120
OX948               AAAAGTAACATGTATACGTTACTTCAAAAAAAAATGCATACGTTAGGAAGGAATAATTCA 65
RG10                AAAAGTAACATGTATACGTTACGTCAAAAAAAA-TGCATACGTTAGGAAGGAATAATTCA 64
                    ********************** ********** **************************

Glyma.11g174100     TCAGTTAAAACAACCAAAAAGAAAAGATAGGTATTTCAACCAGAAATTAACCGTACTAGT 180
OX948               TCAGTTAAAACAACCAAAAAGAAAAGATAGGTATTTCAACCAGAAATTAACCGTACTAGT 125
RG10                TCAGTTAAAACAACCAAAAAGAAAAGATAGGTATTTCAACCAGAAATTAACCGTACTAGT 124
                    ************************************************************

Glyma.11g174100     GAAATGCCTAGTTTGTTCAATATAATAGTAATACTATGTGGACATGGGACAGTACTAGCA 240
OX948               GAAATGCCTAGTTTGTTCAATATAATAGTAATACTATGTGGACATGGGACAGTACTAGCA 185
RG10                GAAATGCCTAGTTTGTTCAATATAATAGTAATACTATGTGGACATGGGACAGTACTAGCA 184
                    ************************************************************

Glyma.11g174100     TTCAACTTCAAGGAAAAGATACAGAAAGTCGAGTTGTCATGATTAGATTGGTCAATTTTA 300
OX948               TTCAACTTCAAGGAAAAGATACAGAAAGTCGAGTTGTCATGATTAGATTGGTCAATTTTA 245
RG10                TTCAACTTCAAGGAAAAGATACAGAAAGTCGAGTTGTCATGATTAGATTGGTCAATTTTA 244
                    ************************************************************

Glyma.11g174100     AAGGGAAAAAAATGTTTCAATCAATATAATTTATTTTCTTATTTTATTATTCAATTGATTT 360
OX948               AAGGGAAAAAAATGTTTCAATCAATATAATTTATTTTCTTATTTTATTATTCAATTGATTT 305
RG10                AAGGGAAAAAAATGTTTCAATCAATATAATTTATTTTCTTATTTTATTATTCAATCGATTT 304
                    ****************************************************** *****

Glyma.11g174100     GATTATAAATCCAAATATGACTTGATTAAATCTAGTTTAAAGTGTTTTAAATTTGAATAA 420
OX948               GATTATAAATCCAAATATGACTTGATTAAATCTAGTTTAAAGTGTTTTAAATTTGAATAA 365
RG10                GATTATAAATCCAAATATGACTTGATTAAATCTAGTTTAAAGTGTTTTAAATTTGAATAA 364
                    ************************************************************

Glyma.11g174100     ATTTTTATTTTATTTTTTTAGAATTTAGTAAATAAATCTTTGAAAATATAATAAATATTA 480
OX948               ATTTTTATTTTATTTTTTTAGAATTTAGTAAATAAATCTTTGAAAATATAATAAATATTA 425
RG10                ATTTTTATTTTATTTTTTTAGAATTTAGTAAATAAATCTTTGAAAATATAATAAATATTA 424
                    ************************************************************

Glyma.11g174100     TAAAACTAATTTAATATCAAAACTCTCACTCTAGAAGTCATAAAATAATCTGTGTTGGTA 540
OX948               TAAAACTAATTTAATATCAAAACTCTCACTCTAGAAGTCATAAAATAATCTGTGTTGGTA 485
RG10                TAAAACTAATTTAATATCAAAACTCTCACTCTAGAAGTCATAAAATAATCTGTGTTGGTA 484
                    ************************************************************

Glyma.11g174100     TATAGTTATATTATTCATCAGAATACAAAAATCAGAAGTCCAAGTTATCAATTAGTAATT 600
OX948               TATAGTTATATTATTCATCAGAATACAAAAATCAGAAGTCCAAGTTATCAATTAGTAATT 545
RG10                TATAGTTATATTATTCATCAGAATACAAAAATCAGAAGTCCAAGTTATCAATTAGTAATT 544
                    ************************************************************

Glyma.11g174100     TAAAAAATTCAAAATTCCCCAGGTATAGATCAATGAACTAATGCAAAAGAACTTCATGTA 660
OX948               TAAAAAATTCAAAATTCCCCAGGTATAGATCAATGAACTAATGCAAAAGAACTTCATGTA 605
RG10                TAAAAAATTCAAAATTCCCCAGGTATAGATCAATGAACTAATGCAAAAGAACTTCATGTA 604
                    ************************************************************

Glyma.11g174100     TACTTTTCTCAAAATTTAAATATATTTTTGGTACTGAAATATATATTATTTTTTATTTTA 720
OX948               TACTTTTCTCAAAATTTAAATATATTTTTGGTACTGAAATATATATTATTTTTTATTTTA 665
RG10                TACTTTTCTCGAAATTTAAATATATTTTTGGTACTGAAATATATATTATTTTTTATTTTA 664
                    ********** *************************************************
```

```
Glyma.11g174100    GTTCTTATAAATCTTTCATTGTATTTTTAGTCTCTTATTAAAGATTACTAAAAGTATGTA 780
OX948              GTTCTTATAAATCTTTCATTGTATTTTTAGTCTCTTATTAAAGATTACTAAAAGTATGTA 725
RG10               GTTCTTATAAATCTTTCATTGTATCTTTAGTCCCTTATTAAAGATTACTAAAAGTATGTA 724
                   ************************ ******* ***************************

Glyma.11g174100    GGATATGTTTGATTTCTTTTTCTTGAAAATTGTTTTCATTTTCTAAAAAAAAAAAAGAAA 840
OX948              GGATATGTTTGATTTCTTTTTCTTGAAAATTGTTTTCATTTTCTAAAAAAAAAAAAGAAA 785
RG10               GGATATGTTTGATTTCTTTTTCTTGAAAATTGTTTTCATTTTCTAAAAAAAAAAAAGAAA 784
                   ************************************************************

Glyma.11g174100    AAGAAAACCTGTTTGATAAGTGTAGTTTTTAAAATTGTTTTAAGAAAATAATATATGTAT 900
OX948              AAGAAAACCTGTTTGATAAGTGTAGTTTTTAAAATTGTTTTAAGAAAATAATATATGTAT 845
RG10               AAGAAAACCTGTTTGATAGGTGTAGTTTTTAAAATTGTTTTAAGAAAATAATATATGTAT 844
                   ****************** *****************************************

Glyma.11g174100    ATGATTTTAATATAATTTCTATTTATTTATATATGTTATCTATTTTGAAATAATTATTAC 960
OX948              ATGATTTTAATATAATTTCTATTTATTTATATATGTTATCTATTTTGAAATAATTATTAC 905
RG10               ATGATTTTAATATAATTTCTATTTATTTATATATGTTATCTATTTTGAAATAATTATTAC 904
                   ************************************************************

Glyma.11g174100    TTTAAATACTTTTATTATTTTTAAATATTAATATTAAAAATGATATCATCGAAATAAAAT 1020
OX948              TTTAAATACTTTTATTATTTTTAAATATTAATATTAAAAATGATATCATCGAAATAAAAT 965
RG10               TTTAAATACTTTTATTATTTTTAAATATTAATATTAAAAATGATATCATCGAAATAAAAT 964
                   ************************************************************

Glyma.11g174100    GAGATATTCATGTGATCTTTCTATAAATTTTATTTTATTAGATGAAAAAATATTCTCTAT 1080
OX948              GAGATATTCATGTGATCTTTCTATAAATTTTATTTTATTAGATGAAAAAATATTCTCTAT 1025
RG10               GAGATATTCATGTGATCTTTCTATAAATTTTATTTTATTAGATGAAAAAATATTCTCTAT 1024
                   ************************************************************

Glyma.11g174100    TTTTTTTATCCTACGTATACAGCTAGTTAGTATTAGTCCTTAAATATATATATTTTGTTAAT 1140
OX948              TTTTTTTATCCTACGTATACAGCTAGTTAGTATTAGTCCTTAAATATATATATTTTGTTAAT 1085
RG10               TTTTTTTATCCTACGTATACAGCTAGTTAGTATTAGTCCTTAAATATATATATTTTGTTAAT 1084
                   ************************************************************

Glyma.11g174100    GAAAATAAATTTAATTTTTTATGTGAAATTTTATTTATAAATACATATCAATTTATTTTA 1200
OX948              GAAAATAAATTTAATTTTTTATGTGAAATTTTATTTATAAATACATATCAATTTATTTTA 1145
RG10               GAAAATAAATTTAATTTTTTATGTGAAATTTTATTTATAAATACATATCAATTTATTTTA 1144
                   ************************************************************

Glyma.11g174100    ATTATAATTTTTATAATTCCAAGGCTACTTATCTTTCCAAAAATTAGCATTTAGCCTATT 1260
OX948              ATTATAATTTTTATAATTCCAAGGCTACTTATCTTTCCAAAAATTAGCATTTAGCCTATT 1205
RG10               ATTATAATTTTTATAATTCCAAGGCTACTTATCTTTCCAAAAATTAGCATTTAGCCTATT 1204
                   ************************************************************

Glyma.11g174100    TTATATGTGAGAAATTAAATGTCGAGAATATCTAGCTATCTTCTACAATTTGCCTTAAAC 1320
OX948              TTATATGTGAGAAATTAAATGTCGAGAATATCTAGCTATCTTCTACAATTTGCCTTAAAC 1265
RG10               TTATATGTGAGAAATTAAATGTCGAGAATATCTAGCTATCTTCTACAATTTGCCTTAAAC 1264
                   ************************************************************

Glyma.11g174100    TGCATATCACCGTATTCAAAGAGCCATGAAATAGAAAACTTTTTTTTTTTTTGAGAGGA 1380
OX948              TGCATATCACCGTATTCAAAGAGCCATGAAATAGAAAACTTTTTTTTTTTTTGAGAGGA 1325
RG10               TGCATATCACCGTATTCAAAGAGCCATGAAATAGAAAACTTTTTTTTTTTT-GAGAGGA 1323
                   **************************************************** *******

Glyma.11g174100    TGAAATTAATAGAAAACTTAATAATGTAAAAACAAACTAAAAAAATAAATTGGAATAATA 1440
OX948              TGAAATTAATAGAAAACTTAATAATGTAAAAACAAACTAAAAAAATAAATTGGAATAATA 1385
RG10               TGAAATTAATAGAAAACTTAATAATGTAAAAACAAACTAAAAAAATAAATTGGAATAATA 1383
                   ************************************************************

Glyma.11g174100    AATAAAATAAAAATTGAAGTCCTTTTTAAATAGATTTACCATATAATTCAAAAGTAACAA 1500
OX948              AATAAAATAAAAATTGAAGTCCTTTTTAAATAGATTTACCATATAATTCAAAAGTAACAA 1445
RG10               AATAAAATAAAAATTGAAGTCCTTTTTAAATAGATTTACCATATAATTCAAAAGTAACAA 1443
                   ************************************************************
```

```
Glyma.11g174100    ACAAAATACTAATATTACATTAGATTGAAACTTTGATTGATAATTCAGATTTCTTAAATA 1560
OX948              ACAAAATACTAATATTACATTAGNNNNNNNNNNNNNNNNNNNNNNNNNNNNNNNNNNNNN 1505
RG10               ACAAAATACTAATATTACATTAGNNNNNNNNNNNNNNNNNNNNNNNNNNNNNNNNNNNNN 1503
                   ***********************

Glyma.11g174100    AAGTCTCTCACGTGAAAGAAAATATTTCAACCTGATCAATCCGATTTTTTTGGTAAAATT 1620
OX948              NNNNNNNNNNNNNNNNNNNNNNNNNNNNNNNNNNNNNNNNNNNNNNNNNNNNNNNNNNNN 1565
RG10               NNNNNNNNNNNNNNNNNNNNNNNNNNNNNNNNNNNNNNNNNNNNNNNNNNNNNNNNNNNN 1563

Glyma.11g174100    AGTAAATATTTCAAGCATGTAACGTATGAATTAACCCAACTAATTTGATATATTTGAAAG 1680
OX948              NNNNNNNNNNNNNNNNNNNNNNNNNNNNNNNNNNNNNNNNNNNNNNNNNNNNNNNNNNNN 1625
RG10               NNNNNNNNNNNNNNNNNNNNNNNNNNNNNNNNNNNNNNNNNNNNNNNNNNNNNNNNNNNN 1623

Glyma.11g174100    CATCACGACTTAAACAAAGGATATATATATATATATATATATATATATATATCGAAAATT 1740
OX948              NNNNNNNNNNNNNNNNNNNNNNNNNNNNNNNNNNNNNNNNNNNNNNNNNNNNNGAAAATT 1685
RG10               NNNNNNNNNNNNNNNNNNNNNNNNNNNNNNNNNNNNNNNNNNNNNNNNNNNNNGAAAATT 1683
                                                                        *******

Glyma.11g174100    TTAGCCATCAAAAACATTTCTCGTGGTTTTCGTTGTTTCTACAATTAGTATATTAACTTC 1800
OX948              TTAGCCATCAAAAACATTTCTCGTGGTTTTCGTTGTTTCTACAATTAGTATATTAACTTC 1745
RG10               TTAGCCATCAAAAACATTTCTCGTGGTTTTCGTTGTTTCTACAATTAGTATATTAACTTC 1743
                   ************************************************************

Glyma.11g174100    CTTGCTGGTTAGTCCTACTCGTCCTGATATAACCAAGGCATTGAGGTCACATGTATCATG 1860
OX948              CTTGCTGGTTAGTCCTACTCGTCCTGATATAACCAAGGCATTGAGGTCACATGTATCATG 1805
RG10               CTTGCTGGTTAGTCCTACTCGTCCTGATATAACCAAGGCATTGAGGTCACATGTATCATG 1803
                   ************************************************************

Glyma.11g174100    TATCTTTCATTCTCAAAGTCAAGAAAGCAATACAATACAAGTAAAGGGTATAAAAGTGAA 1920
OX948              TATCTTTCATTCTCAAAGTCAAGAAAGCAATACAATACAAGTAAAGGGTATAAAAGTGAA 1865
RG10               TATCTTTCATTCTCAAAGTCAAGAAAGCAATACAATACAAGTAAAGGGTATAAAAGTGAA 1863
                   ************************************************************

Glyma.11g174100    TTAATTAATAAAAAGTATTTTGCTGCCATTATTAATACTATTACTGTTACTTTATGCTGT 1980
OX948              TTAATTAATAAAAAGTATTTTGCTGCCATTATTAATACTATTACTGTTACTTTATGCTGT 1925
RG10               TTAATTAATAAAAAGTATTTTGCTGCCATTATTAATACTATTACTGTTACTTTATGCTGT 1923
                   ************************************************************

Glyma.11g174100    TACATAAATGTGACCCCATGTGTCCTTCTCAGAGAATTCACCACCATCTCGCTCTACACT 2040
OX948              TACATAAATGTGACCCCATGTGTCCTTCTCAGAGAATTCACCCCCATCTCGCTCTACACT 1985
RG10               TACATAAATGTGACCCCATGTGTCCTTCTCAGAGAATTCACCCCCATCTCGCTCTACACT 1983
                   ****************************************** *****************

                                            TATA-box
Glyma.11g174100    CATCTTACAAATATATATATATATATATATATATAGCTATAACCCCTCTCCAAAACAGCATT 2100 2066-2071
OX948              CATCTTACAAATATATATATATATATATATATATAGCTATAACCCCTCTCCAAAACAGCATT 2045 2011-2016
RG10               CATCTTACAAATATATATATATATATATATATATAGCTATAACCCCTCTCCAAAACAGCATT 2043 2009-2014
                   ************************************************************

                                                    ATG                                EXON 1
Glyma.11g174100    GTAACAGAGAAAGAAAGGAACATTTGAGCAAAAATGGTTCAAGCACAGCCTCTACAACAT 2160 2134
OX948              GTAACAGAGAAAGAAAGGAACATTTGAGCAAAAATGGTTCAAGCACAGCCTCTACAACAT 2105 2079
RG10               GTAACAGAGAAAGAAAGGAACATTTGAGCAAAAATGGTTCAAGCACAGCCTCTACAACAT 2103 2077
                   ************************************************************

Glyma.11g174100    GTTGGTAATGGTGCAGGGAAAGAAGATCTAGCTTATAATTTTGATCCAAGTGCTCCACCA 2220
OX948              GTTGGTAATGGTGCAGGGAAAGAAGATCTAGCTTATAATTTTGATCCAAGTGCTCCACCA 2165
RG10               GTTGGTAATGGTGCAGGGAAAGAAGATCTAGCTTATAATTTTGATCCAAGTGCTCCACCA 2163
                   ************************************************************

Glyma.11g174100    CCCTTCAAGATTGCAGATATCAGAGCAGCAATTCCAAAGCATTGCTGGGAGAAGAACACA 2280
OX948              CCCTTCAAGATTGCAGATATCAGAGCAGCAATTCCAAAGCATTGCTGGGAGAAGAACACA 2225
RG10               CCCTTCAAGATTGCAGATATCAGAGCAGCAATTCCAAAGCATTGCTGGGAGAAGAACACA 2223
                   ************************************************************
```

```
Glyma.11g174100    TTGAGATCTCTGAGTTACGTTCTGAGGGATGTGTTGATAGTTTCTGCATTGGTAGCTGCA 2340
OX948              TTGAGATCTCTGAGTTACGTTCTGAGGGATGTGTTGATAGTTTCTGCATTGGTAGCTGCA 2285
RG10               TTGAGATCTCTGAGTTACGTTCTGAGGGATGTGTTGATAGTTTCTGCATTGGTAGCTGCA 2283
                   ************************************************************

Glyma.11g174100    GCAATTGGCTTCAATAGCTGGCTCTTCTGGCCACCCTATTGGTCTGCACAAGGCACAATG 2400
OX948              GCAATTGGCTTCAATAGCTGGCTCTTCTGGCCACCCTATTGGTCTGCACAAGGCACAATG 2345
RG10               GCAATTGGCTTCAATAGCTGGCTCTTCTGGCCACCCTATTGGTCTGCACAAGGCACAATG 2343
                   ************************************************************

                                            H                                     INTRON 1
Glyma.11g174100    TTTTGGGCACTTTTTGTTCTTGGACATGATTGgtaactaatttattattacaaattatgt 2460
OX948              TTTTGGGCACTTTTTGTTCTTGGACATGATTGgtaactaatttattattacaaattatgt 2405 H-box1
RG10               TTTTGGGCACTTTTTGTTCTTGGACATGATTGgtaactaatttattattacaaattatgT 2403
                   ************************************************************

Glyma.11g174100    tatgtgattgtgccttttctcagtaatgcttagtcacttcatttttgtttcgatcaaca 2520
OX948              tatgtgattgtgccttttctcagtaatgcttagtcacttcatttttgtttcgatcaaca 2465
RG10               tatgtgattgtgccttttctcagtaatgcttagtcacttcatttttgtttcgatcaaca 2463
                   ************************************************************

Glyma.11g174100    aatgttagttatcaagatagttactcttttgttagcagagagatttaaacccgcgatctc 2580
OX948              aatgttagttatcaagatagttactcttttgttagcagagagatttaaacccgcgatctc 2525
RG10               aatgttagttatcaagatagttactcttttgttagcagagagatttaaacccgcgatctc 2523
                   ************************************************************

Glyma.11g174100    tttccttctcccttcaccacttcacatttcacttggttattatgcatgattgttcattca 2640
OX948              tttccttctcccttcaccacttcacatttcacttggttattatgcatgattgttcattca 2585
RG10               tttccttctcccttcaccacttcacatttcacttggttattatgcatgattgttcattca 2583
                   ************************************************************

                                                                   H              EXON 2
Glyma.11g174100    tctgttctgtcatggtgaattctaatttgattgatgcattgaacagTGGTCATGGAAGTT 2700
OX948              tctgttctgtcatggtgaattctaatttgattgatgcattgaacagTGGTCATGGAAGTT 2645 H-box1
RG10               tctgttctgtcatggtgaattctaatttgattgatgcattgaacagTGGTCATGGAAGTT 2643
                   ************************************************************

Glyma.11g174100    TTTCCAACAGTCCTATGTTGAACAGCATTGTGGGCCACATCTTGCACTCTTCAATCCTTG 2760
OX948              TTTCCAACAGTCCTTTGTTGAACAGCATTGTGGGCCACATCTTGCACTCTTCAATCCTTG 2705
RG10               TTTCCAACAGTCCTTTGTTGAACAGCATTGTGGGCCACATCTTGCACTCTTCAATCCTTG 2703
                   ************** *********************************************
                                                                                  INTRON 2
Glyma.11g174100    TACCATACCATGGATGgtcgattccttttatcaacttttcatgttcactttgtccttaaa 2820
OX948              TACCATACCATGGATGgtcgattccttttatcaacttttcatgttcactttgtccttaaa 2765
RG10               TACCATACCATGGATGgtcgattccttttatcaacttttcatgttcactttgtccttaaa 2763
                   ************************************************************

Glyma.11g174100    tattttttttgttaaaaaatcttttggtctgatttgtcaacctcaccatttactactc 2880
OX948              tattttttttgttaaaaaatcttttggtctgatttgtcaacctcaccatttactactc 2825
RG10               tattttttttgttaaaaaatcttttggtctgatttgtcaacctcaccatttactactc 2823
                   ************************************************************

                                             H        H  H                        EXON 3
Glyma.11g174100    atggatatcttttgcagGAGAATTAGCCACAGGACTCACCATCAGAACCATGGCCATGTT 2940
OX948              atggatatcttttgcagGAGAATTAGCCACAGGACTCACCATCAGAACCATGGCCATGTT 2885 H-box2
RG10               atggatatcttttgcagGAGAATTAGCCACAGGACTCACCATCAGAACCATGGCCATGTT 2883
                   ************************************************************
                                                                                  INTRON 3
Glyma.11g174100    GAGAAGGATGAATCATGGGTTCCGgtattattatcagtttgcttaattaatttcaacatt 3000
OX948              GAGAAGGATGAATCATGGGTTCCGgtattattatcagtttgcttaattaatttcaacatt 2945
RG10               GAGAAGGATGAATCATGGGTTCCGgtattattatcagtttgcttaattaatttcaacatt 2943
                   ************************************************************
```

```
Glyma.11g174100    tttctttcttctcttaattttaatcagtagttaattagatttgattgtgttccaatagaa 3060
OX948              tttct-----ctcttaattttaatcagtagttaattagatttgattgtgttccaatagaa 3000
RG10               tttct-----ctcttaattttaatcagtagttaattagatttgattgtgttccaatagaa 2998
                   *****     **************************************************

Glyma.11g174100    gaaaaagggtaatctagagatgtgaacttcatgaagtggttcatgattatgtgcctttat 3120
OX948              gaaaaagggtaatctagagatgtgaacttcatgaagtggttcatgattatgtgcctttat 3060
RG10               gaaaaagggtaatctagagatgtgaacttcatgaagtggttcatgattatgtgcctttat 3058
                   ************************************************************
                                                                                     EXON 4
Glyma.11g174100    gactttatgtcagCTTTCAGAGAAAGTTTACAAGAATCTAGACAACATGACAAGAATGAT 3180
OX948              gactttatgtcagCTTTCAGAGAAAGTTTACAAGAATCTAGACAACATGACAAGAATGAT 3120
RG10               gactttatgtcagCTTTCAGAGAAAGTTTACAAGAATCTAGACAACATGACAAGAATGAT 3118
                   ************************************************************
                                                                                     INTRON 4
Glyma.11g174100    GAGATTCACTCTTCCTTTCCCCATCTTTGCATACCCCTTTTATTTGgtgagaccctcttt 3240
OX948              GAGATTCACTCTTCCTTTCCCCATCTTTGCATACCCCTTTTATTTGgtgagaccctcttt 3180
RG10               GAGATTCACTCTTCCTTTCCCCATCTTTGCATACCCCTTTTATTTGgtgagaccctcttt 3178
                   ************************************************************

Glyma.11g174100    ttccagaatgacagcattattttactatatagtacaaaattatacctcaattttattttt 3300
OX948              ttccagaatgacagcattattttactatatagtacaaaattatacctcaattttattttt 3240
RG10               ttccagaatgacagcattattttactatatagtacaaaattatacctcaattttattttt 3238
                   ************************************************************

Glyma.11g174100    atttctaacatttaatatactaatcttattcttaccttaaattttgaaccattcttttga 3360
OX948              atttctaacatttaatatactaatcttattcttaccttaaattttgaaccattcttttga 3300
RG10               atttctaacatttaatatactaatcttattcttaccttaaattttgaaccattcttttga 3298
                   ************************************************************

Glyma.11g174100    attcttgaaattgaaaggactttgttgggtctagctagctaacatctcactctctctctt 3420
OX948              attcttgaaattgaaaggactttgttgggtctagctagctaacatctcactctctctctt 3360
RG10               attcttgaaattgaaaggactttgttgggtctagctagctaacatctcactctctctctt 3358
                   ************************************************************
                                                                                     EXON 5
Glyma.11g174100    ttgtgatatgaaccatatatatcagTGGAGCAGAAGCCCAGGAAAAGAAGGTTCTCATTT 3480
OX948              ttgtgatatgaaccatatatatcagTGGAGCAGAAGCCCAGGAAAAGAAGGTTCTCATTT 3420
RG10               ttgtgatatgaaccatatatatcagTGGAGCAGAAGCCCAGGAAAAGAAGGTTCTCATTT 3418
                   ************************************************************

Glyma.11g174100    CAACCCTTACAGCAACTTGTTCTCCCCTGGTGAGAGAAGAGATGTGATAACTTCAACTCT 3540
OX948              CAACCCTTACAGCAACTTGTTCTCCCCTGGTGAGAGAAGAGATGTGATAACTTCAACTCT 3480
RG10               CAACCCTTACAGCAACTTGTTCTCCCCTGGTGAGAGAAGAGATGTGATAACTTCAACTCT 3478
                   ************************************************************

Glyma.11g174100    TTGTTGGGGCATCATGCTTTCTCTGCTTCTCTATCTTTCCCTCACATTGGATCCACTTTT 3600
OX948              TTGTTGGGGCATCATGCTTTCTCTGCTTCTCTATCTTTCCCTCACATTGGGTCCACTTTT 3540
RG10               TTGTTGGGGCATCATGCTTTCTCTGCTTCTCTATCTTTCCCTCACATTGGGTCCACTTTT 3538
                   ************************************************** *********
                                                                                     INTRON 5
Glyma.11g174100    TATGTTCAAGCTTTATGGGGTTCCTTATTTGgtaatttcactttcatatttttttggttt 3660
OX948              TATGTTCAAGCTTTATGGGGTTCCTTATTTGgtaatttcactttcatatttttttggttt 3600
RG10               TATGTTCAAGCTTTATGGGGTTCCTTATTTGgtaatttcactttcatatttttttggttt 3598
                   ************************************************************

Glyma.11g174100    aaatgtatttttttttctcaatttttttttatctttatccatataactttttttcaatttag 3720
OX948              aaatgtatttttttttctcaatttttttttatctttatccatataactttttttcaatttag 3660
RG10               aaatgtatttttttttctcaatttttttttatctttatccatataactttttttcaatttag 3658
                   ************************************************************

Glyma.11g174100    ttcttataaaatatatttatttatttttcgttcttaaagtattttaaataatatatttt 3780
OX948              ttcttataaaatatatttatttatttttcgttcttaaagtattttaaataatatatttt 3720
RG10               ttcttataaaatatatttatttatttttcgttcttaaagtattttaaataatatatttt 3718
                   ************************************************************
```

```
Glyma.11g174100    taatattcaaatttttttactattcaagaatagtgcattagaaaaaataaaaataaaac 3840
OX948              taatattcaaatttttttactattcaagaatagtgcattagaaaaaataaaaataaaac 3780
RG10               taatattcaaatttttttactattcaagaatagtgcattaaaaaaaataaaaataaaac 3778
                   **************************************** ******************

Glyma.11g174100    aaacatattttataagaattaaaatgaatttttttataaaaataaaaatgaaaaaaaata 3900
OX948              aaacatattttataagaattaaaatgaatttttttataaaaataaaaatgaaaaaaaata 3840
RG10               aaacatattttataagaattaaaatgaatttttttataaaaataaaaatgaaaaaaaata 3838
                   ************************************************************

Glyma.11g174100    ataaattagaaaaattggaatgactaaaaatatattaacacttgctttctttaaacaccac 3960
OX948              ataaattaaaaaaattggaatgactaaaaatatattaacacttgctttctttaaacaccac 3900
RG10               ataaattaaaaaaattggaatgactaaaaatatattaacacttgctttctttaaacaccac 3898
                   ******** ***************************************************
                                                                                 EXON 6
Glyma.11g174100    acaccaatgtgggttaagtaatgccctatcatttattcacttgtttgatcatgtgtagAT 4020
OX948              acaccaatgtgggttaagtaatgccctatcatttattcacttgtttgatcatgtgtagAT 3960
RG10               acaccaatgtgggttaagtaatgccctatcatttattcacttgtttgatcatgtgtagAT 3958
                   ************************************************************

Glyma.11g174100    CTTCGTCGTGTGGCTGGATTTCGTCACATACTTGCATCATCATGGTTACAAGCAGAAACT 4080
OX948              CTTCGTCGTGTGGCTGGATTTCGTCACATACTTGCATCATCATGGTTACAAGCAGAAACT 4020
RG10               CTTCGTCGTGTGGCTGGATTTCGTCACATACTTGCATCATCATGGTTACAAGCAGAAACT 4018
                   ************************************************************
                                                                                 INTRON 6
Glyma.11g174100    ACCTTGGTACCGTGGCCAGgtatcacatttaataaatttgtttcattaactttttaagag 4140
OX948              ACCTTGGTACCGTGGCCAGgtatcacatttaataaatttgtttcattaactttttaagag 4080
RG10               ACCTTGGTACCGTGGCCAGgtatcacatttaataaatttgtttcattaactttttaagag 4078
                   ************************************************************

Glyma.11g174100    aatttaaaaaaaaatagttttcataattaatcaaatatcaagcaagttgttagatctacg 4200
OX948              aatttaaaaaaaaatagttttcataattaatcaaatatcaagcaagttgttagatctacg 4140
RG10               aatttaaaaaaaaatagttttcataattaatcaaatatcaagcaagttgttagatctacg 4138
                   ************************************************************

Glyma.11g174100    cttgcacttgctttaaagtcaaattcagaccatatttaccttccaagtccaaaccgtcaa 4260
OX948              cttgcacttgctttaaagtcaaattcagaccatatttaccttccaagtccaaaccgtcaa 4200
RG10               cttgcacttgctttaaagtcaaattcagaccatatttaccttccaagtccaaaccgtcaa 4198
                   ************************************************************

Glyma.11g174100    ctattttcaccgactttatagtccaatttgtcacatggtcactttaaacagaacccctttt 4320
OX948              ctattttcaccgactttatagtccaatttgtcacatggtcactttaaacagaacccctttt 4260
RG10               ctattttcaccgactttatagtccaatttgtcacatggtcactttaaacagaacccctttt 4258
                   ************************************************************

Glyma.11g174100    tggagtaaacatattatcttaataattgttagtttcaaataaacaacatacttatgtgca 4380
OX948              tggagtaaacatattatcttaataattgttagtttcaaataaacaacatacttatgtgca 4320
RG10               tggagtaaacatattatcttaataattgttagtttcaaataaacaacatacttatgtgca 4318
                   ************************************************************

Glyma.11g174100    aattttgccttccttcaaagaaaagatagaaacttcatgttactctgtctatgtaatttc 4440
OX948              aattttgccttccttcaaagaaaagatagaaacttcatgttactctgtctatgtaatttc 4380
RG10               aattttgccttccttcaaagaaaagatagaaacttcatgttactctgtctatgtaatttc 4378
                   ************************************************************
                                                                                 EXON 7
Glyma.11g174100    acagGAATGGACTTATCTAAGGGGTGGTCTTACAACCGTAGATCGTGACTATGGTTGGAT 4500
OX948              acagGAATGGACTTATCTAAGGGGTGGTCTTACAACCGTAGATCGTGACTATGGTTGGAT 4440
RG10               acagGAATGGACTTATCTAAGGGGTGGTCTTACAACCGTAGATCGTGACTATGGTTGGAT 4438
                   ************************************************************

                                               H         H  H
Glyma.11g174100    CAACAACATTCACCATGACATTGGCACCCATGTCATCCATCACCTTTTCCCTCAGATTCC 4560
OX948              CAACAACATTCACCATGACATTGGCACCCATGTCATCCATCACCTTTTCCCTCAGATTCC 4500 H-box3
RG10               CAACAACATTCACCATGACATTGGCACCCATGTCATCCATCACCTTTTCCCTCAGATTCC 4498
                   ************************************************************
```

```
                                                                                      INTRON 7
Glyma.11g174100    ACATTATCATTTGGTTGAAGCGgtattaattctctgtttcacaaaaaatcattatatgat 4620
OX948              ACATTATCATTTGGTTGAAGCGgtattaattctctgtttcacaaaaaatcattatatgat 4560
RG10               ACATTATCATTTGGTTGAAGCGgtattaattctctgtttcacaaaaaatcattatatgat 4558
                   ************************************************************

Glyma.11g174100    ttaaaacattcgcatatttatttgatgtcggtcaatctccatgtgatacataataaaatt 4680
OX948              ttaaaacattcgcatatttatttgatgtcggtcaatctccatgtgatacataataaaatt 4620
RG10               ttaaaacattcgcatatttatttgatgtcggtcaatctccatgtgatacataataaaatt 4618
                   ************************************************************

Glyma.11g174100    ttagtagtttttttcttttaaactgaaaaggtcaattacatgttaagtgaagattgacca 4740
OX948              ttagtagtttttttcttttaaactgaaaaggtcaattacatgttaagtgaagattgacca 4680
RG10               ttagtagtttttttcttttaaactgaaaaggtcaattacatgttaagtgaagattgacca 4678
                   ************************************************************

Glyma.11g174100    tgatgacaaacatgtcatcatctcctaccatacatataataatttctccattttcacatt 4800
OX948              tgatgacaaacatgtcatcatctcctaccatacatataataatttctccattttcacatt 4740
RG10               tgatgacaaacatgtcatcatctcctaccatacatataataatttctccattttcacatt 4738
                   ************************************************************

Glyma.11g174100    caataatctcaaaaaatgcatacatcaattttcttctatggtcctgaattgttcctttctt 4860
OX948              caataatctcaaaaaatgcatacatcaattttcttctatggtcctgaattgttcctttctt 4800
RG10               caataatctcaaaaaatgcatacatcaattttcttctatggtcctgaattgttcctttctt 4798
                   ************************************************************
                                                                                      EXON 8
Glyma.11g174100    ttttttaatttctctttctttttctttttgttgttccacaatagACTAAAGCAGCTAAG 4920
OX948              ttttttaatttctctttctttttctttttgttgttccacaatagACTAAAGCAGCTAAG 4860
RG10               ttttttaatttctctttctttttctttttgttgttccacaatagACTAAAGCAGCTAAG 4858
                   ************************************************************

Glyma.11g174100    GCAGTGCTAGGAAAGTATTATCGTGAGCCTCAGAAATCAGGGCCATTGCCACTTCATCTT 4980
OX948              GCAGTGCTAGGAAAGTATTATCGTGAGCCTCAGAAATCAGGGCCATTGCCACTTCATCTT 4920
RG10               GCAGTGCTAGGAAAGTATTATCGTGAGCCTCAGAAATCAGGGCCATTGCCACTTCATCTT 4918
                   ************************************************************

Glyma.11g174100    ATCAAGTACTTGCTACACAGCATAAGTCAGGATCACTTCGTTAGTGACTATGGTGACATT 5040
OX948              ATCAAGTACTTGCTACACAGCATAAGTCAGGATCACTTCGTTAGTGACTATGGTGACATT 4980
RG10               ATCAAGTACTTGCTACACAGCATAAGTCAGGATCACTTCGTTAGTGACTATGGTGACATT 4978
                   ************************************************************

                                                                      TAA         3’UTR
Glyma.11g174100    GTGTACTACCAAACTGATTCTCAGTTCCACAAAGATTCTTGGACCAAGTCCAACTAAAGT 5100 5097
OX948              GTGTACTACCAAACTGATTCTCAGTTCCACAAAGATTCTTGGACCAAGTCCAACTAAAGT 5040 5037
RG10               GTGTACTACCAAACTGATTCTCAGTTCCACAAAGATTCTTGGACCAAGTCCAACTAAAGT 5038 5035
                   ************************************************************

Glyma.11g174100    TTTGATGCTACTACATTGACCTATTTTCTCATATGTTACCTAATCAAATCAATTAGGTGA 5160
OX948              TTTGATGCTACTACATTGACCTATTTTCTCATATGTTACCTAATCAAATCAATTAGGTGA 5100
RG10               TTTGATGCTACTACATTGACCTATTTTCTCATATGTTACCTAATCAAATCAATTAGGTGA 5098
                   ************************************************************

Glyma.11g174100    CATGTATAAGCTTTCATAAATTATGCTAGAAATGTACTTACTGTTCAAAGCATGCTATGC 5220
OX948              CATGTATAAGCTTTCATAAATTATGCTAGAAATGTACTTACTGTTCAAAGCATGCTATGC 5160
RG10               CATGTATAAGCTTTCATAAATTATGCTAGAAATGTACTTACTGTTCAAAGCATGCTATGC 5158
                   ************************************************************

Glyma.11g174100    TAGAAAAGAATCTATTTTTCAATACCCAGATCATGAATTAGTTGACTTCTATAAGCTTTT 5280
OX948              TAGAAAAGAATCTATTTTTCAATACCCAGATCATGAATTAGTTGACTTCTATAAGCTTTT 5220
RG10               TAGAAAAGAATCTATTTTTCAATACCCAGATCATGAATTAGTTGACTTCTATAAGCTTTT 5218
                   ************************************************************

Glyma.11g174100    ATATGCTTTGTCAGAATTGCACGCGTCGAAAAACATCTGACGATGGCTGACGATGGCTGA 5340
OX948              ATATGCTTTGTCAGAATTGCACGCGTCGAAAAACATCTGACGATGGCTGACGATGGCTGA 5280
RG10               ATATGCTTTGTCAGAATTGCACGCGTCGAAAAACATCTGACGATGGCTGACGATGGCTGA 5278
                   ************************************************************
```

```
Glyma.11g174100    GCCAACTATTGAATGAATTTTAAAGATAAAAGTACAGAATAATAATTTGTATAATTAATA 5400
OX948              GCCAACTATTGAATGAATTTTAAAGATAAAAGTACAGAATAATAATTTGTATAATTAATA 5340
RG10               GCCAACTATTGAATGAATTTTAAAGATAAAAGTACAGAATAATAATTTGTATAATTAATA 5338
                   ************************************************************

Glyma.11g174100    ATTAAAATTATGATTATATATAAAGGGTCTTGTTAATTGAAGGATACTAAGAGTATGATT 5460
OX948              ATTAAAATTATGATTATATATAAAGGGTCTTGTTAATTGAAGGATACTAAGAGTATGATT 5400
RG10               ATTAAAATTATGATTATATATAAAGGGTCTTGTTAATTGAAGGATACTAAGAGTATGATT 5398
                   ************************************************************

Glyma.11g174100    GATTCGCTAAAAAATAAGGAATTGGGCAGTACAAAAATATTTGTTTAACGTTTGATCTTA 5520
OX948              GATTCGCTAAAAAATAAGGAATTGGGCAGTACAAAAATATTTGTTTAACGTTTGATCTTA 5460
RG10               GATTCGCTAAAAAATAAGGAATTGGGCAGTACAAAAATATTTGTTTAACGTTTGATCTTA 5458
                   ************************************************************

                                        Poly (A)
Glyma.11g174100    AAATGACTGAGGGTCAGTACGAAATAAAGAATGATGGACTAGAAAAATGTCCAAAAACTT 5580 5543-5548
OX948              AAATGACTGAGGGTCAGTACGAAATAAAGAATGATGGACTAGAAAAATGTCCAAAAACTT 5520 5483-5488
RG10               AAATGACTGAGGGTCAGTACGAAATAAAGAATGATGGACTAGAAAAATGTCCAAAAACTT 5518 5481-5486
                   ************************************************************

Glyma.11g174100    GTAACTCAAGTAAATCTCAGTACAATTTTTTGTTTCATGTCTAACAAGAGTAAAAAATAC 5640
OX948              GTAACTCAAGTAAATCTCAGTACAATTTTTTGTTTCATGTCTAACAAGAGTAAAAAATAC 5580
RG10               GTAACTCAAGTAAATCTCAGTACAATTTTTTGTTTCATGTCTAACAAGAGTAAAAAATAC 5578
                   ************************************************************

Glyma.11g174100    AATATTATTCCTATTTTCTGACTTTCATTATCACACACTTTTTTCCTTTTTTCCTATGTG 5700
OX948              AATATTATTCCTATTTTCTGACTTTCATTATCACACACTTTTTTCCTTTTTTCCTATGTG 5640
RG10               AATATTATTCCTATTTTCTGACTTTCATTATCACACACTTTTTTCCTTTTTTCCTATGTG 5638
                   ************************************************************

Glyma.11g174100    TCTCCTTTTCTAAACATCCCTCTCTAGACATTGTACCTTGGCCATCGGCAAGGGTTGCCT 5760
OX948              TCTCCTTTTCTAAACATCCCTCTCTAGACATTGTACCTTGGCCATCGGCAAGGGTTGCCT 5700
RG10               TCTCCTTTTCTAAACATCCCTCTCTAGACATTGTACCTTGGCCATCGGCAAGGGTTGCCT 5698
                   ************************************************************

Glyma.11g174100    TGGCTGTTGGCCTAGGCACCTTGGCTTCCGGCGAGTGTCTTATGAACGGTTGTTGTTTCC 5820
OX948              TGGCTGTTGGCCTAGGCACCTTGGCTTCCGGCGAGTGTCTTATGAACGGTTGTTGTTTCC 5760
RG10               TGGCTGTTGGCCTAGGCACCTTGGCTTCCGGCGAGTGTCTTATGAACGGTTGTTGTTTCC 5758
                   ************************************************************

Glyma.11g174100    TTTTTTGCTTATTATTTCTTTTTTTTATTATTATTAATTTATTCAAATGTTCTCATTGTC 5880
OX948              TTTTTTGCTTATTATTTCTTTTTTTTATTATTATTAATTTATTCAAATGTTCTCATTGTC 5820
RG10               TTTTTTGCTTATTATTTCTTTTTTTTATTATTATTAATTTATTCAAATGTTCTCATTGTC 5818
                   ************************************************************

Glyma.11g174100    ATCTTCTTCTTTCTCTCTATGTTTATTTTCTTCCTTTTGGCCAACTCCAACGAGGCTGCA 5940
OX948              ATCTTCTTCTTTCTCTCTATGTTTATTTTCTTCCTTTTGGCCAACTCCAACGAGGCTGCA 5880
RG10               ATCTTCTTCTTTCTCTCTATGTTTATTTTCTTCCTTTTGGCCAACTCCAACGAGGCTGCA 5878
                   ************************************************************

Glyma.11g174100    TCATGCCGCCATGCTGTCACTGTCATCGTCGTGACCTATGTGGCCTCGCATTGCCGGGCC 6000
OX948              TCATGCCGCCATGCTGTCACTGTCATCGTCGTGACCTATGTGGCCTCGCATTGCCGGGCC 5940
RG10               TCATGCCGCCATGCTGTCACTGTCATCGTCGTGACCTATGTGGCCTCGCATTGCCGGGCC 5938
                   ************************************************************

Glyma.11g174100    TGCGCCCAATGGCATCAAGGGTCGCACCCCCTCCATGAAGGTGTCTCTCCTTTTTGTGTT 6060
OX948              TGCGCCCAATGGCATCAAGGGTCGCACCCCCTCCATGAAGGTGTCTCTCCTTTTTGTGTT 6000
RG10               TGCGCCCAATGGCATCAAGGGTCGCACCCCCTCCATGAAGGTGTCTCTCCTTTTTGTGTT 5998
                   ************************************************************

Glyma.11g174100    GTCAAGGTACTACGAATGCATCTAAGGTTTTATAGTAAAATTCACATTATTTTGTTCGTT 6120
OX948              GTCAAGGTACTACGAATGCATCTAAGGTTTTATAGTAAAATTCACATTATTTTGTTCGTT 6060
RG10               GTCAAGGTACTACGAATGCATCTAAGGTTTTATAGTAAAATTCACATTATTTTGTTCGTT 6058
                   ************************************************************
```

```
Glyma.11g174100    ATATAAACAAATCAAAGAAGATACAATATAAAATTATTTGTAGTGTGCATTGATCACCAA 6180
OX948              ATATAAACAAATCAAAGAAGATACAATATAAAATTATTTGTAGTGTGCATTGATCACCAA 6120
RG10               ATATAAACAAATCAAAGAAGATACAATATAAAATTATTTGTAGTGTGCATTGATCACCAA 6118
                   ************************************************************

Glyma.11g174100    ATAACCTACGATACAAAAAATTATCCTGTGACTCAATTACTTGTGTCTAATAGTGTTCTA 6240
OX948              ------------------------------------------------------------
RG10               ATAACCTACGATACAAAAAATTATCCTGTGACTCAATTACTTGTGTCTAATAGTGTTCTA 6178

Glyma.11g174100    TTTGTTCTATCTCTTTAGAAAATTAATCCGACCCTGATTAAAAATTAAAAGAAAAAATAC 6300
OX948              ------------------------------------------------------------
RG10               TTTGTTCTATCTCTTTAGAAAATTAATCCGACCCTGATTAAAAATTAAAAGAAAAAATAC 6238

Glyma.11g174100    GTATTGTGAAATTATAGGAAAATAAAAAAAAAATCATATACAACACAATTTTTTGCATTT 6360
OX948              ------------------------------------------------------------
RG10               GTATTGTGAAATTATAGGAAAATAAAAAAAAAATCATATACAACACAATTTTTTGCATTT 6298

Glyma.11g174100    GAATAAAAAAAATCTAGTTTAAGTTTGTTAATTAATGATTAGCAGTTGCCATATAGAAAA 6420
OX948              ------------------------------------------------------------
RG10               GAATAAAAAAAATCTAGTTTAAGTTTGTTAATTAATGATTAGCAGTTGCCATATAGAAAA 6358

Glyma.11g174100    GATTAAAATTTCAATCGTTTCAAAGTAATAATTATTAGTGAGAATTTGACTCTTCAACGT 6480
OX948              ------------------------------------------------------------
RG10               GATTAAAATTTCAATCGTTTCAAAGTAATAATTATTAGTGAGAATTTGACTCTTCAACGT 6418

Glyma.11g174100    ATATCTTGCTCTTTAGACTTTATTAAACTGGAAATCAATTTTTTTTCTTTTAATTCGATG 6540
OX948              ------------------------------------------------------------
RG10               ATATCTTGCTCTTTAGACTTTATTAAACTGGAAATCAATTTTTTTTCTTTTAATTCGATG 6478

Glyma.11g174100    GTGTTCATCAGTTCAGAATCAGAATGTTAGCATAGTTTAAAAGCAAAATAACAAAGGAAA 6600
OX948              ------------------------------------------------------------
RG10               GTGTTCATCAGTTCAGAATCAGAATGTTAGCATAGTTTAAAAGCAAAATAACAAAGGAAA 6538

Glyma.11g174100    GCCATACATAACCCAACCCTTTTTGACGTTGTCATTTTAACAGTACAAAGACAGGTTGTG 6660
OX948              ------------------------------------------------------------
RG10               GCCATACATAACCCAACCCTTTTTGACGTTGTCATTTTAACAGTACAAAGACAGGTTGTG 6598

Glyma.11g174100    AGGTGACGTTATAACTAACATAGTACTTCATGTCTGCATCAAGCTCTGCCATGAATCAAT 6720
OX948              ------------------------------------------------------------
RG10               AGGTGACGTTATAACTAACATAGTACTTCATGTCTGCATCAAGCTCTGCCATGAATCAAT 6658

Glyma.11g174100    CACGTTACAATAATGTCACTTTTATTTCAACCACACAAAGGCTCGAAGGTTGGGATACCA 6780
OX948              ------------------------------------------------------------
RG10               CACGTTACAATAATGTCACTTTTATTTCAACCACACAAAGGCTCGAAGGTTGGGATACCA 6718

Glyma.11g174100    AGTGTAAGTTTTGTTATGTTTGATTCAGGAGAAAGTAATCAAAATCAAAGAAAGATAGAA 6840
OX948              ------------------------------------------------------------
RG10               AG---------------------------------------------------------- 6720

Glyma.11g174100    AGGAAAAGGATAACAGAGTATTATAATTTTTAGATTATTTGATGATAAAAAATGTTAATT 6900
OX948              ------------------------------------------------------------
RG10               ------------------------------------------------------------
```

```
Glyma.11g174100    TTTTTTCTCTTATTTAATTGCATAAAAAGTTTTATATATATATATATATATATATATATA 6960
OX948              ------------------------------------------------------------
RG10               ------------------------------------------------------------

Glyma.11g174100    TATATATATATATATATATATATATACACACACAAAGGCTCGAAGGTTGGGATACCAAGT 7020
OX948              ------------------------------------------------------------
RG10               ------------------------------------------------------------

Glyma.11g174100    GTAAGTTTTGTTATGTTTGATTCAGGAGAAAGTAATCAAAATCAAAGAAAGATAGAAAGG 7080
OX948              ------------------------------------------------------------
RG10               ------------------------------------------------------------

Glyma.11g174100    AAAAGGATAACAGAGTATTATAATTTTTAGATTATTTGATGATAAAAAATGTTAATTTTT 7140
OX948              ------------------------------------------------------------
RG10               ------------------------------------------------------------

Glyma.11g174100    TTTCTCTTATTTAATTGCATAAAAAGTTATATATATATATATATATATATATATATATAT 7200
OX948              ------------------------------------------------------------
RG10               ------------------------------------------------------------

Glyma.11g174100    ATATATATATATATATATATATATATATATATTTAATAAAATAGAAAAAAGATGCAACGA 7260
OX948              ------------------------------------------------------------
RG10               ------------------------------------------------------------

Glyma.11g174100    TAAATAAATTTTGTTTCAAGGTCCATGCCAAAAATTTTAGTTTTATCATATGGATTTACA 7320
OX948              ------------------------------------------------------------
RG10               ------------------------------------------------------------

Glyma.11g174100    TTAAAACACTTGTCCCTAGATTTTCAATTATATATCAAACATGCACTAACCA 7372
OX948              ----------------------------------------------------
RG10               ----------------------------------------------------
```

Figure S5 | Comparison of FAD3A, FAD3B, FAD3C and FAD3D in RG10, OX948 and reference Williams 82 protein sequences [Clustal O (1.2.1) and BoxShade]; conserved histidine residues are shown in red, undelined and bold.

```
Glyma.14G194300-FAD3A     1 MVKDTKPLAYAANNGYQKE----AFDPSAPPPFKIAEIRVAIPKHCWVKNPWRSLSYVLR
RG10-FAD3A                1 MVKDTKPLAYAANNGYQKE----AFDPSAPPPFKIAEIRVAIPKHCWVKNPWRSLSYVLR
OX948-FAD3A               1 MVKDTKPLAYAANNGYQKE----AFDPSAPPPFKIAEIRVAIPKHCWVKNPWRSLSYVLR
Glyma.02G227200-FAD3B     1 MVKDTKPLAYAANNGYQQKGSSFDFDPSAPPPFKIAEIRASIPKHCWVKNPWRSLSYVLR
RG10-FAD3B                1 MVKDTKPLAYAANNGYQQKGSSFDFDPSAPPPFKIAEIRASIPKHCWVKNPWRSLSYVLR
OX848-FAD3B               1 MVKDTKPLAYAANNGYQQKGSSFDFDPSAPPPFKIAEIRASIPKHCWVKNPWRSLSYVLR
Glyma.18G062000-FAD3C     1 MV-QAQPLQ-HVGNGAGKEDQAY-FDPSAPPPFKIANIRAAIPKHCWEKNTLRSLSYVLR
RG10-FAD3C                1 MV-QAQPLQ-HVGNGAGKEDQAY-FDPSAPPPFKIANIRAAIPKHCWEKNTLRSLSYVLR
OX948-FAD3C               1 MV-QAQPLQ-HVGNGAGKEDQAY-FDPSAPPPFKIANIRAAIPKHCWEKNTLRSLSYVLR
Glyma.11G174100-FAD3D     1 MV-QAQPLQ-HVGNGAGKEDLAYNFDPSAPPPFKIADIRAAIPKHCWEKNTLRSLSYVLR
RG10-FAD3D                1 MV-QAQPLQ-HVGNGAGKEDLAYNFDPSAPPPFKIADIRAAIPKHCWEKNTLRSLSYVLR
OX948-FAD3D               1 MV-QAQPLQ-HVGNGAGKEDLAYNFDPSAPPPFKIADIRAAIPKHCWEKNTLRSLSYVLR
consensus                 1 **.   ** .   **   ... ..  ************.**..****** **  ********

                                                        FVLGHDCGHGSF    H-box 1
Glyma.14G194300-FAD3A    57 DVLVIAALMAAASHFNNWLLWLIYWPIQGTMFWALFVLGHDCGHGSFSDSPFLNSLVGHI
RG10-FAD3A               57 DVLVIAALMAAASHFNNWLLWLIYWPIQGTMFWALFVLGHDCGHGSFSDSPFLNSLVGHI
OX948-FAD3A              57 DVLVIAALMAAASHFNNWLLWLIYWPIQGTMFWALFVLGHDCGHGSFSDSPFLNSLVGHI
Glyma.02G227200-FAD3B    61 DVLVIAALVAAAIHFDNWLLWLIYCPIQGTMFWALFVLGHDCGHGSFSDSPLLNSLVGHI
RG10-FAD3B               61 DVLVIAALVAAAIHFDNWLLWLIYCPIQGTMFWALFVLGHDCGHGSFSDSPLLNSLVGHI
OX848-FAD3B              61 DVLVIAALVAAAIHFDNWLLWLIYCPIQGTMFWALFVLGHDCGHGSFSDSPLLNSLVGHI
Glyma.18G062000-FAD3C    58 DVLVVTALVAAAIGFNSWFFWPLYWPAQGTMFWALFVLGHDCGHGSFSNSPLLNSIVGHI
RG10-FAD3C               58 DVLVVTALVAAAIGFNSWFFWPLYWPAQGTMFWALFVLGHDCGHGSFSNSPLLNSIVGHI
OX948-FAD3C              58 DVLVVTALVAAAIGFNSWFFWPLYWPAQGTMFWALFVLGHDCGHGSFSNSPLLNSIVGHI
Glyma.11G174100-FAD3D    59 DVLIVSALVAAAIGFNSWLFWPPYWSAQGTMFWALFVLGHDCGHGSFSNSPMLNSIVGHI
RG10-FAD3D               59 DVLIVSALVAAAIGFNSWLFWPPYWSAQGTMFWALFVLGHDCGHGSFSNSPLLNSIVGHI
OX948-FAD3D              59 DVLIVSALVAAAIGFNSWLFWPPYWSAQGTMFWALFVLGHDCGHGSFSNSPLLNSIVGHI
consensus                61 ***...**.***. *. *. * .*..  ********************* **.***.****

                                             H  HH   H-BOX 2
Glyma.14G194300-FAD3A   117 LHSSILVPYHGWRISHRTHHQNHGHIEKDESWVPLTEKIYKNLDNMTRLVRFTVPFPLFV
RG10-FAD3A              117 LHSSILVPYHGWRISHRTHHQNHGHIEKDESWVPLTEKIYKNLDNMTRLVRFTVPFPLFV
OX948-FAD3A             117 LHSSILVPYHGWRISHRTHHQNHGHIEKDESWVPLTEKIYKNLDNMTRLVRFTVPFPLFV
Glyma.02G227200-FAD3B   121 LHSSILVPYHGWRISHRTHHQNHGHIEKDESWVPLTEKIYKNLDSMTRLIRFTVPFPLFV
RG10-FAD3B              121 LHSSILVPYHGWRISHRTHHQNHGHIEKDESWVPLTEKIYKNLDSMTRLIRFTVPFPLFV
OX848-FAD3B             121 LHSSILVPYHGWRISHRTHHQNHGHIEKDESWVPLTEKIYKNLDSMTRLIRFTVPFPLFV
Glyma.18G062000-FAD3C   118 LHSSILVPYHGWRISHRTHHQNHGHVEKDESWVPLTEKVYKNLDNMTRMMRFTLPFPIFA
RG10-FAD3C              118 LHSSILVPYHGWRISHRTHHQNHGHVEKDESWVPLTEKVYKNLDNMTRMMRFTLPFPIFA
OX948-FAD3C             118 LHSSILVPYHGWRISHRTHHQNHGHVEKDESWVPLTEKVYKNLDNMTRMMRFTLPFPIFA
Glyma.11G174100-FAD3D   119 LHSSILVPYHGWRISHRTHHQNHGHVEKDESWVPLSEKVYKNLDNMTRMMRFTLPFPIFA
RG10-FAD3D              119 LHSSILVPYHGWRISHRTHHQNHGHVEKDESWVPLSEKVYKNLDNMTRMMRFTLPFPIFA
OX948-FAD3D             119 LHSSILVPYHGWRISHRTHHQNHGHVEKDESWVPLSEKVYKNLDNMTRMMRFTLPFPIFA
consensus               121 *************************.*********.**.*****.***..***.***.*

Glyma.14G194300-FAD3A   177 YPIYLFSRSPGKEGSHFNPYSNLFPPSERKGIAISTLCWVTMFSMLIYLSFITSPVLLLK
RG10-FAD3A              177 YPIYLFSRSPGKEGSHFNPYSNLFPPSERKGIAISTLCWVTMFSMLIYLSFITSPVLLLK
OX948-FAD3A             177 YPIYLFSRSPGKEGSHFNPYSNLFPPSERKGIAISTLCWVTMFSMLIYLSFITSPVLLLK
Glyma.02G227200-FAD3B   181 YPIYLFSRSPGKEGSHFNPYSNLFPPSERKGIAISTLCWATMFSLLIYLSFITSPLLVLK
RG10-FAD3B              181 YPIYL-------------------------------------------------------
OX848-FAD3B             181 YPIYLFSRSPGKEGSHFNPYSNLFPPSERKGIAISTLCWATMFSLLIYLSFITSPLLVLK
Glyma.18G062000-FAD3C   178 YPFYLWSRSPGKEGSHFNPYSNLFSPGERRDVLTSTLCWGIMLSVLLYLSLTMGPLFMLK
RG10-FAD3C              178 YPFYLWSRSPGKEGSHFNPYSNLFSPGERRDVLTSTLCWGIMLSVLLYLSLTMGPLFMLK
OX948-FAD3C             178 YPFYLWSRSPGKEGSHFNPYSNLFSPGERRDVLTSTLCWGIMLSVLLYLSLTMGPLFMLK
Glyma.11G174100-FAD3D   179 YPFYLWSRSPGKEGSHFNPYSNLFSPGERRDVITSTLCWGIMLSLLLYLSLTLDPLFMFK
RG10-FAD3D              179 YPFYLWSRSPGKEGSHFNPYSNLFSPGERRDVITSTLCWGIMLSLLLYLSLTLGPLFMFK
OX948-FAD3D             179 YPFYLWSRSPGKEGSHFNPYSNLFSPGERRDVITSTLCWGIMLSLLLYLSLTLGPLFMFK
consensus               181 ** **................................................ ......
```

```
Glyma.14G194300-FAD3A  237 LYGIPYWIFVMWLDFVTYLHHHGHHQKLPWYRGKEWSYLRGGLTTVDRDYGWINNIHHDI
RG10-FAD3A             237 LYGIPYWIFVMWLDFVTYLHHHGHHQKLP-------------------------------
OX948-FAD3A            237 LYGIPYWIFVMWLDFVTYLHHHGHHQKLPWYRGKEWSYLRGGLTTVDRDYGWINNIHHDI
Glyma.02G227200-FAD3B  241 LYGIPYWIFVMWLDFVTYLHHHGHHQKLPWYRGKEWSYLRGGLTTVDRDYGWINNIHHDI
RG10-FAD3B                 ------------------------------------------------------------
OX848-FAD3B            241 LYGIPYWIFVMWLDFVTYLHHHGHHQKLPWYRGKEWSYLRGGLTTVDRDYGWINNIHHDI
Glyma.18G062000-FAD3C  238 LYGVPYLIFVMWLDFVTYLHHHGYKQKLPWYRGQEWSYLRGGLTTVDRDYGWINNIHHDI
RG10-FAD3C             238 LYGVPYLIFVMWLDFVTYLHHHGYKQKLPWYRGQEWSYLRGGLTTVDRDYGWINNIHHDI
OX948-FAD3C            238 LYGVPYLIFVMWLDFVTYLHHHGYKQKLPWYRGQEWSYLRGGLTTVDRDYGWINNIHHDI
Glyma.11G174100-FAD3D  239 LYGVPYLIFVVWLDFVTYLHHHGYKQKLPWYRGQEWTYLRGGLTTVDRDYGWINNIHHDI
RG10-FAD3D             239 LYGVPYLIFVVWLDFVTYLHHHGYKQKLPWYRGQEWTYLRGGLTTVDRDYGWINNIHHDI
OX948-FAD3D            239 LYGVPYLIFVVWLDFVTYLHHHGYKQKLPWYRGQEWTYLRGGLTTVDRDYGWINNIHHDI
consensus              241 ............................................................

                             H  HH   H-box 3
Glyma.14G194300-FAD3A  297 GTHVIHHLFPQIPHYHLVEATQAAKSVLGEYYREPERSAPLPFHLIKYLIQSMRQDHFVS
RG10-FAD3A                 ------------------------------------------------------------
OX948-FAD3A            297 GTHVIHHLFPQIPHYHLVEATQAAKSVLGEYYREPERSAPLPFHLIKYLIQSMRQDHFVS
Glyma.02G227200-FAD3B  301 GTHVIHHLFPQIPHYHLVEATQAAKPVLGDYYREPERSAPLPFHLIKYLIQSMRQDHFVS
RG10-FAD3B                 ------------------------------------------------------------
OX848-FAD3B            301 GTHVIHHLFPQIPHYHLVEATQAAKPVLGDYYREPERSAPLPFHLIKYLIQSMRQDHFVS
Glyma.18G062000-FAD3C  298 GTHVIHHLFPQIPHYHLIEATKAAKAVLGKYYREPQKSGPLPLHLIKYLLHSISQDHFVS
RG10-FAD3C             298 GTHVIHHLFPQIPHYHLIEATKAAKAVLGKYYREPQKSGPLPLHLIKYLLHSISQDHFVS
OX948-FAD3C            298 GTHVIHHLFPQIPHYHLIEATKAAKAVLGKYYREPQKSGPLPLHLIKYLLHSISQDHFVS
Glyma.11G174100-FAD3D  299 GTHVIHHLFPQIPHYHLVEATKAAKAVLGKYYREPQKSGPLPLHLIKYLLHSISQDHFVS
RG10-FAD3D             299 GTHVIHHLFPQIPHYHLVEATKAAKAVLGKYYREPQKSGPLPLHLIKYLLHSISQDHFVS
OX948-FAD3D            299 GTHVIHHLFPQIPHYHLVEATKAAKAVLGKYYREPQKSGPLPLHLIKYLLHSISQDHFVS
consensus              301 ............................................................

Glyma.14G194300-FAD3A  357 DTGDVVYYQTDSLHLHSHRD---
RG10-FAD3A                 -----------------------
OX948-FAD3A            357 DTGDVVYYQTDSLHLHSHRD---
Glyma.02G227200-FAD3B  361 DTGDVVYYQTDSLLLHSQRD---
RG10-FAD3B                 -----------------------
OX848-FAD3B            361 DTGDVVYYQTDSLLLHSQRD---
Glyma.18G062000-FAD3C  358 DSGDIVYYQTDSQLHKDSWTQSN
RG10-FAD3C             358 DSGDIVYYQTDSQLHKDSWTQSN
OX948-FAD3C            358 DSGDIVYYQTDSQLHKDSWTQSN
Glyma.11G174100-FAD3D  359 DYGDIVYYQTDSQFHKDSWTKSN
RG10-FAD3D             359 DYGDIVYYQTDSQFHKDSWTKSN
OX948-FAD3D            359 DYGDIVYYQTDSQFHKDSWTKSN
consensus              361 ............. ...... ..
```

Table S1 Soybean ω-3 fatty acid desaturase (*Fad3*) gene-specific sequencing primers

		Primer					PCR product size (bp)		
								Sequenced	
#	Name	Sequence (5' to 3')	Position	Size (bp)	GC (%)	T_M (°C)	Expected	RG10	OX948
Fad3A **– Glyma14g37350 – Gm14:**									
1	Fad3A-5(1kb)	GCTAGGAAGCAATTTGCATGCTAGGAAG<br>ACTGGGATCAAAAGCTTCCTTTTG	2-1,162	28<br>24	46.4<br>41.7	59.8<br>56.0	1,161	1,114	1,112
2	Fad3A-5E3n	CCCACCACGTATCCCTGAA<br>TTCATCCTTCTCAATGTGTCCATGA	1,034-2,019	19<br>25	57.9<br>40.0	56.7<br>56.2	986	986	986
3	Fad3A-E36n	GTGGAACCAAATCTGATTTGAGAAC<br>GGTAGTATCTAACATTTAAGTGTTCACT	1,914-2,981	25<br>28	40.0<br>32.1	54.5<br>52.9	1,068	1,068	1,068
4	Fad3A-E67n	CTTAATCAATATATTTAGAATCTGGTTAACAT<br>GTATTAGTAGAGTGTAATCTATTTCCTCT	2,738-4,344	32<br>29	21.9<br>31.0	51.0<br>51.9	1,607	1,607	1,605
5	Fad3A-3E7n	CTATTATTACTCACCTAAAAAGAATGC<br>TTGAGGCCCACTATGAATTCC	4,113-5,099	27<br>21	29.6<br>47.6	50.5<br>54.7	987	987	987
6	Fad3A-3(1kb)	GTATTACCGTGAGCCAGAAAGATCTGCA<br>AGGGAACAAGCAAGCGTCTTGACTTCC	4,805-6,026	28<br>27	46.4<br>51.9	60.1<br>62.7	1,222	1,011	1,011
Fad3B **– Glyma02g39230 – Gm02:**									
1	FadB-5(1kb)n	GAGTTTTACACTGTATCTATTCATAAAAC<br>AGGATCAAAATCAAAAGAAGAACCCTTTTG	14-1,084	29<br>30	27.6<br>33.3	51.0<br>56.5	1,071	1,023	1,020
2	Fad3B-5E3n	GAAAAGAGAGGAACCACACTCTCT<br>CTCATCCTTCTCAATGTGTCCATGG	951-1,993	24<br>25	45.8<br>48.0	56.2<br>57.4	1,043	1,039	1,045
3	Fad3B-E36n	GGATTTGGAACCAAATTTTATTTGATAAATGAC<br>GTACCAAGGCAGTTTCTGGTGG	1,877-2,711	33<br>22	27.3<br>54.5	54.8<br>58.1	835	836	836
4	Fad3B-E67n	CCACCAGAAACTGCCTTGGTAC<br>TATTGTTTTTAGGTGAATAAGCAATAAAATG	2,690-4,124	22<br>31	54.5<br>22.6	58.1<br>52.2	1,434	1,434	1,432
5	Fad3B-3E7n	CATTTTATTGCTTATTCACCTAAAAACAATA<br>GTTAGTTCTAAATCATAAATTGGATATAAATC	4,094-5,303	31<br>32	22.6<br>21.9	52.2<br>50.3	1,210	1,211	1,211
6	Fad3B-3(1kb)	CCACTCGCAACGAGACTGAGTTTCAAAC<br>GAACAACCAGACAATAGAGGTGGAACTATGG	4,881-6,079	28<br>31	50.0<br>45.2	61.3<br>59.9	1.199	1,154	1,154

[a]Gene:
Fad3A – TA44802_3847 (4, 027 bp); 1 kb flanking - Gm14:46,646,929..46,652,946 (6,027 bp, R)
Fad3B – TA44804_3847; 1 kb flanking - Gm02:44,496,345..44,502,426 (6,082 bp, R)

Fad3A	Glyma14g37350	Gm14:46,647,783..46,651,952	←	recent duplication Gm02 Ks0.1824 (Glyma v1.0)
Fad3B	Glyma02g39230	Gm02:44,497,345..44,501,526	←	recent duplication Gm02 Ks0.1824 (Glyma v1.0)

Table S1 Soybean ω-3 fatty acid desaturase (*Fad3*) gene-specific sequencing primers

	Primer						PCR product size (bp)		
#	Name	Sequence (5' to 3')	Position	Size (bp)	GC (%)	T_M (°C)	Expected	Sequenced RG10	Sequenced OX948
***Fad3C* – Glyma18g06950 – Gm18:**									
1	Fad3C-51(2kb)	CATGACAACACAAGCCATTCCTC<br>CAATGCAGATTATTTTATGACTTCTGG	18-1,110	23<br>27	47.8<br>33.3	56.5<br>52.9	1,093	1,093	1,093
2	Fad3C-52(2kb)n	TGAATAAGCTGGCTTAGAAGTCAA<br>GAATTTCGAATCTAGTAACACTCAAAGTC	467-1,647	24<br>29	37.5<br>34.5	54.2<br>54.2	1,181	1,181	1,181
3	Fad3C-5(1kb)n	GATTTCCAGGTATAGAAAATGAACTAATG<br>CATAACCAAGTGAAATGAAATGACTAAAG	1,186-2,479	29<br>29	31.0<br>31.0	52.6<br>53.4	1,294	1,294	1,294
4	Fad3C-E15n	GTTATGTTATGTTATGTTGTTGTGCC<br>CGGTGTAGGTTGCAAATAACCC	2,408-3,486	26<br>22	34.6<br>50.0	53.2<br>56.3	1,079	1,079	1,079
5	Fad3C-3E5n	CTCACACTTTCTTTATACATCGCAC<br>GTATTTAAGAGTGAAATAGGTAAATGTAGC	3,432-4,657	25<br>30	40.0<br>30.0	54.1<br>52.5	1,226	1,226	1,226
6	Fad3C-3(1kb)	GATTATATGTCAATTGAAAATTGGTCAAGACC<br>GATTATCTAATTTTGATTAATGGGTCAG	4,265-5,815	32<br>28	31.2<br>28.6	55.3<br>50.9	1,550	1,550	1,550
7	Fad3C-31(2kb)n	GGAATCAAAATGAAAGAAAGAGAGAAAAG<br>CTAACCAATCCTTCAAGATTATAAGTATAAGAC	5,369-6,335	29<br>33	31.0<br>30.3	53.5<br>53.8	967	966	967
8	Fad3C-32(2kb)	AGATAATCACCCACTAAATTATTCTTCAC<br>ATTTCCAATATTTATTCCTTATATGCAC	5,808-6,844	29<br>28	31.0<br>25.0	53.4<br>50.6	1,037	1,036	1,037
***Fad3D* – Glyma11g27190 – Gm11:**									
1	Fad3D-51(2kb)n	GAGGAAAAAGTAACATGTATACGTTAC<br>AATACTAACTAGCTGTATACGTAGG	56-1,114	27<br>25	33.3<br>36.0	52.1<br>51.3	1,059	1,058	1,059
2	Fad3D-52(2kb)n	GTGTTGGTATATAGTTATATTATTCATCAG<br>CTAATGTAATATTAGTATTTTGTTTGTTAC	532-1,523	30<br>30	26.7<br>20.0	50.4<br>48.5	992	991	992
3	Fad3D-5(1kb)n	CACCGTATTCAAAGAGCCATG<br>CTAAGCATTACTGAGAAAAGGCAC	1,328-2,493	21<br>24	47.6<br>41.7	53.9<br>54.0	1,166	1,166	1,167
4	Fad3D-E15n	GATATCAGAGCAGCAATTCCAAAG<br>TCACATCTCTTCTCTCACCAGGG	2,236-3,527	24<br>23	41.7<br>52.2	54.1<br>57.8	1,292	1,287	1,287
5	Fad3D-3E5n	GTTGGGTCTAGCTAGCTAAC<br>GAGAAAATAGGTCAATGTAGTAGCATC	3,384-5,130	20<br>27	50.0<br>37.0	52.2<br>53.5	1,747	1,747	1,747
6	Fad3D-3(1kb)	AGGGCCATTGCCACTTCATCTTATC<br>CACTATTAGACACAAGTAATTGAGTCAC	4,959-6,235	25<br>28	48.0<br>35.7	59.2<br>53.7	1,277	1,277	1,277
7	Fad3D-31(2kb)n	CCGGCGAGTGTCTTATGAACG<br>CACTTGGTATCCCAACCTTCGAG	5,788-6,784	21<br>23	57.1<br>52.2	58.0<br>57.4	997	997	396
8	Fad3D-32(2kb)n	GTAGTGTGCATTGATCACCAAATAAC<br>GACAAGTGTTTTAATGTAAATCCATATG	6,160-7,334	26<br>28	38.5<br>28.6	54.7<br>51.5	1,175	641	0

[a]Gene:

Fad3C – TA58104_3847 (2,767 bp); 1 kb flanking - Gm18:6,618,529..5,623,295 (4,767 bp); + 2 kb (upstream and downstream) – 6,887 bp (R) from Gm18:5558601..5879932 (supercontig_271, 321,331 bp)

Fad3D – TA58105_3847 (3,171 bp); 1 kb flanking – Gm11:26,804,260..26,809,430 (5,171 bp); + 2 kb (upstream and downstream) 7,372 bp (R) from Gm11:25147014..27241276 (supercontig_126, 2,094,262 bp), reverse complement

Fad3C	Glyma18:06950	Gm18:5,619,528..5,622,303	→	recent duplication Gm11 Ks0.2225 (Glyma v1.0)
Fad3D	Glyma11g27190	Gm11:26,805,260..26,808,430	←	recent duplication Gm11 Ks0.2225 (Glyma v1.0)

Table S2 | Syntenic regions containing *FAD3* genes in soybean (Wm82.a2.v1) genome.

Synteny	Syntenic region (bp)	
	Coordinates	Size
FAD3A..FAD3B (Gm14..Gm02)	Gm14:43,488,986..48,040,552<br>Gm02:39,317,568..43,411,256	4,551,566<br>4,093,688
FAD3A..FAD3D (Gm14..Gm11)	Gm14:46,828,582..45,560,369<br>Gm11:14,593,258..23,026,887	1,731,787<br>8,433,629
FAD3A..FAD3C (Gm14..Gm18)	Gm14:45,569,552..48,040,552<br>Gm18:6,041,423..3,206,212	2,471,000<br>2,835,211
FAD3B..FAD3D (Gm02..Gm11)	Gm02:41,164,759..42,300,330<br>Gm11:23,026,887..14,576,517	1,135,571<br>8,450,373
FAD3B..FAD3C (Gm02..Gm18)	Gm02:41,175,313..44,532,815<br>Gm18:6,041,423..1,921,224	3,357,502<br>4,120,199
FAD3C..FAD3D (Gm18..Gm11)	Gm18:4,627,923..5,788,627<br>Gm11:14,448,495..24,249,278	1,160,704<br>9,800,782

Plant Genome Duplication Database (PGDD, http://chibba.agtec.uga.edu/duplication/index/locus)
Related syntenic regions in soybean genome by locus identifier
Display range: 500 Kb
Date accessed: 23.09.2015

<u>*FAD3A* (locus Glyma.14g194300) synteny</u>

Syntenic blocks: 52 (27 plant species)
Gene anchors: 6 - 248

[38] Glyma.14G194300 is contained in a huge block (Score 9852.0, *E*-value 2e-158) with 248 anchors

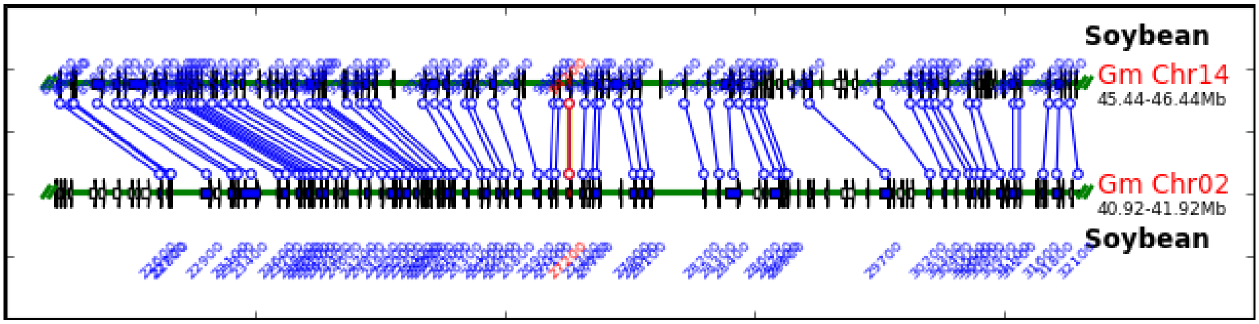

Order within Block	Locus 1	Locus 2	Ka	Ks
1	Glyma.02G208200	Glyma.14G176300	0.23	1.56
2	Glyma.02G208400	Glyma.14G176600	0.02	0.15
3	Glyma.02G208500	Glyma.14G176700	0.02	0.08
4	Glyma.02G208600	Glyma.14G176800	0.03	0.09
5	Glyma.02G208700	Glyma.14G176900	0.01	0.13

6	Glyma.02G208800	Glyma.14G177000	0.05	0.24
7	Glyma.02G208900	Glyma.14G177100	0.02	0.09
8	Glyma.02G209000	Glyma.14G177200	0.02	0.10
9	Glyma.02G209100	Glyma.14G177300	0.02	0.09
10	Glyma.02G209200	Glyma.14G177400	0.05	0.14
11	Glyma.02G209400	Glyma.14G177500	0.05	0.34
12	Glyma.02G209500	Glyma.14G177600	0.08	0.29
13	Glyma.02G209600	Glyma.14G177700	0.03	0.14
14	Glyma.02G210000	Glyma.14G177800	0.26	0.62
15	Glyma.02G210400	Glyma.14G177900	0.05	0.18
16	Glyma.02G210500	Glyma.14G178000	0.03	0.14
17	Glyma.02G210600	Glyma.14G178200	0.08	0.18
18	Glyma.02G210800	Glyma.14G178500	0.05	0.15
19	Glyma.02G211200	Glyma.14G178800	0.02	0.08
20	Glyma.02G211300	Glyma.14G178900	0.02	0.10
21	Glyma.02G211400	Glyma.14G179000	0.04	0.13
22	Glyma.02G211500	Glyma.14G179100	0.02	0.19
23	Glyma.02G211600	Glyma.14G179200	0.03	0.14
24	Glyma.02G211700	Glyma.14G179300	0.03	0.15
25	Glyma.02G211800	Glyma.14G179500	0.03	0.20
26	Glyma.02G211900	Glyma.14G179600	0.04	0.17
27	Glyma.02G212100	Glyma.14G179800	0.03	0.14
28	Glyma.02G212200	Glyma.14G179900	0.06	0.15
29	Glyma.02G212300	Glyma.14G180100	0.02	0.15
30	Glyma.02G212400	Glyma.14G180300	0.03	0.10
31	Glyma.02G212500	Glyma.14G180400	0.20	0.51
32	Glyma.02G212600	Glyma.14G180500	0.02	0.13
33	Glyma.02G212700	Glyma.14G180600	0.01	0.10
34	Glyma.02G213000	Glyma.14G180700	0.08	0.39
35	Glyma.02G213200	Glyma.14G180800	0.03	0.21
36	Glyma.02G213300	Glyma.14G180900	0.05	0.29
37	Glyma.02G213400	Glyma.14G181100	0.05	0.11
38	Glyma.02G213500	Glyma.14G181200	0.10	0.31
39	Glyma.02G213600	Glyma.14G181300	0.01	0.10
40	Glyma.02G213700	Glyma.14G181400	0.04	0.08
41	Glyma.02G213800	Glyma.14G181500	0.06	0.16
42	Glyma.02G213900	Glyma.14G181600	0.07	0.31
43	Glyma.02G214300	Glyma.14G181800	0.03	0.11

44	Glyma.02G214400	Glyma.14G181900	0.02	0.13
45	Glyma.02G214500	Glyma.14G182000	0.05	0.12
46	Glyma.02G214700	Glyma.14G182100	0.05	0.10
47	Glyma.02G215000	Glyma.14G182300	0.04	0.14
48	Glyma.02G215100	Glyma.14G182400	0.04	0.11
49	Glyma.02G215200	Glyma.14G182500	0.01	0.09
50	Glyma.02G215300	Glyma.14G182700	0.03	0.15
51	Glyma.02G215600	Glyma.14G182800	0.03	0.11
52	Glyma.02G215900	Glyma.14G182900	0.04	0.08
53	Glyma.02G216000	Glyma.14G183000	0.02	0.09
54	Glyma.02G216100	Glyma.14G183100	0.01	0.10
55	Glyma.02G216200	Glyma.14G183300	0.02	0.11
56	Glyma.02G216500	Glyma.14G183500	0.20	0.41
57	Glyma.02G216600	Glyma.14G183800	0.03	0.07
58	Glyma.02G216800	Glyma.14G184100	0.65	0.60
59	Glyma.02G216900	Glyma.14G184200	0.03	0.08
60	Glyma.02G217000	Glyma.14G184400	0.04	0.07
61	Glyma.02G217100	Glyma.14G184500	0.02	0.10
62	Glyma.02G217200	Glyma.14G184600	0.02	0.08
63	Glyma.02G217500	Glyma.14G184700	0.06	0.17
64	Glyma.02G217800	Glyma.14G185100	0.02	0.12
65	Glyma.02G217900	Glyma.14G185300	0.04	0.14
66	Glyma.02G218100	Glyma.14G185400	0.03	0.14
67	Glyma.02G218200	Glyma.14G185600	0.05	0.23
68	Glyma.02G218300	Glyma.14G185700	0.01	0.11
69	Glyma.02G219000	Glyma.14G186300	0.04	0.10
70	Glyma.02G219100	Glyma.14G186400	0.09	0.15
71	Glyma.02G219200	Glyma.14G186500	0.11	0.22
72	Glyma.02G219300	Glyma.14G186700	0.14	0.22
73	Glyma.02G219400	Glyma.14G186800	0.05	0.11
74	Glyma.02G220200	Glyma.14G187600	0.23	0.38
75	Glyma.02G220800	Glyma.14G188200	0.02	0.17
76	Glyma.02G220900	Glyma.14G188400	0.02	0.12
77	Glyma.02G221000	Glyma.14G188500	0.02	0.13
78	Glyma.02G221100	Glyma.14G188600	0.02	0.16
79	Glyma.02G221200	Glyma.14G188700	0.03	0.16
80	Glyma.02G221300	Glyma.14G188800	0.04	0.08
81	Glyma.02G221700	Glyma.14G189000	0.03	0.16

82	Glyma.02G221900	Glyma.14G189100	0.02	0.15
83	Glyma.02G222000	Glyma.14G189200	0.09	0.29
84	Glyma.02G222300	Glyma.14G189300	0.05	0.13
85	Glyma.02G222400	Glyma.14G189400	0.02	0.12
86	Glyma.02G222600	Glyma.14G189500	0.02	0.14
87	Glyma.02G222700	Glyma.14G189600	0.08	0.14
88	Glyma.02G222800	Glyma.14G189700	0.09	0.38
89	Glyma.02G222900	Glyma.14G189900	0.02	0.07
90	Glyma.02G223100	Glyma.14G190000	0.01	0.10
91	Glyma.02G223300	Glyma.14G190100	0.02	0.13
92	Glyma.02G223400	Glyma.14G190200	0.02	0.08
93	Glyma.02G223600	Glyma.14G190300	0.02	0.20
94	Glyma.02G223700	Glyma.14G190400	0.02	0.09
95	Glyma.02G223800	Glyma.14G190500	0.06	0.13
96	Glyma.02G223900	Glyma.14G190600	0.02	0.13
97	Glyma.02G224000	Glyma.14G190700	0.02	0.11
98	Glyma.02G224100	Glyma.14G190800	0.00	0.59
99	Glyma.02G224200	Glyma.14G190900	0.03	0.14
100	Glyma.02G224300	Glyma.14G191000	0.08	0.13
101	Glyma.02G224400	Glyma.14G191100	0.05	0.18
102	Glyma.02G224600	Glyma.14G191300	0.04	0.17
103	Glyma.02G224700	Glyma.14G191400	0.09	0.21
104	Glyma.02G224800	Glyma.14G191500	0.01	0.14
105	Glyma.02G224900	Glyma.14G191700	0.02	0.12
106	Glyma.02G225000	Glyma.14G191900	0.04	0.11
107	Glyma.02G225200	Glyma.14G192000	0.07	0.10
108	Glyma.02G225300	Glyma.14G192100	0.02	0.08
109	Glyma.02G225400	Glyma.14G192200	0.04	0.13
110	Glyma.02G225500	Glyma.14G192300	0.03	0.12
111	Glyma.02G225600	Glyma.14G192400	0.04	0.10
112	Glyma.02G225700	Glyma.14G192600	0.10	0.20
113	Glyma.02G225800	Glyma.14G192800	0.09	0.23
114	Glyma.02G226100	Glyma.14G192900	0.05	0.10
115	Glyma.02G226200	Glyma.14G193000	0.06	0.21
116	Glyma.02G226300	Glyma.14G193200	0.02	0.11
117	Glyma.02G226400	Glyma.14G193300	0.04	0.10
118	Glyma.02G226500	Glyma.14G193400	0.02	0.12
119	Glyma.02G226600	Glyma.14G193500	0.03	0.18

120	Glyma.02G226700	Glyma.14G193600	0.03	0.11
121	Glyma.02G226800	Glyma.14G193800	0.04	0.12
122	Glyma.02G226900	Glyma.14G193900	0.03	0.16
123	Glyma.02G227000	Glyma.14G194100	0.04	0.13
124	Glyma.02G227100	Glyma.14G194200	0.07	0.15
125	Glyma.02G227200	Glyma.14G194300	0.02	0.13
126	Glyma.02G227300	Glyma.14G194400	0.02	0.11
127	Glyma.02G227600	Glyma.14G194500	0.03	0.15
128	Glyma.02G227700	Glyma.14G194600	0.02	0.08
129	Glyma.02G227900	Glyma.14G194800	0.06	0.13
130	Glyma.02G228000	Glyma.14G194900	0.05	0.09
131	Glyma.02G228100	Glyma.14G195000	0.01	0.08
132	Glyma.02G228200	Glyma.14G195200	0.02	0.12
133	Glyma.02G228300	Glyma.14G195300	0.08	0.16
134	Glyma.02G228400	Glyma.14G195400	0.01	0.10
135	Glyma.02G228600	Glyma.14G195600	0.04	0.09
136	Glyma.02G228700	Glyma.14G195700	0.02	0.09
137	Glyma.02G228800	Glyma.14G195900	0.06	0.19
138	Glyma.02G228900	Glyma.14G196000	0.12	0.24
139	Glyma.02G229700	Glyma.14G196800	0.02	0.11
140	Glyma.02G230200	Glyma.14G197300	0.04	0.38
141	Glyma.02G230300	Glyma.14G197400	0.07	0.16
142	Glyma.02G230400	Glyma.14G197500	0.06	0.13
143	Glyma.02G230500	Glyma.14G197600	0.01	0.12
144	Glyma.02G230600	Glyma.14G197700	0.09	0.17
145	Glyma.02G230700	Glyma.14G197800	0.05	0.11
146	Glyma.02G230800	Glyma.14G197900	0.07	0.17
147	Glyma.02G230900	Glyma.14G198000	0.42	1.63
148	Glyma.02G231100	Glyma.14G198700	0.03	0.09
149	Glyma.02G231200	Glyma.14G198800	0.02	0.21
150	Glyma.02G231600	Glyma.14G198900	0.01	0.20
151	Glyma.02G231800	Glyma.14G199000	0.03	0.17
152	Glyma.02G232100	Glyma.14G199200	0.03	0.21
153	Glyma.02G232500	Glyma.14G199900	0.11	0.25
154	Glyma.02G232600	Glyma.14G200200	0.02	0.12
155	Glyma.02G232700	Glyma.14G200300	0.07	0.21
156	Glyma.02G232800	Glyma.14G200400	0.01	0.10
157	Glyma.02G233000	Glyma.14G200600	0.03	0.11

158	Glyma.02G233100	Glyma.14G200700	0.05	0.15
159	Glyma.02G233200	Glyma.14G201200	0.05	0.16
160	Glyma.02G233300	Glyma.14G201300	0.04	0.13
161	Glyma.02G233700	Glyma.14G201500	0.02	0.10
162	Glyma.02G233800	Glyma.14G201600	0.03	0.15
163	Glyma.02G233900	Glyma.14G201700	0.18	0.72
164	Glyma.02G234200	Glyma.14G201800	0.03	0.10
165	Glyma.02G234300	Glyma.14G202000	0.04	0.16
166	Glyma.02G234500	Glyma.14G202100	0.03	0.18
167	Glyma.02G234600	Glyma.14G202300	0.03	0.09
168	Glyma.02G234800	Glyma.14G202700	0.07	0.29
169	Glyma.02G234900	Glyma.14G202800	0.09	0.15
170	Glyma.02G235000	Glyma.14G202900	0.23	0.38
171	Glyma.02G235100	Glyma.14G203000	0.02	0.20
172	Glyma.02G235400	Glyma.14G203400	0.04	0.13
173	Glyma.02G235500	Glyma.14G203500	0.05	0.24
174	Glyma.02G235600	Glyma.14G203600	0.04	0.13
175	Glyma.02G235700	Glyma.14G203700	0.02	0.12
176	Glyma.02G235800	Glyma.14G203800	0.04	0.09
177	Glyma.02G235900	Glyma.14G203900	0.01	0.10
178	Glyma.02G236100	Glyma.14G204000	0.09	0.19
179	Glyma.02G236200	Glyma.14G204100	0.03	0.15
180	Glyma.02G236300	Glyma.14G204200	0.03	0.17
181	Glyma.02G236400	Glyma.14G204400	0.05	0.12
182	Glyma.02G236600	Glyma.14G205100	0.03	0.12
183	Glyma.02G236700	Glyma.14G205500	0.01	0.06
184	Glyma.02G236800	Glyma.14G205600	0.05	0.23
185	Glyma.02G236900	Glyma.14G205700	0.00	0.14
186	Glyma.02G237000	Glyma.14G206000	0.03	0.18
187	Glyma.02G237100	Glyma.14G206100	0.03	0.15
188	Glyma.02G237200	Glyma.14G206200	0.14	0.28
189	Glyma.02G237400	Glyma.14G206500	0.23	0.28
190	Glyma.02G237600	Glyma.14G206600	0.10	0.16
191	Glyma.02G237700	Glyma.14G206700	0.10	0.34
192	Glyma.02G237800	Glyma.14G206800	0.03	0.11
193	Glyma.02G238300	Glyma.14G207200	0.01	0.13
194	Glyma.02G238400	Glyma.14G207300	0.01	0.07
195	Glyma.02G238500	Glyma.14G207400	0.06	0.15

196	Glyma.02G238600	Glyma.14G207500	0.03	0.15
197	Glyma.02G238700	Glyma.14G207600	0.02	0.16
198	Glyma.02G238800	Glyma.14G207700	0.02	0.21
199	Glyma.02G239000	Glyma.14G207900	0.05	0.14
200	Glyma.02G239100	Glyma.14G208000	0.02	0.15
201	Glyma.02G239200	Glyma.14G208100	0.04	0.08
202	Glyma.02G239300	Glyma.14G208200	0.04	0.31
203	Glyma.02G239600	Glyma.14G208500	0.01	0.11
204	Glyma.02G239700	Glyma.14G208600	0.00	0.18
205	Glyma.02G239800	Glyma.14G209000	0.07	0.11
206	Glyma.02G239900	Glyma.14G209200	0.08	0.13
207	Glyma.02G240000	Glyma.14G209400	0.03	0.13
208	Glyma.02G240200	Glyma.14G209700	0.06	0.15
209	Glyma.02G240300	Glyma.14G209800	0.06	0.15
210	Glyma.02G240400	Glyma.14G209900	0.01	0.10
211	Glyma.02G240500	Glyma.14G210000	0.03	0.14
212	Glyma.02G240600	Glyma.14G210100	0.01	0.15
213	Glyma.02G240700	Glyma.14G210200	0.02	0.09
214	Glyma.02G240800	Glyma.14G210400	0.02	0.15
215	Glyma.02G240900	Glyma.14G210500	0.02	0.19
216	Glyma.02G241000	Glyma.14G210600	0.05	0.13
217	Glyma.02G241100	Glyma.14G210700	0.03	0.14
218	Glyma.02G241200	Glyma.14G211000	0.00	0.11
219	Glyma.02G241400	Glyma.14G211100	0.02	0.12
220	Glyma.02G241500	Glyma.14G211200	0.02	0.18
221	Glyma.02G241600	Glyma.14G211300	0.04	0.10
222	Glyma.02G241700	Glyma.14G211400	0.06	0.09
223	Glyma.02G241800	Glyma.14G211500	0.03	0.11
224	Glyma.02G241900	Glyma.14G211600	0.02	0.12
225	Glyma.02G242000	Glyma.14G211800	0.02	0.09
226	Glyma.02G242100	Glyma.14G211900	0.05	0.14
227	Glyma.02G242400	Glyma.14G212000	0.02	0.12
228	Glyma.02G242700	Glyma.14G212100	0.08	0.22
229	Glyma.02G242900	Glyma.14G212200	0.04	0.17
230	Glyma.02G243200	Glyma.14G212300	0.04	0.10
231	Glyma.02G243300	Glyma.14G212400	0.08	0.13
232	Glyma.02G243400	Glyma.14G212500	0.02	0.10
233	Glyma.02G243500	Glyma.14G212600	0.05	0.12

234	Glyma.02G243600	Glyma.14G212700	0.04	0.21
235	Glyma.02G243800	Glyma.14G212900	0.04	0.08
236	Glyma.02G243900	Glyma.14G213200	1.11	1.23
237	Glyma.02G244100	Glyma.14G213400	0.02	0.09
238	Glyma.02G244200	Glyma.14G213600	0.04	0.19
239	Glyma.02G244700	Glyma.14G214300	0.03	0.17
240	Glyma.02G245000	Glyma.14G215100	0.04	0.21
241	Glyma.02G245200	Glyma.14G215300	0.04	0.12
242	Glyma.02G245300	Glyma.14G215400	0.02	0.05
243	Glyma.02G245400	Glyma.14G215500	0.02	0.11
244	Glyma.02G245600	Glyma.14G215600	0.01	0.08
245	Glyma.02G245700	Glyma.14G215800	0.03	0.21
246	Glyma.02G245800	Glyma.14G215900	0.03	0.08
247	Glyma.02G245900	Glyma.14G216000	0.03	0.11
248	Glyma.02G246000	Glyma.14G216100	0.03	0.12

[40] Glyma.14G194300 is contained in a large block (Score 1483.0, *E*-value 8e-179) with 39 anchors

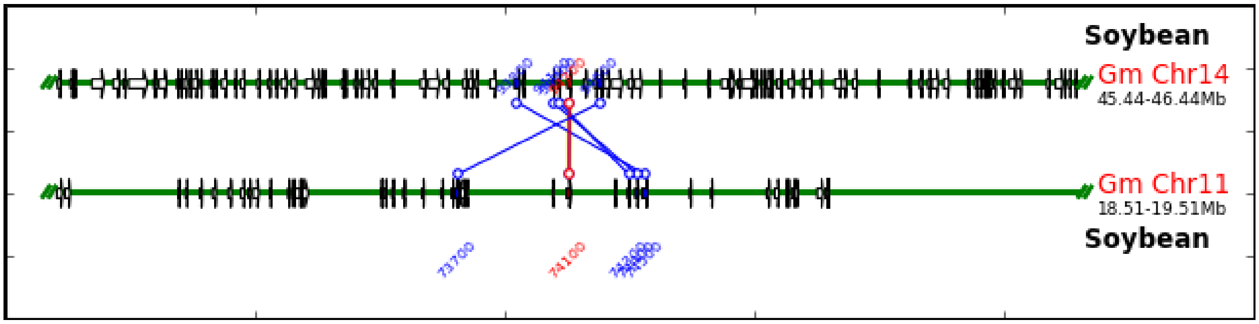

Order within Block	Locus 1	Locus 2	Ka	Ks
1	Glyma.11G159900	Glyma.14G203700	0.20	0.59
2	Glyma.11G160600	Glyma.14G202900	0.47	1.36
3	Glyma.11G161000	Glyma.14G202800	0.30	1.10
4	Glyma.11G161300	Glyma.14G202700	0.16	0.50
5	Glyma.11G161500	Glyma.14G202000	0.11	0.54
6	Glyma.11G161600	Glyma.14G201800	0.23	0.52
7	Glyma.11G161800	Glyma.14G201700	0.13	0.83
8	Glyma.11G162100	Glyma.14G201600	0.13	0.60
9	Glyma.11G162400	Glyma.14G201500	0.14	0.45

10	Glyma.11G162500	Glyma.14G201300	0.24	0.54
11	Glyma.11G163100	Glyma.14G200500	0.26	1.40
12	Glyma.11G163300	Glyma.14G200200	0.20	0.67
13	Glyma.11G163600	Glyma.14G200000	0.75	4.21
14	Glyma.11G163800	Glyma.14G199900	0.27	1.09
15	Glyma.11G164000	Glyma.14G198900	0.10	0.49
16	Glyma.11G164600	Glyma.14G198000	0.45	1.63
17	Glyma.11G165000	Glyma.14G197700	0.15	0.47
18	Glyma.11G165700	Glyma.14G197300	0.17	1.27
19	Glyma.11G169300	Glyma.14G196200	0.16	1.13
20	Glyma.11G169600	Glyma.14G195900	0.29	1.13
21	Glyma.11G169700	Glyma.14G195700	0.12	0.45
22	Glyma.11G169800	Glyma.14G195600	0.16	0.53
23	Glyma.11G170000	Glyma.14G195300	0.33	0.69
24	Glyma.11G170200	Glyma.14G195200	0.14	0.69
25	Glyma.11G170300	Glyma.14G195000	0.05	0.60
26	Glyma.11G170500	Glyma.14G194900	0.12	0.71
27	Glyma.11G173700	Glyma.14G194600	0.13	0.61
28	Glyma.11G174100	Glyma.14G194300	0.15	0.96
29	Glyma.11G174300	Glyma.14G194200	0.29	0.50
30	Glyma.11G174400	Glyma.14G194100	0.19	0.59
31	Glyma.11G174500	Glyma.14G193900	0.22	0.59
32	Glyma.11G175900	Glyma.14G192500	0.14	1.11
33	Glyma.11G176100	Glyma.14G192400	0.30	0.32
34	Glyma.11G176400	Glyma.14G191900	0.16	0.61
35	Glyma.11G176500	Glyma.14G191700	0.14	0.92
36	Glyma.11G176800	Glyma.14G191300	0.15	0.58
37	Glyma.11G176900	Glyma.14G191100	0.40	0.91
38	Glyma.11G177800	Glyma.14G190800	0.01	1.04
39	Glyma.11G178300	Glyma.14G190700	0.20	0.48

[42] Glyma.14G194300 is contained in a huge block (Score 3013.0, *E*-value 1e-101) with 78 anchors

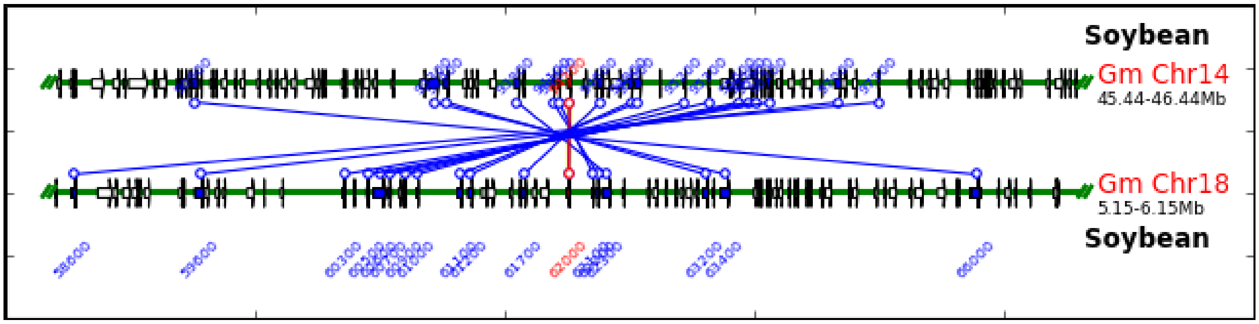

Order within Block	Locus 1	Annotation 1	Locus 2	Annotation 2	Ka	Ks
1	Glyma.14G190900		Glyma.18G066000		0.67	2.15
2	Glyma.14G193300		Glyma.18G063400		0.17	0.79
3	Glyma.14G193400		Glyma.18G063200		0.14	0.51
4	Glyma.14G193900		Glyma.18G062300		0.19	0.64
5	Glyma.14G194100		Glyma.18G062200		0.19	0.57
6	Glyma.14G194200		Glyma.18G062100		0.30	0.50
7	Glyma.14G194300		Glyma.18G062000		0.14	0.84
8	Glyma.14G194600		Glyma.18G061700		0.12	0.56
9	Glyma.14G194900		Glyma.18G061200		0.14	0.61
10	Glyma.14G195000		Glyma.18G061100		0.06	0.53
11	Glyma.14G195200		Glyma.18G061000		0.15	0.68
12	Glyma.14G195300		Glyma.18G060900		0.35	0.68
13	Glyma.14G195600		Glyma.18G060700		0.15	0.47
14	Glyma.14G195700		Glyma.18G060600		0.12	0.44
15	Glyma.14G195900		Glyma.18G060500		0.29	0.95
16	Glyma.14G196200		Glyma.18G060300		0.15	1.09
17	Glyma.14G197000		Glyma.18G059600		0.20	0.56
18	Glyma.14G197300		Glyma.18G058600		0.20	1.37
19	Glyma.14G197700		Glyma.18G058200		0.15	0.45
20	Glyma.14G198000		Glyma.18G057800		0.47	2.05
21	Glyma.14G198900		Glyma.18G057200		0.10	0.50
22	Glyma.14G199900		Glyma.18G057000		0.29	1.51
23	Glyma.14G200000		Glyma.18G056800		0.69	3.17
24	Glyma.14G200200		Glyma.18G056600		0.19	0.64
25	Glyma.14G200500		Glyma.18G056400		0.26	1.16
26	Glyma.14G201200		Glyma.18G055900		0.14	0.50
27	Glyma.14G201300		Glyma.18G055800		0.30	0.67
28	Glyma.14G201500		Glyma.18G055700		0.12	0.56

29	Glyma.14G201600		Glyma.18G055600		0.12	0.56
30	Glyma.14G201700		Glyma.18G055400		0.13	0.82
31	Glyma.14G201800		Glyma.18G055300		0.23	0.57
32	Glyma.14G202000		Glyma.18G055200		0.11	0.54
33	Glyma.14G202700		Glyma.18G055000		0.13	0.53
34	Glyma.14G202800		Glyma.18G054800		0.32	1.36
35	Glyma.14G203000		Glyma.18G054600		0.24	1.02
36	Glyma.14G203500		Glyma.18G054300		0.47	0.76
37	Glyma.14G203600		Glyma.18G054200		0.15	0.54
38	Glyma.14G203700		Glyma.18G054100		0.17	0.55
39	Glyma.14G203900		Glyma.18G054000		0.07	0.59
40	Glyma.14G204100		Glyma.18G052500		0.14	0.74
41	Glyma.14G204200		Glyma.18G052300		0.29	0.69
42	Glyma.14G204400		Glyma.18G052100		0.23	0.63
43	Glyma.14G205100		Glyma.18G051600		0.25	0.88
44	Glyma.14G205600		Glyma.18G051500		0.38	0.84
45	Glyma.14G205700		Glyma.18G051400		0.01	0.63
46	Glyma.14G206000		Glyma.18G051100		0.12	0.59
47	Glyma.14G206100		Glyma.18G050800		0.17	0.82
48	Glyma.14G206200		Glyma.18G050700		0.21	0.48
49	Glyma.14G206600		Glyma.18G050400		0.07	0.51
50	Glyma.14G206800		Glyma.18G050300		0.10	0.45
51	Glyma.14G207400		Glyma.18G048800		0.19	0.73
52	Glyma.14G207500		Glyma.18G048700		0.15	0.51
53	Glyma.14G207800		Glyma.18G048400		0.09	0.94
54	Glyma.14G207900		Glyma.18G048000		0.26	0.76
55	Glyma.14G208000		Glyma.18G047800		0.19	0.74
56	Glyma.14G208400		Glyma.18G047000		0.25	0.73
57	Glyma.14G208500		Glyma.18G046800		0.06	0.46
58	Glyma.14G208700		Glyma.18G045600		0.49	2.01
59	Glyma.14G208900		Glyma.18G045300		0.22	0.75
60	Glyma.14G209000		Glyma.18G045200		0.34	0.56
61	Glyma.14G209400		Glyma.18G045100		0.13	0.47
62	Glyma.14G209600		Glyma.18G044900		0.15	1.11
63	Glyma.14G210600		Glyma.18G044200		0.23	0.59
64	Glyma.14G211100		Glyma.18G043600		0.05	0.77
65	Glyma.14G211200		Glyma.18G043500		0.14	0.76
66	Glyma.14G211900		Glyma.18G043000		0.20	0.69

67	Glyma.14G212000		Glyma.18G042300		0.13	0.50
68	Glyma.14G212100		Glyma.18G042200		0.15	0.73
69	Glyma.14G212200		Glyma.18G042100		0.24	0.91
70	Glyma.14G212700		Glyma.18G041400		0.18	0.79
71	Glyma.14G213100		Glyma.18G041300		0.44	0.73
72	Glyma.14G213200		Glyma.18G041100		0.14	0.63
73	Glyma.14G213400		Glyma.18G040900		0.08	0.46
74	Glyma.14G213600		Glyma.18G040800		0.23	0.88
75	Glyma.14G214500		Glyma.18G040700		0.14	0.53
76	Glyma.14G215100		Glyma.18G040200		0.25	1.11
77	Glyma.14G215800		Glyma.18G039500		0.14	0.64
78	Glyma.14G216100		Glyma.18G039400		0.15	0.59

***FAD3B* (locus Glyma.02g227200)**

Syntenic blocks: 49 (25 plant species)
Gene anchors: 6 - 254

[37] Glyma.02G227200 is contained in a large block (Score 1688.0, *E*-value 0.0) with 44 anchors

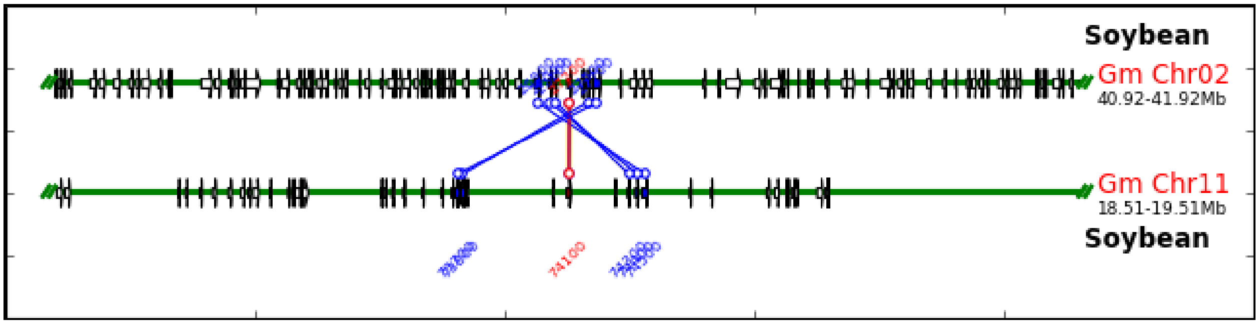

Order within Block	Locus 1	Locus 2	Ka	Ks
1	Glyma.02G224000	Glyma.11G178300	0.21	0.44
2	Glyma.02G224100	Glyma.11G177800	0.01	0.99
3	Glyma.02G224400	Glyma.11G176900	0.56	1.10
4	Glyma.02G224600	Glyma.11G176800	0.16	0.62
5	Glyma.02G224900	Glyma.11G176500	0.16	0.90
6	Glyma.02G225000	Glyma.11G176400	0.15	0.62
7	Glyma.02G225600	Glyma.11G176100	0.28	0.40
8	Glyma.02G226900	Glyma.11G174500	0.21	0.52
9	Glyma.02G227000	Glyma.11G174400	0.19	0.58
10	Glyma.02G227100	Glyma.11G174300	0.30	0.52
11	Glyma.02G227200	Glyma.11G174100	0.15	0.90
12	Glyma.02G227500	Glyma.11G173800	0.17	0.53
13	Glyma.02G227700	Glyma.11G173700	0.13	0.60
14	Glyma.02G227900	Glyma.11G170900	0.29	0.69
15	Glyma.02G228000	Glyma.11G170500	0.15	0.81
16	Glyma.02G228100	Glyma.11G170300	0.06	0.61
17	Glyma.02G228200	Glyma.11G170200	0.14	0.71
18	Glyma.02G228300	Glyma.11G170000	0.36	0.69
19	Glyma.02G228500	Glyma.11G169900	0.13	0.41
20	Glyma.02G228600	Glyma.11G169800	0.17	0.56
21	Glyma.02G228700	Glyma.11G169700	0.12	0.44
22	Glyma.02G228800	Glyma.11G169600	0.30	0.91
23	Glyma.02G229200	Glyma.11G168800	0.13	0.56

24	Glyma.02G229400	Glyma.11G167200	0.12	0.54
25	Glyma.02G229500	Glyma.11G167100	0.21	0.63
26	Glyma.02G229600	Glyma.11G166300	0.26	0.77
27	Glyma.02G229700	Glyma.11G166100	0.08	0.59
28	Glyma.02G229900	Glyma.11G166000	0.13	0.70
29	Glyma.02G230200	Glyma.11G165700	0.18	1.10
30	Glyma.02G230600	Glyma.11G165000	0.14	0.50
31	Glyma.02G230900	Glyma.11G164600	0.39	1.10
32	Glyma.02G231600	Glyma.11G164000	0.10	0.59
33	Glyma.02G232500	Glyma.11G163800	0.22	0.97
34	Glyma.02G232600	Glyma.11G163300	0.18	0.67
35	Glyma.02G233300	Glyma.11G162500	0.23	0.52
36	Glyma.02G233700	Glyma.11G162400	0.14	0.38
37	Glyma.02G233800	Glyma.11G162100	0.12	0.67
38	Glyma.02G233900	Glyma.11G161800	0.16	0.87
39	Glyma.02G234200	Glyma.11G161600	0.19	0.50
40	Glyma.02G234300	Glyma.11G161500	0.16	0.47
41	Glyma.02G234800	Glyma.11G161300	0.14	0.62
42	Glyma.02G234900	Glyma.11G161000	0.27	1.47
43	Glyma.02G235000	Glyma.11G160600	0.39	0.97
44	Glyma.02G235600	Glyma.11G159800	0.16	0.71

[38] Glyma.02G227200 is contained in a huge block (Score 9852.0, *E*-value 2e-158) with 248 anchors

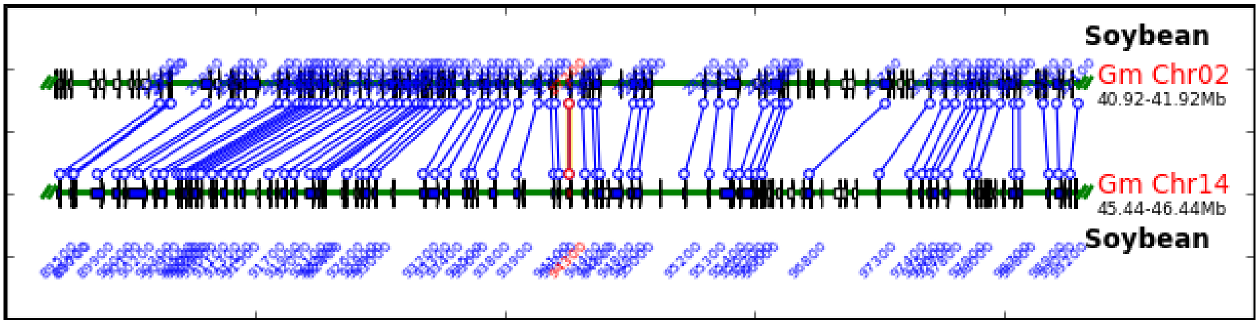

Order within Block	Locus 1	Locus 2	Ka	Ks
1	Glyma.02G208200	Glyma.14G176300	0.23	1.56
2	Glyma.02G208400	Glyma.14G176600	0.02	0.15
3	Glyma.02G208500	Glyma.14G176700	0.02	0.08
4	Glyma.02G208600	Glyma.14G176800	0.03	0.09

5	Glyma.02G208700	Glyma.14G176900	0.01	0.13
6	Glyma.02G208800	Glyma.14G177000	0.05	0.24
7	Glyma.02G208900	Glyma.14G177100	0.02	0.09
8	Glyma.02G209000	Glyma.14G177200	0.02	0.10
9	Glyma.02G209100	Glyma.14G177300	0.02	0.09
10	Glyma.02G209200	Glyma.14G177400	0.05	0.14
11	Glyma.02G209400	Glyma.14G177500	0.05	0.34
12	Glyma.02G209500	Glyma.14G177600	0.08	0.29
13	Glyma.02G209600	Glyma.14G177700	0.03	0.14
14	Glyma.02G210000	Glyma.14G177800	0.26	0.62
15	Glyma.02G210400	Glyma.14G177900	0.05	0.18
16	Glyma.02G210500	Glyma.14G178000	0.03	0.14
17	Glyma.02G210600	Glyma.14G178200	0.08	0.18
18	Glyma.02G210800	Glyma.14G178500	0.05	0.15
19	Glyma.02G211200	Glyma.14G178800	0.02	0.08
20	Glyma.02G211300	Glyma.14G178900	0.02	0.10
21	Glyma.02G211400	Glyma.14G179000	0.04	0.13
22	Glyma.02G211500	Glyma.14G179100	0.02	0.19
23	Glyma.02G211600	Glyma.14G179200	0.03	0.14
24	Glyma.02G211700	Glyma.14G179300	0.03	0.15
25	Glyma.02G211800	Glyma.14G179500	0.03	0.20
26	Glyma.02G211900	Glyma.14G179600	0.04	0.17
27	Glyma.02G212100	Glyma.14G179800	0.03	0.14
28	Glyma.02G212200	Glyma.14G179900	0.06	0.15
29	Glyma.02G212300	Glyma.14G180100	0.02	0.15
30	Glyma.02G212400	Glyma.14G180300	0.03	0.10
31	Glyma.02G212500	Glyma.14G180400	0.20	0.51
32	Glyma.02G212600	Glyma.14G180500	0.02	0.13
33	Glyma.02G212700	Glyma.14G180600	0.01	0.10
34	Glyma.02G213000	Glyma.14G180700	0.08	0.39
35	Glyma.02G213200	Glyma.14G180800	0.03	0.21
36	Glyma.02G213300	Glyma.14G180900	0.05	0.29
37	Glyma.02G213400	Glyma.14G181100	0.05	0.11
38	Glyma.02G213500	Glyma.14G181200	0.10	0.31
39	Glyma.02G213600	Glyma.14G181300	0.01	0.10
40	Glyma.02G213700	Glyma.14G181400	0.04	0.08
41	Glyma.02G213800	Glyma.14G181500	0.06	0.16
42	Glyma.02G213900	Glyma.14G181600	0.07	0.31

43	Glyma.02G214300	Glyma.14G181800	0.03	0.11
44	Glyma.02G214400	Glyma.14G181900	0.02	0.13
45	Glyma.02G214500	Glyma.14G182000	0.05	0.12
46	Glyma.02G214700	Glyma.14G182100	0.05	0.10
47	Glyma.02G215000	Glyma.14G182300	0.04	0.14
48	Glyma.02G215100	Glyma.14G182400	0.04	0.11
49	Glyma.02G215200	Glyma.14G182500	0.01	0.09
50	Glyma.02G215300	Glyma.14G182700	0.03	0.15
51	Glyma.02G215600	Glyma.14G182800	0.03	0.11
52	Glyma.02G215900	Glyma.14G182900	0.04	0.08
53	Glyma.02G216000	Glyma.14G183000	0.02	0.09
54	Glyma.02G216100	Glyma.14G183100	0.01	0.10
55	Glyma.02G216200	Glyma.14G183300	0.02	0.11
56	Glyma.02G216500	Glyma.14G183500	0.20	0.41
57	Glyma.02G216600	Glyma.14G183800	0.03	0.07
58	Glyma.02G216800	Glyma.14G184100	0.65	0.60
59	Glyma.02G216900	Glyma.14G184200	0.03	0.08
60	Glyma.02G217000	Glyma.14G184400	0.04	0.07
61	Glyma.02G217100	Glyma.14G184500	0.02	0.10
62	Glyma.02G217200	Glyma.14G184600	0.02	0.08
63	Glyma.02G217500	Glyma.14G184700	0.06	0.17
64	Glyma.02G217800	Glyma.14G185100	0.02	0.12
65	Glyma.02G217900	Glyma.14G185300	0.04	0.14
66	Glyma.02G218100	Glyma.14G185400	0.03	0.14
67	Glyma.02G218200	Glyma.14G185600	0.05	0.23
68	Glyma.02G218300	Glyma.14G185700	0.01	0.11
69	Glyma.02G219000	Glyma.14G186300	0.04	0.10
70	Glyma.02G219100	Glyma.14G186400	0.09	0.15
71	Glyma.02G219200	Glyma.14G186500	0.11	0.22
72	Glyma.02G219300	Glyma.14G186700	0.14	0.22
73	Glyma.02G219400	Glyma.14G186800	0.05	0.11
74	Glyma.02G220200	Glyma.14G187600	0.23	0.38
75	Glyma.02G220800	Glyma.14G188200	0.02	0.17
76	Glyma.02G220900	Glyma.14G188400	0.02	0.12
77	Glyma.02G221000	Glyma.14G188500	0.02	0.13
78	Glyma.02G221100	Glyma.14G188600	0.02	0.16
79	Glyma.02G221200	Glyma.14G188700	0.03	0.16
80	Glyma.02G221300	Glyma.14G188800	0.04	0.08

81	Glyma.02G221700	Glyma.14G189000	0.03	0.16
82	Glyma.02G221900	Glyma.14G189100	0.02	0.15
83	Glyma.02G222000	Glyma.14G189200	0.09	0.29
84	Glyma.02G222300	Glyma.14G189300	0.05	0.13
85	Glyma.02G222400	Glyma.14G189400	0.02	0.12
86	Glyma.02G222600	Glyma.14G189500	0.02	0.14
87	Glyma.02G222700	Glyma.14G189600	0.08	0.14
88	Glyma.02G222800	Glyma.14G189700	0.09	0.38
89	Glyma.02G222900	Glyma.14G189900	0.02	0.07
90	Glyma.02G223100	Glyma.14G190000	0.01	0.10
91	Glyma.02G223300	Glyma.14G190100	0.02	0.13
92	Glyma.02G223400	Glyma.14G190200	0.02	0.08
93	Glyma.02G223600	Glyma.14G190300	0.02	0.20
94	Glyma.02G223700	Glyma.14G190400	0.02	0.09
95	Glyma.02G223800	Glyma.14G190500	0.06	0.13
96	Glyma.02G223900	Glyma.14G190600	0.02	0.13
97	Glyma.02G224000	Glyma.14G190700	0.02	0.11
98	Glyma.02G224100	Glyma.14G190800	0.00	0.59
99	Glyma.02G224200	Glyma.14G190900	0.03	0.14
100	Glyma.02G224300	Glyma.14G191000	0.08	0.13
101	Glyma.02G224400	Glyma.14G191100	0.05	0.18
102	Glyma.02G224600	Glyma.14G191300	0.04	0.17
103	Glyma.02G224700	Glyma.14G191400	0.09	0.21
104	Glyma.02G224800	Glyma.14G191500	0.01	0.14
105	Glyma.02G224900	Glyma.14G191700	0.02	0.12
106	Glyma.02G225000	Glyma.14G191900	0.04	0.11
107	Glyma.02G225200	Glyma.14G192000	0.07	0.10
108	Glyma.02G225300	Glyma.14G192100	0.02	0.08
109	Glyma.02G225400	Glyma.14G192200	0.04	0.13
110	Glyma.02G225500	Glyma.14G192300	0.03	0.12
111	Glyma.02G225600	Glyma.14G192400	0.04	0.10
112	Glyma.02G225700	Glyma.14G192600	0.10	0.20
113	Glyma.02G225800	Glyma.14G192800	0.09	0.23
114	Glyma.02G226100	Glyma.14G192900	0.05	0.10
115	Glyma.02G226200	Glyma.14G193000	0.06	0.21
116	Glyma.02G226300	Glyma.14G193200	0.02	0.11
117	Glyma.02G226400	Glyma.14G193300	0.04	0.10
118	Glyma.02G226500	Glyma.14G193400	0.02	0.12

119	Glyma.02G226600	Glyma.14G193500	0.03	0.18
120	Glyma.02G226700	Glyma.14G193600	0.03	0.11
121	Glyma.02G226800	Glyma.14G193800	0.04	0.12
122	Glyma.02G226900	Glyma.14G193900	0.03	0.16
123	Glyma.02G227000	Glyma.14G194100	0.04	0.13
124	Glyma.02G227100	Glyma.14G194200	0.07	0.15
125	Glyma.02G227200	Glyma.14G194300	0.02	0.13
126	Glyma.02G227300	Glyma.14G194400	0.02	0.11
127	Glyma.02G227600	Glyma.14G194500	0.03	0.15
128	Glyma.02G227700	Glyma.14G194600	0.02	0.08
129	Glyma.02G227900	Glyma.14G194800	0.06	0.13
130	Glyma.02G228000	Glyma.14G194900	0.05	0.09
131	Glyma.02G228100	Glyma.14G195000	0.01	0.08
132	Glyma.02G228200	Glyma.14G195200	0.02	0.12
133	Glyma.02G228300	Glyma.14G195300	0.08	0.16
134	Glyma.02G228400	Glyma.14G195400	0.01	0.10
135	Glyma.02G228600	Glyma.14G195600	0.04	0.09
136	Glyma.02G228700	Glyma.14G195700	0.02	0.09
137	Glyma.02G228800	Glyma.14G195900	0.06	0.19
138	Glyma.02G228900	Glyma.14G196000	0.12	0.24
139	Glyma.02G229700	Glyma.14G196800	0.02	0.11
140	Glyma.02G230200	Glyma.14G197300	0.04	0.38
141	Glyma.02G230300	Glyma.14G197400	0.07	0.16
142	Glyma.02G230400	Glyma.14G197500	0.06	0.13
143	Glyma.02G230500	Glyma.14G197600	0.01	0.12
144	Glyma.02G230600	Glyma.14G197700	0.09	0.17
145	Glyma.02G230700	Glyma.14G197800	0.05	0.11
146	Glyma.02G230800	Glyma.14G197900	0.07	0.17
147	Glyma.02G230900	Glyma.14G198000	0.42	1.63
148	Glyma.02G231100	Glyma.14G198700	0.03	0.09
149	Glyma.02G231200	Glyma.14G198800	0.02	0.21
150	Glyma.02G231600	Glyma.14G198900	0.01	0.20
151	Glyma.02G231800	Glyma.14G199000	0.03	0.17
152	Glyma.02G232100	Glyma.14G199200	0.03	0.21
153	Glyma.02G232500	Glyma.14G199900	0.11	0.25
154	Glyma.02G232600	Glyma.14G200200	0.02	0.12
155	Glyma.02G232700	Glyma.14G200300	0.07	0.21
156	Glyma.02G232800	Glyma.14G200400	0.01	0.10

157	Glyma.02G233000	Glyma.14G200600	0.03	0.11
158	Glyma.02G233100	Glyma.14G200700	0.05	0.15
159	Glyma.02G233200	Glyma.14G201200	0.05	0.16
160	Glyma.02G233300	Glyma.14G201300	0.04	0.13
161	Glyma.02G233700	Glyma.14G201500	0.02	0.10
162	Glyma.02G233800	Glyma.14G201600	0.03	0.15
163	Glyma.02G233900	Glyma.14G201700	0.18	0.72
164	Glyma.02G234200	Glyma.14G201800	0.03	0.10
165	Glyma.02G234300	Glyma.14G202000	0.04	0.16
166	Glyma.02G234500	Glyma.14G202100	0.03	0.18
167	Glyma.02G234600	Glyma.14G202300	0.03	0.09
168	Glyma.02G234800	Glyma.14G202700	0.07	0.29
169	Glyma.02G234900	Glyma.14G202800	0.09	0.15
170	Glyma.02G235000	Glyma.14G202900	0.23	0.38
171	Glyma.02G235100	Glyma.14G203000	0.02	0.20
172	Glyma.02G235400	Glyma.14G203400	0.04	0.13
173	Glyma.02G235500	Glyma.14G203500	0.05	0.24
174	Glyma.02G235600	Glyma.14G203600	0.04	0.13
175	Glyma.02G235700	Glyma.14G203700	0.02	0.12
176	Glyma.02G235800	Glyma.14G203800	0.04	0.09
177	Glyma.02G235900	Glyma.14G203900	0.01	0.10
178	Glyma.02G236100	Glyma.14G204000	0.09	0.19
179	Glyma.02G236200	Glyma.14G204100	0.03	0.15
180	Glyma.02G236300	Glyma.14G204200	0.03	0.17
181	Glyma.02G236400	Glyma.14G204400	0.05	0.12
182	Glyma.02G236600	Glyma.14G205100	0.03	0.12
183	Glyma.02G236700	Glyma.14G205500	0.01	0.06
184	Glyma.02G236800	Glyma.14G205600	0.05	0.23
185	Glyma.02G236900	Glyma.14G205700	0.00	0.14
186	Glyma.02G237000	Glyma.14G206000	0.03	0.18
187	Glyma.02G237100	Glyma.14G206100	0.03	0.15
188	Glyma.02G237200	Glyma.14G206200	0.14	0.28
189	Glyma.02G237400	Glyma.14G206500	0.23	0.28
190	Glyma.02G237600	Glyma.14G206600	0.10	0.16
191	Glyma.02G237700	Glyma.14G206700	0.10	0.34
192	Glyma.02G237800	Glyma.14G206800	0.03	0.11
193	Glyma.02G238300	Glyma.14G207200	0.01	0.13
194	Glyma.02G238400	Glyma.14G207300	0.01	0.07

195	Glyma.02G238500	Glyma.14G207400	0.06	0.15
196	Glyma.02G238600	Glyma.14G207500	0.03	0.15
197	Glyma.02G238700	Glyma.14G207600	0.02	0.16
198	Glyma.02G238800	Glyma.14G207700	0.02	0.21
199	Glyma.02G239000	Glyma.14G207900	0.05	0.14
200	Glyma.02G239100	Glyma.14G208000	0.02	0.15
201	Glyma.02G239200	Glyma.14G208100	0.04	0.08
202	Glyma.02G239300	Glyma.14G208200	0.04	0.31
203	Glyma.02G239600	Glyma.14G208500	0.01	0.11
204	Glyma.02G239700	Glyma.14G208600	0.00	0.18
205	Glyma.02G239800	Glyma.14G209000	0.07	0.11
206	Glyma.02G239900	Glyma.14G209200	0.08	0.13
207	Glyma.02G240000	Glyma.14G209400	0.03	0.13
208	Glyma.02G240200	Glyma.14G209700	0.06	0.15
209	Glyma.02G240300	Glyma.14G209800	0.06	0.15
210	Glyma.02G240400	Glyma.14G209900	0.01	0.10
211	Glyma.02G240500	Glyma.14G210000	0.03	0.14
212	Glyma.02G240600	Glyma.14G210100	0.01	0.15
213	Glyma.02G240700	Glyma.14G210200	0.02	0.09
214	Glyma.02G240800	Glyma.14G210400	0.02	0.15
215	Glyma.02G240900	Glyma.14G210500	0.02	0.19
216	Glyma.02G241000	Glyma.14G210600	0.05	0.13
217	Glyma.02G241100	Glyma.14G210700	0.03	0.14
218	Glyma.02G241200	Glyma.14G211000	0.00	0.11
219	Glyma.02G241400	Glyma.14G211100	0.02	0.12
220	Glyma.02G241500	Glyma.14G211200	0.02	0.18
221	Glyma.02G241600	Glyma.14G211300	0.04	0.10
222	Glyma.02G241700	Glyma.14G211400	0.06	0.09
223	Glyma.02G241800	Glyma.14G211500	0.03	0.11
224	Glyma.02G241900	Glyma.14G211600	0.02	0.12
225	Glyma.02G242000	Glyma.14G211800	0.02	0.09
226	Glyma.02G242100	Glyma.14G211900	0.05	0.14
227	Glyma.02G242400	Glyma.14G212000	0.02	0.12
228	Glyma.02G242700	Glyma.14G212100	0.08	0.22
229	Glyma.02G242900	Glyma.14G212200	0.04	0.17
230	Glyma.02G243200	Glyma.14G212300	0.04	0.10
231	Glyma.02G243300	Glyma.14G212400	0.08	0.13
232	Glyma.02G243400	Glyma.14G212500	0.02	0.10

233	Glyma.02G243500	Glyma.14G212600	0.05	0.12
234	Glyma.02G243600	Glyma.14G212700	0.04	0.21
235	Glyma.02G243800	Glyma.14G212900	0.04	0.08
236	Glyma.02G243900	Glyma.14G213200	1.11	1.23
237	Glyma.02G244100	Glyma.14G213400	0.02	0.09
238	Glyma.02G244200	Glyma.14G213600	0.04	0.19
239	Glyma.02G244700	Glyma.14G214300	0.03	0.17
240	Glyma.02G245000	Glyma.14G215100	0.04	0.21
241	Glyma.02G245200	Glyma.14G215300	0.04	0.12
242	Glyma.02G245300	Glyma.14G215400	0.02	0.05
243	Glyma.02G245400	Glyma.14G215500	0.02	0.11
244	Glyma.02G245600	Glyma.14G215600	0.01	0.08
245	Glyma.02G245700	Glyma.14G215800	0.03	0.21
246	Glyma.02G245800	Glyma.14G215900	0.03	0.08
247	Glyma.02G245900	Glyma.14G216000	0.03	0.11
248	Glyma.02G246000	Glyma.14G216100	0.03	0.12

[40] Glyma.02G227200 is contained in a huge block (Score 5176.0, *E*-value 1e-98) with 133 anchors

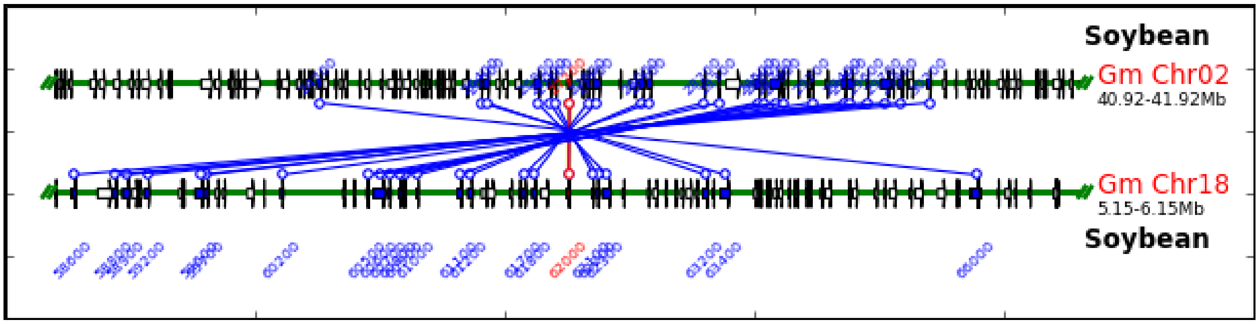

Order within Block	Locus 1	Locus 2	Ka	Ks
1	Glyma.02G224200	Glyma.18G066000	0.67	2.48
2	Glyma.02G226400	Glyma.18G063400	0.15	0.70
3	Glyma.02G226500	Glyma.18G063200	0.15	0.49
4	Glyma.02G226900	Glyma.18G062300	0.19	0.62
5	Glyma.02G227000	Glyma.18G062200	0.19	0.56
6	Glyma.02G227100	Glyma.18G062100	0.31	0.49
7	Glyma.02G227200	Glyma.18G062000	0.15	0.78
8	Glyma.02G227500	Glyma.18G061800	0.16	0.47
9	Glyma.02G227700	Glyma.18G061700	0.12	0.59

10	Glyma.02G228000	Glyma.18G061200	0.13	0.63
11	Glyma.02G228100	Glyma.18G061100	0.06	0.56
12	Glyma.02G228200	Glyma.18G061000	0.14	0.67
13	Glyma.02G228300	Glyma.18G060900	0.32	0.70
14	Glyma.02G228500	Glyma.18G060800	0.17	0.44
15	Glyma.02G228600	Glyma.18G060700	0.16	0.50
16	Glyma.02G228700	Glyma.18G060600	0.11	0.46
17	Glyma.02G228800	Glyma.18G060500	0.30	0.90
18	Glyma.02G229200	Glyma.18G060200	0.12	0.48
19	Glyma.02G229400	Glyma.18G059700	0.12	0.56
20	Glyma.02G229500	Glyma.18G059600	0.19	0.61
21	Glyma.02G229600	Glyma.18G059200	0.28	0.80
22	Glyma.02G229700	Glyma.18G058900	0.08	0.53
23	Glyma.02G229900	Glyma.18G058800	0.14	0.68
24	Glyma.02G230200	Glyma.18G058600	0.19	1.19
25	Glyma.02G230600	Glyma.18G058200	0.14	0.50
26	Glyma.02G230900	Glyma.18G057800	0.37	0.86
27	Glyma.02G231600	Glyma.18G057200	0.10	0.57
28	Glyma.02G232500	Glyma.18G057000	0.22	1.15
29	Glyma.02G232600	Glyma.18G056600	0.19	0.70
30	Glyma.02G233200	Glyma.18G055900	0.14	0.51
31	Glyma.02G233300	Glyma.18G055800	0.29	0.73
32	Glyma.02G233700	Glyma.18G055700	0.13	0.53
33	Glyma.02G233800	Glyma.18G055600	0.12	0.60
34	Glyma.02G233900	Glyma.18G055400	0.15	0.70
35	Glyma.02G234200	Glyma.18G055300	0.19	0.56
36	Glyma.02G234300	Glyma.18G055200	0.16	0.46
37	Glyma.02G234800	Glyma.18G055000	0.14	0.57
38	Glyma.02G234900	Glyma.18G054800	0.26	1.34
39	Glyma.02G235100	Glyma.18G054600	0.26	1.07
40	Glyma.02G235500	Glyma.18G054300	0.31	0.81
41	Glyma.02G235600	Glyma.18G054200	0.14	0.64
42	Glyma.02G235700	Glyma.18G054100	0.16	0.57
43	Glyma.02G235900	Glyma.18G054000	0.07	0.61
44	Glyma.02G236200	Glyma.18G052500	0.14	0.66
45	Glyma.02G236300	Glyma.18G052300	0.33	0.73
46	Glyma.02G236400	Glyma.18G052100	0.22	0.60
47	Glyma.02G236600	Glyma.18G051600	0.25	0.78

48	Glyma.02G236800	Glyma.18G051500	0.38	0.96
49	Glyma.02G236900	Glyma.18G051400	0.01	0.57
50	Glyma.02G237000	Glyma.18G051100	0.11	0.57
51	Glyma.02G237100	Glyma.18G050800	0.16	0.85
52	Glyma.02G237200	Glyma.18G050700	0.20	0.52
53	Glyma.02G237600	Glyma.18G050400	0.10	0.52
54	Glyma.02G237800	Glyma.18G050300	0.12	0.47
55	Glyma.02G237900	Glyma.18G050200	0.24	0.59
56	Glyma.02G238500	Glyma.18G048800	0.19	0.82
57	Glyma.02G238600	Glyma.18G048700	0.13	0.46
58	Glyma.02G239000	Glyma.18G048000	0.29	0.73
59	Glyma.02G239100	Glyma.18G047800	0.19	0.78
60	Glyma.02G239600	Glyma.18G046800	0.06	0.48
61	Glyma.02G239800	Glyma.18G045200	0.25	0.63
62	Glyma.02G240000	Glyma.18G045100	0.11	0.51
63	Glyma.02G241000	Glyma.18G044200	0.24	0.63
64	Glyma.02G241400	Glyma.18G043600	0.06	0.68
65	Glyma.02G241500	Glyma.18G043500	0.16	0.82
66	Glyma.02G242100	Glyma.18G043000	0.21	0.69
67	Glyma.02G242200	Glyma.18G042900	0.29	0.64
68	Glyma.02G242400	Glyma.18G042300	0.13	0.44
69	Glyma.02G242700	Glyma.18G042200	0.26	1.07
70	Glyma.02G242900	Glyma.18G042100	0.24	0.99
71	Glyma.02G243600	Glyma.18G041400	0.16	0.85
72	Glyma.02G243900	Glyma.18G041100	0.10	0.62
73	Glyma.02G244100	Glyma.18G040900	0.08	0.51
74	Glyma.02G244200	Glyma.18G040800	0.22	0.90
75	Glyma.02G244600	Glyma.18G040700	0.14	0.47
76	Glyma.02G244700	Glyma.18G040600	0.10	0.52
77	Glyma.02G245000	Glyma.18G040200	0.23	1.19
78	Glyma.02G245700	Glyma.18G039500	0.12	0.62
79	Glyma.02G246000	Glyma.18G039400	0.15	0.60
80	Glyma.02G246400	Glyma.18G039200	0.15	0.48
81	Glyma.02G246500	Glyma.18G039100	0.10	0.58
82	Glyma.02G246800	Glyma.18G038900	0.40	0.82
83	Glyma.02G247000	Glyma.18G038700	0.20	0.80
84	Glyma.02G247600	Glyma.18G038500	0.15	0.49
85	Glyma.02G247900	Glyma.18G038400	0.36	0.91

86	Glyma.02G248400	Glyma.18G038100	0.39	1.23
87	Glyma.02G248500	Glyma.18G038000	0.10	0.77
88	Glyma.02G248700	Glyma.18G037700	0.06	0.48
89	Glyma.02G248900	Glyma.18G037400	0.23	0.62
90	Glyma.02G249000	Glyma.18G037100	0.29	0.68
91	Glyma.02G249100	Glyma.18G037000	0.38	1.28
92	Glyma.02G249400	Glyma.18G036800	0.18	0.76
93	Glyma.02G249500	Glyma.18G036700	0.24	0.82
94	Glyma.02G249600	Glyma.18G036400	0.05	0.32
95	Glyma.02G249800	Glyma.18G036200	0.18	1.02
96	Glyma.02G250100	Glyma.18G036000	0.37	0.73
97	Glyma.02G250200	Glyma.18G035000	0.20	0.88
98	Glyma.02G250700	Glyma.18G034600	0.09	0.44
99	Glyma.02G250800	Glyma.18G034400	0.04	0.52
100	Glyma.02G251100	Glyma.18G034300	0.07	0.57
101	Glyma.02G251400	Glyma.18G034200	0.11	0.40
102	Glyma.02G251800	Glyma.18G034100	0.14	0.53
103	Glyma.02G252000	Glyma.18G033600	0.06	0.66
104	Glyma.02G252200	Glyma.18G033500	0.40	1.57
105	Glyma.02G252500	Glyma.18G033300	0.80	1.00
106	Glyma.02G252600	Glyma.18G032100	0.11	0.58
107	Glyma.02G252700	Glyma.18G031800	0.28	1.18
108	Glyma.02G252900	Glyma.18G031600	0.13	0.82
109	Glyma.02G253200	Glyma.18G031300	0.12	0.62
110	Glyma.02G253300	Glyma.18G031200	0.08	0.60
111	Glyma.02G253600	Glyma.18G031100	0.06	0.62
112	Glyma.02G253700	Glyma.18G030900	0.17	0.84
113	Glyma.02G253900	Glyma.18G030500	0.23	0.51
114	Glyma.02G254200	Glyma.18G030300	0.15	0.66
115	Glyma.02G254300	Glyma.18G030200	0.09	0.75
116	Glyma.02G254600	Glyma.18G029900	0.25	0.95
117	Glyma.02G254800	Glyma.18G029700	0.18	0.90
118	Glyma.02G254900	Glyma.18G029500	0.06	0.61
119	Glyma.02G255000	Glyma.18G029400	0.13	0.79
120	Glyma.02G255400	Glyma.18G029300	0.25	0.87
121	Glyma.02G255500	Glyma.18G029100	0.08	0.69
122	Glyma.02G255800	Glyma.18G029000	0.07	0.66
123	Glyma.02G256600	Glyma.18G028700	0.18	0.47

124	Glyma.02G256700	Glyma.18G028500	0.10	0.35
125	Glyma.02G256800	Glyma.18G028300	0.10	0.71
126	Glyma.02G256900	Glyma.18G028100	0.22	0.77
127	Glyma.02G257000	Glyma.18G027200	0.24	0.86
128	Glyma.02G257100	Glyma.18G027100	0.34	1.56
129	Glyma.02G257300	Glyma.18G026700	0.16	0.64
130	Glyma.02G257700	Glyma.18G026500	0.12	0.70
131	Glyma.02G258000	Glyma.18G026200	0.12	0.69
132	Glyma.02G258200	Glyma.18G026000	0.01	0.45
133	Glyma.02G258300	Glyma.18G025800	0.18	0.42

FAD3C (locus Glyma.18g062000)

Syntenic blocks: 53 (27 plant species)
Gene anchors: 6 - 302

[39] Glyma.18G062000 is contained in a huge block (Score 5176.0, *E*-value 1e-98) with 133 anchors

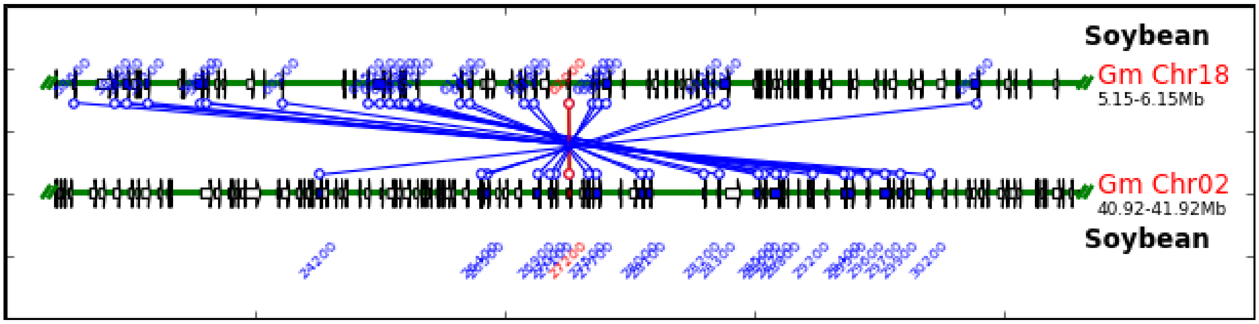

Order within Block	Locus 1	Locus 2	Ka	Ks
1	Glyma.02G224200	Glyma.18G066000	0.67	2.48
2	Glyma.02G226400	Glyma.18G063400	0.15	0.70
3	Glyma.02G226500	Glyma.18G063200	0.15	0.49
4	Glyma.02G226900	Glyma.18G062300	0.19	0.62
5	Glyma.02G227000	Glyma.18G062200	0.19	0.56
6	Glyma.02G227100	Glyma.18G062100	0.31	0.49
7	Glyma.02G227200	Glyma.18G062000	0.15	0.78
8	Glyma.02G227500	Glyma.18G061800	0.16	0.47
9	Glyma.02G227700	Glyma.18G061700	0.12	0.59
10	Glyma.02G228000	Glyma.18G061200	0.13	0.63
11	Glyma.02G228100	Glyma.18G061100	0.06	0.56
12	Glyma.02G228200	Glyma.18G061000	0.14	0.67
13	Glyma.02G228300	Glyma.18G060900	0.32	0.70
14	Glyma.02G228500	Glyma.18G060800	0.17	0.44
15	Glyma.02G228600	Glyma.18G060700	0.16	0.50
16	Glyma.02G228700	Glyma.18G060600	0.11	0.46
17	Glyma.02G228800	Glyma.18G060500	0.30	0.90
18	Glyma.02G229200	Glyma.18G060200	0.12	0.48
19	Glyma.02G229400	Glyma.18G059700	0.12	0.56
20	Glyma.02G229500	Glyma.18G059600	0.19	0.61
21	Glyma.02G229600	Glyma.18G059200	0.28	0.80
22	Glyma.02G229700	Glyma.18G058900	0.08	0.53

23	Glyma.02G229900	Glyma.18G058800	0.14	0.68
24	Glyma.02G230200	Glyma.18G058600	0.19	1.19
25	Glyma.02G230600	Glyma.18G058200	0.14	0.50
26	Glyma.02G230900	Glyma.18G057800	0.37	0.86
27	Glyma.02G231600	Glyma.18G057200	0.10	0.57
28	Glyma.02G232500	Glyma.18G057000	0.22	1.15
29	Glyma.02G232600	Glyma.18G056600	0.19	0.70
30	Glyma.02G233200	Glyma.18G055900	0.14	0.51
31	Glyma.02G233300	Glyma.18G055800	0.29	0.73
32	Glyma.02G233700	Glyma.18G055700	0.13	0.53
33	Glyma.02G233800	Glyma.18G055600	0.12	0.60
34	Glyma.02G233900	Glyma.18G055400	0.15	0.70
35	Glyma.02G234200	Glyma.18G055300	0.19	0.56
36	Glyma.02G234300	Glyma.18G055200	0.16	0.46
37	Glyma.02G234800	Glyma.18G055000	0.14	0.57
38	Glyma.02G234900	Glyma.18G054800	0.26	1.34
39	Glyma.02G235100	Glyma.18G054600	0.26	1.07
40	Glyma.02G235500	Glyma.18G054300	0.31	0.81
41	Glyma.02G235600	Glyma.18G054200	0.14	0.64
42	Glyma.02G235700	Glyma.18G054100	0.16	0.57
43	Glyma.02G235900	Glyma.18G054000	0.07	0.61
44	Glyma.02G236200	Glyma.18G052500	0.14	0.66
45	Glyma.02G236300	Glyma.18G052300	0.33	0.73
46	Glyma.02G236400	Glyma.18G052100	0.22	0.60
47	Glyma.02G236600	Glyma.18G051600	0.25	0.78
48	Glyma.02G236800	Glyma.18G051500	0.38	0.96
49	Glyma.02G236900	Glyma.18G051400	0.01	0.57
50	Glyma.02G237000	Glyma.18G051100	0.11	0.57
51	Glyma.02G237100	Glyma.18G050800	0.16	0.85
52	Glyma.02G237200	Glyma.18G050700	0.20	0.52
53	Glyma.02G237600	Glyma.18G050400	0.10	0.52
54	Glyma.02G237800	Glyma.18G050300	0.12	0.47
55	Glyma.02G237900	Glyma.18G050200	0.24	0.59
56	Glyma.02G238500	Glyma.18G048800	0.19	0.82
57	Glyma.02G238600	Glyma.18G048700	0.13	0.46
58	Glyma.02G239000	Glyma.18G048000	0.29	0.73
59	Glyma.02G239100	Glyma.18G047800	0.19	0.78
60	Glyma.02G239600	Glyma.18G046800	0.06	0.48

61	Glyma.02G239800	Glyma.18G045200	0.25	0.63
62	Glyma.02G240000	Glyma.18G045100	0.11	0.51
63	Glyma.02G241000	Glyma.18G044200	0.24	0.63
64	Glyma.02G241400	Glyma.18G043600	0.06	0.68
65	Glyma.02G241500	Glyma.18G043500	0.16	0.82
66	Glyma.02G242100	Glyma.18G043000	0.21	0.69
67	Glyma.02G242200	Glyma.18G042900	0.29	0.64
68	Glyma.02G242400	Glyma.18G042300	0.13	0.44
69	Glyma.02G242700	Glyma.18G042200	0.26	1.07
70	Glyma.02G242900	Glyma.18G042100	0.24	0.99
71	Glyma.02G243600	Glyma.18G041400	0.16	0.85
72	Glyma.02G243900	Glyma.18G041100	0.10	0.62
73	Glyma.02G244100	Glyma.18G040900	0.08	0.51
74	Glyma.02G244200	Glyma.18G040800	0.22	0.90
75	Glyma.02G244600	Glyma.18G040700	0.14	0.47
76	Glyma.02G244700	Glyma.18G040600	0.10	0.52
77	Glyma.02G245000	Glyma.18G040200	0.23	1.19
78	Glyma.02G245700	Glyma.18G039500	0.12	0.62
79	Glyma.02G246000	Glyma.18G039400	0.15	0.60
80	Glyma.02G246400	Glyma.18G039200	0.15	0.48
81	Glyma.02G246500	Glyma.18G039100	0.10	0.58
82	Glyma.02G246800	Glyma.18G038900	0.40	0.82
83	Glyma.02G247000	Glyma.18G038700	0.20	0.80
84	Glyma.02G247600	Glyma.18G038500	0.15	0.49
85	Glyma.02G247900	Glyma.18G038400	0.36	0.91
86	Glyma.02G248400	Glyma.18G038100	0.39	1.23
87	Glyma.02G248500	Glyma.18G038000	0.10	0.77
88	Glyma.02G248700	Glyma.18G037700	0.06	0.48
89	Glyma.02G248900	Glyma.18G037400	0.23	0.62
90	Glyma.02G249000	Glyma.18G037100	0.29	0.68
91	Glyma.02G249100	Glyma.18G037000	0.38	1.28
92	Glyma.02G249400	Glyma.18G036800	0.18	0.76
93	Glyma.02G249500	Glyma.18G036700	0.24	0.82
94	Glyma.02G249600	Glyma.18G036400	0.05	0.32
95	Glyma.02G249800	Glyma.18G036200	0.18	1.02
96	Glyma.02G250100	Glyma.18G036000	0.37	0.73
97	Glyma.02G250200	Glyma.18G035000	0.20	0.88
98	Glyma.02G250700	Glyma.18G034600	0.09	0.44

99	Glyma.02G250800	Glyma.18G034400	0.04	0.52
100	Glyma.02G251100	Glyma.18G034300	0.07	0.57
101	Glyma.02G251400	Glyma.18G034200	0.11	0.40
102	Glyma.02G251800	Glyma.18G034100	0.14	0.53
103	Glyma.02G252000	Glyma.18G033600	0.06	0.66
104	Glyma.02G252200	Glyma.18G033500	0.40	1.57
105	Glyma.02G252500	Glyma.18G033300	0.80	1.00
106	Glyma.02G252600	Glyma.18G032100	0.11	0.58
107	Glyma.02G252700	Glyma.18G031800	0.28	1.18
108	Glyma.02G252900	Glyma.18G031600	0.13	0.82
109	Glyma.02G253200	Glyma.18G031300	0.12	0.62
110	Glyma.02G253300	Glyma.18G031200	0.08	0.60
111	Glyma.02G253600	Glyma.18G031100	0.06	0.62
112	Glyma.02G253700	Glyma.18G030900	0.17	0.84
113	Glyma.02G253900	Glyma.18G030500	0.23	0.51
114	Glyma.02G254200	Glyma.18G030300	0.15	0.66
115	Glyma.02G254300	Glyma.18G030200	0.09	0.75
116	Glyma.02G254600	Glyma.18G029900	0.25	0.95
117	Glyma.02G254800	Glyma.18G029700	0.18	0.90
118	Glyma.02G254900	Glyma.18G029500	0.06	0.61
119	Glyma.02G255000	Glyma.18G029400	0.13	0.79
120	Glyma.02G255400	Glyma.18G029300	0.25	0.87
121	Glyma.02G255500	Glyma.18G029100	0.08	0.69
122	Glyma.02G255800	Glyma.18G029000	0.07	0.66
123	Glyma.02G256600	Glyma.18G028700	0.18	0.47
124	Glyma.02G256700	Glyma.18G028500	0.10	0.35
125	Glyma.02G256800	Glyma.18G028300	0.10	0.71
126	Glyma.02G256900	Glyma.18G028100	0.22	0.77
127	Glyma.02G257000	Glyma.18G027200	0.24	0.86
128	Glyma.02G257100	Glyma.18G027100	0.34	1.56
129	Glyma.02G257300	Glyma.18G026700	0.16	0.64
130	Glyma.02G257700	Glyma.18G026500	0.12	0.70
131	Glyma.02G258000	Glyma.18G026200	0.12	0.69
132	Glyma.02G258200	Glyma.18G026000	0.01	0.45
133	Glyma.02G258300	Glyma.18G025800	0.18	0.42

[41] Glyma.18G062000 is contained in a huge block (Score 2991.0, *E*-value 1e-43) with 76 anchors

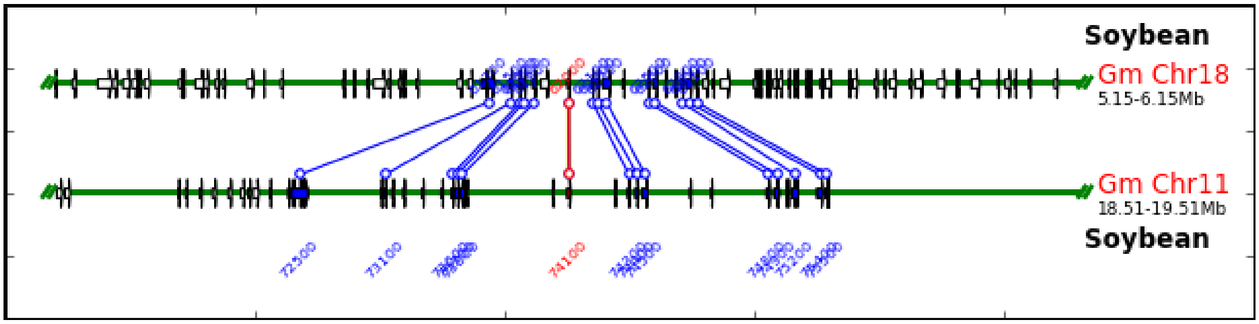

Order within Block	Locus 1	Locus 2	Ka	Ks
1	Glyma.11G159700	Glyma.18G053500	0.34	0.78
2	Glyma.11G160100	Glyma.18G053900	0.01	0.10
3	Glyma.11G160400	Glyma.18G054400	0.01	0.14
4	Glyma.11G160600	Glyma.18G054500	0.05	0.12
5	Glyma.11G160900	Glyma.18G054700	0.06	0.10
6	Glyma.11G161000	Glyma.18G054800	0.03	0.13
7	Glyma.11G161200	Glyma.18G054900	0.05	0.14
8	Glyma.11G161300	Glyma.18G055000	0.02	0.13
9	Glyma.11G161400	Glyma.18G055100	0.03	0.10
10	Glyma.11G161500	Glyma.18G055200	0.02	0.09
11	Glyma.11G161600	Glyma.18G055300	0.02	0.15
12	Glyma.11G161800	Glyma.18G055400	0.03	0.18
13	Glyma.11G162100	Glyma.18G055600	0.02	0.11
14	Glyma.11G162400	Glyma.18G055700	0.08	0.08
15	Glyma.11G162500	Glyma.18G055800	0.16	0.25
16	Glyma.11G162700	Glyma.18G056000	0.62	0.91
17	Glyma.11G162800	Glyma.18G056100	0.45	0.78
18	Glyma.11G162900	Glyma.18G056200	0.06	0.15
19	Glyma.11G163000	Glyma.18G056300	0.02	0.10
20	Glyma.11G163100	Glyma.18G056400	0.06	0.16
21	Glyma.11G163300	Glyma.18G056600	0.04	0.13
22	Glyma.11G163400	Glyma.18G056700	0.17	0.32
23	Glyma.11G163500	Glyma.18G056800	0.28	0.34
24	Glyma.11G163800	Glyma.18G057000	0.04	0.13
25	Glyma.11G163900	Glyma.18G057100	0.02	0.07
26	Glyma.11G164000	Glyma.18G057200	0.01	0.12
27	Glyma.11G164100	Glyma.18G057300	0.02	0.13
28	Glyma.11G164300	Glyma.18G057500	0.06	0.12

29	Glyma.11G164400	Glyma.18G057600	0.11	0.46
30	Glyma.11G164500	Glyma.18G057700	0.03	0.10
31	Glyma.11G164600	Glyma.18G057800	0.09	0.16
32	Glyma.11G164700	Glyma.18G057900	0.02	0.19
33	Glyma.11G164800	Glyma.18G058000	0.02	0.14
34	Glyma.11G165000	Glyma.18G058200	0.05	0.17
35	Glyma.11G165100	Glyma.18G058300	0.03	0.13
36	Glyma.11G165300	Glyma.18G058400	0.07	0.12
37	Glyma.11G165600	Glyma.18G058500	0.06	0.07
38	Glyma.11G165700	Glyma.18G058600	0.08	0.23
39	Glyma.11G165900	Glyma.18G058700	0.03	0.09
40	Glyma.11G166000	Glyma.18G058800	0.04	0.15
41	Glyma.11G166100	Glyma.18G058900	0.02	0.09
42	Glyma.11G166200	Glyma.18G059000	0.06	0.11
43	Glyma.11G166300	Glyma.18G059200	0.05	0.13
44	Glyma.11G166500	Glyma.18G059300	0.02	0.17
45	Glyma.11G166600	Glyma.18G059400	0.31	0.48
46	Glyma.11G167100	Glyma.18G059600	0.04	0.13
47	Glyma.11G167200	Glyma.18G059700	0.03	0.13
48	Glyma.11G167300	Glyma.18G059800	0.03	0.18
49	Glyma.11G168600	Glyma.18G060000	0.06	0.21
50	Glyma.11G168800	Glyma.18G060200	0.02	0.05
51	Glyma.11G169300	Glyma.18G060300	0.03	0.26
52	Glyma.11G169500	Glyma.18G060400	0.06	0.27
53	Glyma.11G169600	Glyma.18G060500	0.08	0.23
54	Glyma.11G169700	Glyma.18G060600	0.01	0.09
55	Glyma.11G169800	Glyma.18G060700	0.05	0.14
56	Glyma.11G169900	Glyma.18G060800	0.05	0.12
57	Glyma.11G170000	Glyma.18G060900	0.08	0.17
58	Glyma.11G170200	Glyma.18G061000	0.03	0.16
59	Glyma.11G170300	Glyma.18G061100	0.01	0.13
60	Glyma.11G170500	Glyma.18G061200	0.06	0.15
61	Glyma.11G172500	Glyma.18G061300	0.05	0.18
62	Glyma.11G173100	Glyma.18G061500	0.00	0.09
63	Glyma.11G173600	Glyma.18G061600	0.08	0.08
64	Glyma.11G173700	Glyma.18G061700	0.01	0.11
65	Glyma.11G173800	Glyma.18G061800	0.02	0.10
66	Glyma.11G174100	Glyma.18G062000	0.02	0.12

67	Glyma.11G174300	Glyma.18G062100	0.04	0.12
68	Glyma.11G174400	Glyma.18G062200	0.03	0.16
69	Glyma.11G174500	Glyma.18G062300	0.05	0.13
70	Glyma.11G174800	Glyma.18G062500	0.04	0.16
71	Glyma.11G174900	Glyma.18G062600	0.03	0.14
72	Glyma.11G175200	Glyma.18G062900	0.01	0.12
73	Glyma.11G175400	Glyma.18G063000	0.04	0.11
74	Glyma.11G175500	Glyma.18G063100	0.01	0.08
75	Glyma.11G178700	Glyma.18G063200	0.05	0.13
76	Glyma.11G178800	Glyma.18G063300	0.05	0.13

[42] Glyma.18G062000 is contained in a huge block (Score 3013.0, *E*-value 1e-101) with 78 anchors

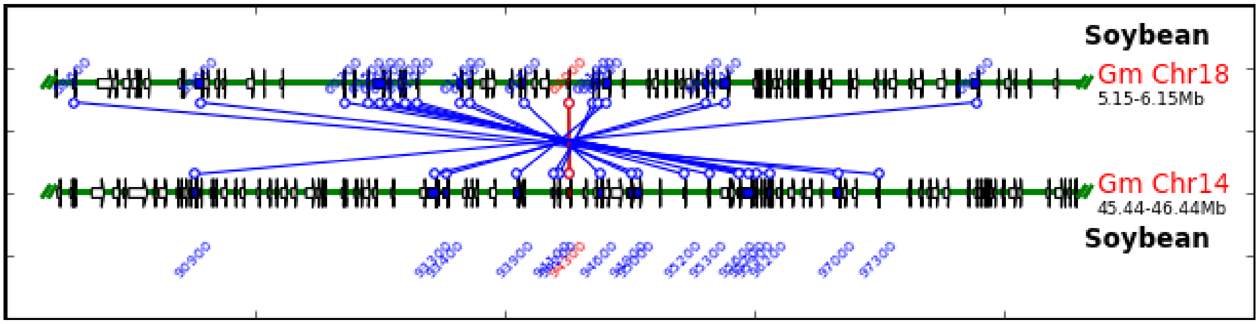

Order within Block	Locus 1	Locus 2	Ka	Ks
1	Glyma.14G190900	Glyma.18G066000	0.67	2.15
2	Glyma.14G193300	Glyma.18G063400	0.17	0.79
3	Glyma.14G193400	Glyma.18G063200	0.14	0.51
4	Glyma.14G193900	Glyma.18G062300	0.19	0.64
5	Glyma.14G194100	Glyma.18G062200	0.19	0.57
6	Glyma.14G194200	Glyma.18G062100	0.30	0.50
7	Glyma.14G194300	Glyma.18G062000	0.14	0.84
8	Glyma.14G194600	Glyma.18G061700	0.12	0.56
9	Glyma.14G194900	Glyma.18G061200	0.14	0.61
10	Glyma.14G195000	Glyma.18G061100	0.06	0.53
11	Glyma.14G195200	Glyma.18G061000	0.15	0.68
12	Glyma.14G195300	Glyma.18G060900	0.35	0.68
13	Glyma.14G195600	Glyma.18G060700	0.15	0.47
14	Glyma.14G195700	Glyma.18G060600	0.12	0.44
15	Glyma.14G195900	Glyma.18G060500	0.29	0.95

16	Glyma.14G196200	Glyma.18G060300	0.15	1.09
17	Glyma.14G197000	Glyma.18G059600	0.20	0.56
18	Glyma.14G197300	Glyma.18G058600	0.20	1.37
19	Glyma.14G197700	Glyma.18G058200	0.15	0.45
20	Glyma.14G198000	Glyma.18G057800	0.47	2.05
21	Glyma.14G198900	Glyma.18G057200	0.10	0.50
22	Glyma.14G199900	Glyma.18G057000	0.29	1.51
23	Glyma.14G200000	Glyma.18G056800	0.69	3.17
24	Glyma.14G200200	Glyma.18G056600	0.19	0.64
25	Glyma.14G200500	Glyma.18G056400	0.26	1.16
26	Glyma.14G201200	Glyma.18G055900	0.14	0.50
27	Glyma.14G201300	Glyma.18G055800	0.30	0.67
28	Glyma.14G201500	Glyma.18G055700	0.12	0.56
29	Glyma.14G201600	Glyma.18G055600	0.12	0.56
30	Glyma.14G201700	Glyma.18G055400	0.13	0.82
31	Glyma.14G201800	Glyma.18G055300	0.23	0.57
32	Glyma.14G202000	Glyma.18G055200	0.11	0.54
33	Glyma.14G202700	Glyma.18G055000	0.13	0.53
34	Glyma.14G202800	Glyma.18G054800	0.32	1.36
35	Glyma.14G203000	Glyma.18G054600	0.24	1.02
36	Glyma.14G203500	Glyma.18G054300	0.47	0.76
37	Glyma.14G203600	Glyma.18G054200	0.15	0.54
38	Glyma.14G203700	Glyma.18G054100	0.17	0.55
39	Glyma.14G203900	Glyma.18G054000	0.07	0.59
40	Glyma.14G204100	Glyma.18G052500	0.14	0.74
41	Glyma.14G204200	Glyma.18G052300	0.29	0.69
42	Glyma.14G204400	Glyma.18G052100	0.23	0.63
43	Glyma.14G205100	Glyma.18G051600	0.25	0.88
44	Glyma.14G205600	Glyma.18G051500	0.38	0.84
45	Glyma.14G205700	Glyma.18G051400	0.01	0.63
46	Glyma.14G206000	Glyma.18G051100	0.12	0.59
47	Glyma.14G206100	Glyma.18G050800	0.17	0.82
48	Glyma.14G206200	Glyma.18G050700	0.21	0.48
49	Glyma.14G206600	Glyma.18G050400	0.07	0.51
50	Glyma.14G206800	Glyma.18G050300	0.10	0.45
51	Glyma.14G207400	Glyma.18G048800	0.19	0.73
52	Glyma.14G207500	Glyma.18G048700	0.15	0.51
53	Glyma.14G207800	Glyma.18G048400	0.09	0.94

54	Glyma.14G207900	Glyma.18G048000	0.26	0.76
55	Glyma.14G208000	Glyma.18G047800	0.19	0.74
56	Glyma.14G208400	Glyma.18G047000	0.25	0.73
57	Glyma.14G208500	Glyma.18G046800	0.06	0.46
58	Glyma.14G208700	Glyma.18G045600	0.49	2.01
59	Glyma.14G208900	Glyma.18G045300	0.22	0.75
60	Glyma.14G209000	Glyma.18G045200	0.34	0.56
61	Glyma.14G209400	Glyma.18G045100	0.13	0.47
62	Glyma.14G209600	Glyma.18G044900	0.15	1.11
63	Glyma.14G210600	Glyma.18G044200	0.23	0.59
64	Glyma.14G211100	Glyma.18G043600	0.05	0.77
65	Glyma.14G211200	Glyma.18G043500	0.14	0.76
66	Glyma.14G211900	Glyma.18G043000	0.20	0.69
67	Glyma.14G212000	Glyma.18G042300	0.13	0.50
68	Glyma.14G212100	Glyma.18G042200	0.15	0.73
69	Glyma.14G212200	Glyma.18G042100	0.24	0.91
70	Glyma.14G212700	Glyma.18G041400	0.18	0.79
71	Glyma.14G213100	Glyma.18G041300	0.44	0.73
72	Glyma.14G213200	Glyma.18G041100	0.14	0.63
73	Glyma.14G213400	Glyma.18G040900	0.08	0.46
74	Glyma.14G213600	Glyma.18G040800	0.23	0.88
75	Glyma.14G214500	Glyma.18G040700	0.14	0.53
76	Glyma.14G215100	Glyma.18G040200	0.25	1.11
77	Glyma.14G215800	Glyma.18G039500	0.14	0.64
78	Glyma.14G216100	Glyma.18G039400	0.15	0.59

***FAD3D* (locus Glyma.11g174100)**

Syntenic blocks: 36 (21 plant species)
Gene anchors: 7 - 76

[27] Glyma.11G174100 is contained in a large block (Score 1688.0, *E*-value 0.0) with 44 anchors

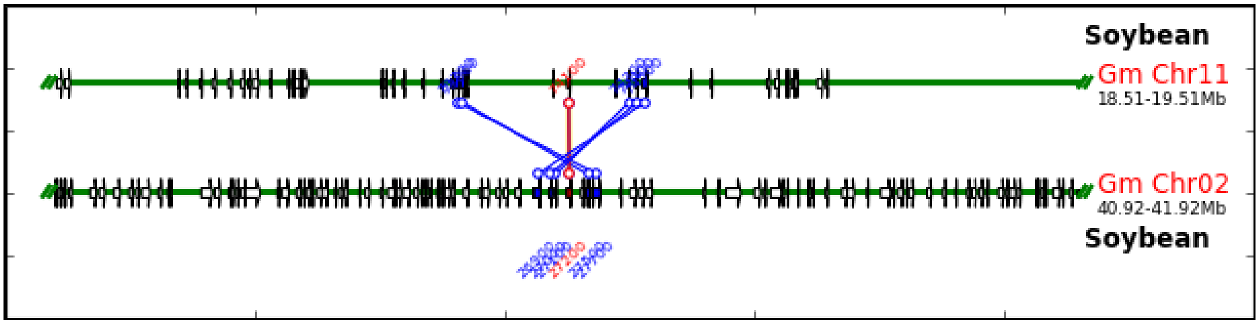

Order within Block	Locus 1	Locus 2	Ka	Ks
1	Glyma.02G224000	Glyma.11G178300	0.21	0.44
2	Glyma.02G224100	Glyma.11G177800	0.01	0.99
3	Glyma.02G224400	Glyma.11G176900	0.56	1.10
4	Glyma.02G224600	Glyma.11G176800	0.16	0.62
5	Glyma.02G224900	Glyma.11G176500	0.16	0.90
6	Glyma.02G225000	Glyma.11G176400	0.15	0.62
7	Glyma.02G225600	Glyma.11G176100	0.28	0.40
8	Glyma.02G226900	Glyma.11G174500	0.21	0.52
9	Glyma.02G227000	Glyma.11G174400	0.19	0.58
10	Glyma.02G227100	Glyma.11G174300	0.30	0.52
11	Glyma.02G227200	Glyma.11G174100	0.15	0.90
12	Glyma.02G227500	Glyma.11G173800	0.17	0.53
13	Glyma.02G227700	Glyma.11G173700	0.13	0.60
14	Glyma.02G227900	Glyma.11G170900	0.29	0.69
15	Glyma.02G228000	Glyma.11G170500	0.15	0.81
16	Glyma.02G228100	Glyma.11G170300	0.06	0.61
17	Glyma.02G228200	Glyma.11G170200	0.14	0.71
18	Glyma.02G228300	Glyma.11G170000	0.36	0.69
19	Glyma.02G228500	Glyma.11G169900	0.13	0.41
20	Glyma.02G228600	Glyma.11G169800	0.17	0.56
21	Glyma.02G228700	Glyma.11G169700	0.12	0.44
22	Glyma.02G228800	Glyma.11G169600	0.30	0.91
23	Glyma.02G229200	Glyma.11G168800	0.13	0.56

24	Glyma.02G229400	Glyma.11G167200	0.12	0.54
25	Glyma.02G229500	Glyma.11G167100	0.21	0.63
26	Glyma.02G229600	Glyma.11G166300	0.26	0.77
27	Glyma.02G229700	Glyma.11G166100	0.08	0.59
28	Glyma.02G229900	Glyma.11G166000	0.13	0.70
29	Glyma.02G230200	Glyma.11G165700	0.18	1.10
30	Glyma.02G230600	Glyma.11G165000	0.14	0.50
31	Glyma.02G230900	Glyma.11G164600	0.39	1.10
32	Glyma.02G231600	Glyma.11G164000	0.10	0.59
33	Glyma.02G232500	Glyma.11G163800	0.22	0.97
34	Glyma.02G232600	Glyma.11G163300	0.18	0.67
35	Glyma.02G233300	Glyma.11G162500	0.23	0.52
36	Glyma.02G233700	Glyma.11G162400	0.14	0.38
37	Glyma.02G233800	Glyma.11G162100	0.12	0.67
38	Glyma.02G233900	Glyma.11G161800	0.16	0.87
39	Glyma.02G234200	Glyma.11G161600	0.19	0.50
40	Glyma.02G234300	Glyma.11G161500	0.16	0.47
41	Glyma.02G234800	Glyma.11G161300	0.14	0.62
42	Glyma.02G234900	Glyma.11G161000	0.27	1.47
43	Glyma.02G235000	Glyma.11G160600	0.39	0.97
44	Glyma.02G235600	Glyma.11G159800	0.16	0.71

[28] Glyma.11G174100 is contained in a large block (Score 1483.0, *E*-value 8e-179) with 39 anchors

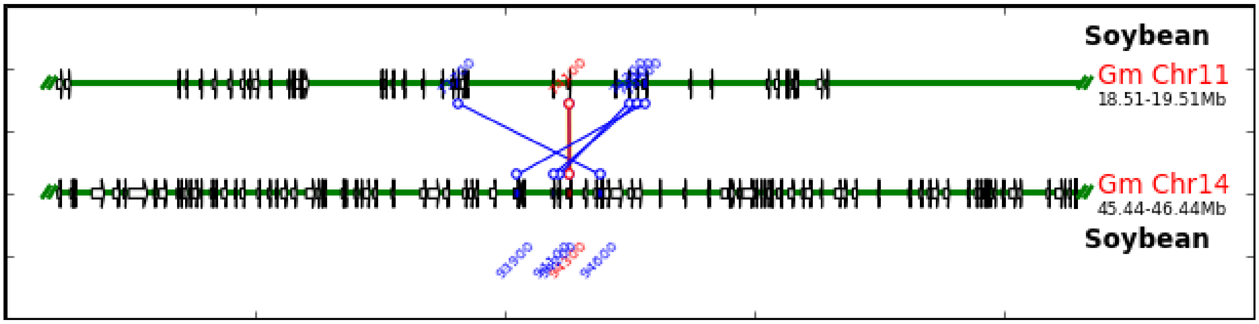

Order within Block	Locus 1	Locus 2	Ka	Ks
1	Glyma.11G159900	Glyma.14G203700	0.20	0.59
2	Glyma.11G160600	Glyma.14G202900	0.47	1.36
3	Glyma.11G161000	Glyma.14G202800	0.30	1.10
4	Glyma.11G161300	Glyma.14G202700	0.16	0.50

5	Glyma.11G161500	Glyma.14G202000	0.11	0.54
6	Glyma.11G161600	Glyma.14G201800	0.23	0.52
7	Glyma.11G161800	Glyma.14G201700	0.13	0.83
8	Glyma.11G162100	Glyma.14G201600	0.13	0.60
9	Glyma.11G162400	Glyma.14G201500	0.14	0.45
10	Glyma.11G162500	Glyma.14G201300	0.24	0.54
11	Glyma.11G163100	Glyma.14G200500	0.26	1.40
12	Glyma.11G163300	Glyma.14G200200	0.20	0.67
13	Glyma.11G163600	Glyma.14G200000	0.75	4.21
14	Glyma.11G163800	Glyma.14G199900	0.27	1.09
15	Glyma.11G164000	Glyma.14G198900	0.10	0.49
16	Glyma.11G164600	Glyma.14G198000	0.45	1.63
17	Glyma.11G165000	Glyma.14G197700	0.15	0.47
18	Glyma.11G165700	Glyma.14G197300	0.17	1.27
19	Glyma.11G169300	Glyma.14G196200	0.16	1.13
20	Glyma.11G169600	Glyma.14G195900	0.29	1.13
21	Glyma.11G169700	Glyma.14G195700	0.12	0.45
22	Glyma.11G169800	Glyma.14G195600	0.16	0.53
23	Glyma.11G170000	Glyma.14G195300	0.33	0.69
24	Glyma.11G170200	Glyma.14G195200	0.14	0.69
25	Glyma.11G170300	Glyma.14G195000	0.05	0.60
26	Glyma.11G170500	Glyma.14G194900	0.12	0.71
27	Glyma.11G173700	Glyma.14G194600	0.13	0.61
28	Glyma.11G174100	Glyma.14G194300	0.15	0.96
29	Glyma.11G174300	Glyma.14G194200	0.29	0.50
30	Glyma.11G174400	Glyma.14G194100	0.19	0.59
31	Glyma.11G174500	Glyma.14G193900	0.22	0.59
32	Glyma.11G175900	Glyma.14G192500	0.14	1.11
33	Glyma.11G176100	Glyma.14G192400	0.30	0.32
34	Glyma.11G176400	Glyma.14G191900	0.16	0.61
35	Glyma.11G176500	Glyma.14G191700	0.14	0.92
36	Glyma.11G176800	Glyma.14G191300	0.15	0.58
37	Glyma.11G176900	Glyma.14G191100	0.40	0.91
38	Glyma.11G177800	Glyma.14G190800	0.01	1.04
39	Glyma.11G178300	Glyma.14G190700	0.20	0.48

[29] Glyma.11G174100 is contained in a huge block (Score 2991.0, *E*-value 1e-43) with 76 anchors

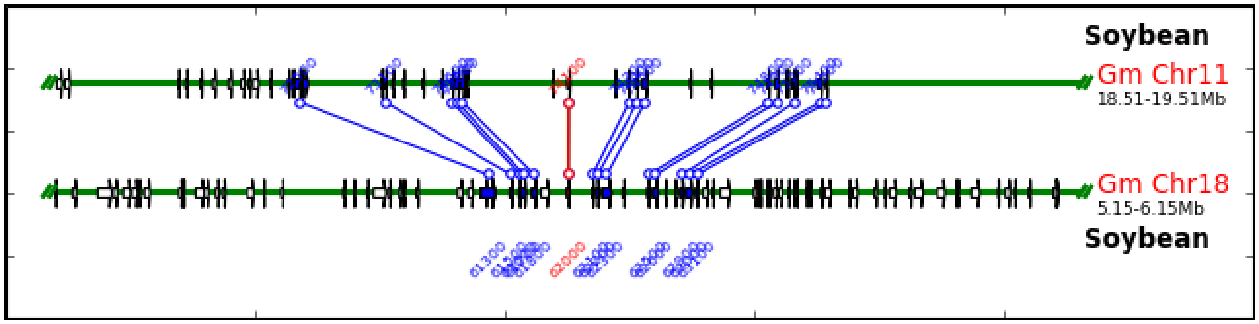

Order within Block	Locus 1	Locus 2	Ka	Ks
1	Glyma.11G159700	Glyma.18G053500	0.34	0.78
2	Glyma.11G160100	Glyma.18G053900	0.01	0.10
3	Glyma.11G160400	Glyma.18G054400	0.01	0.14
4	Glyma.11G160600	Glyma.18G054500	0.05	0.12
5	Glyma.11G160900	Glyma.18G054700	0.06	0.10
6	Glyma.11G161000	Glyma.18G054800	0.03	0.13
7	Glyma.11G161200	Glyma.18G054900	0.05	0.14
8	Glyma.11G161300	Glyma.18G055000	0.02	0.13
9	Glyma.11G161400	Glyma.18G055100	0.03	0.10
10	Glyma.11G161500	Glyma.18G055200	0.02	0.09
11	Glyma.11G161600	Glyma.18G055300	0.02	0.15
12	Glyma.11G161800	Glyma.18G055400	0.03	0.18
13	Glyma.11G162100	Glyma.18G055600	0.02	0.11
14	Glyma.11G162400	Glyma.18G055700	0.08	0.08
15	Glyma.11G162500	Glyma.18G055800	0.16	0.25
16	Glyma.11G162700	Glyma.18G056000	0.62	0.91
17	Glyma.11G162800	Glyma.18G056100	0.45	0.78
18	Glyma.11G162900	Glyma.18G056200	0.06	0.15
19	Glyma.11G163000	Glyma.18G056300	0.02	0.10
20	Glyma.11G163100	Glyma.18G056400	0.06	0.16
21	Glyma.11G163300	Glyma.18G056600	0.04	0.13
22	Glyma.11G163400	Glyma.18G056700	0.17	0.32
23	Glyma.11G163500	Glyma.18G056800	0.28	0.34
24	Glyma.11G163800	Glyma.18G057000	0.04	0.13
25	Glyma.11G163900	Glyma.18G057100	0.02	0.07
26	Glyma.11G164000	Glyma.18G057200	0.01	0.12
27	Glyma.11G164100	Glyma.18G057300	0.02	0.13
28	Glyma.11G164300	Glyma.18G057500	0.06	0.12

29	Glyma.11G164400	Glyma.18G057600	0.11	0.46
30	Glyma.11G164500	Glyma.18G057700	0.03	0.10
31	Glyma.11G164600	Glyma.18G057800	0.09	0.16
32	Glyma.11G164700	Glyma.18G057900	0.02	0.19
33	Glyma.11G164800	Glyma.18G058000	0.02	0.14
34	Glyma.11G165000	Glyma.18G058200	0.05	0.17
35	Glyma.11G165100	Glyma.18G058300	0.03	0.13
36	Glyma.11G165300	Glyma.18G058400	0.07	0.12
37	Glyma.11G165600	Glyma.18G058500	0.06	0.07
38	Glyma.11G165700	Glyma.18G058600	0.08	0.23
39	Glyma.11G165900	Glyma.18G058700	0.03	0.09
40	Glyma.11G166000	Glyma.18G058800	0.04	0.15
41	Glyma.11G166100	Glyma.18G058900	0.02	0.09
42	Glyma.11G166200	Glyma.18G059000	0.06	0.11
43	Glyma.11G166300	Glyma.18G059200	0.05	0.13
44	Glyma.11G166500	Glyma.18G059300	0.02	0.17
45	Glyma.11G166600	Glyma.18G059400	0.31	0.48
46	Glyma.11G167100	Glyma.18G059600	0.04	0.13
47	Glyma.11G167200	Glyma.18G059700	0.03	0.13
48	Glyma.11G167300	Glyma.18G059800	0.03	0.18
49	Glyma.11G168600	Glyma.18G060000	0.06	0.21
50	Glyma.11G168800	Glyma.18G060200	0.02	0.05
51	Glyma.11G169300	Glyma.18G060300	0.03	0.26
52	Glyma.11G169500	Glyma.18G060400	0.06	0.27
53	Glyma.11G169600	Glyma.18G060500	0.08	0.23
54	Glyma.11G169700	Glyma.18G060600	0.01	0.09
55	Glyma.11G169800	Glyma.18G060700	0.05	0.14
56	Glyma.11G169900	Glyma.18G060800	0.05	0.12
57	Glyma.11G170000	Glyma.18G060900	0.08	0.17
58	Glyma.11G170200	Glyma.18G061000	0.03	0.16
59	Glyma.11G170300	Glyma.18G061100	0.01	0.13
60	Glyma.11G170500	Glyma.18G061200	0.06	0.15
61	Glyma.11G172500	Glyma.18G061300	0.05	0.18
62	Glyma.11G173100	Glyma.18G061500	0.00	0.09
63	Glyma.11G173600	Glyma.18G061600	0.08	0.08
64	Glyma.11G173700	Glyma.18G061700	0.01	0.11
65	Glyma.11G173800	Glyma.18G061800	0.02	0.10
66	Glyma.11G174100	Glyma.18G062000	0.02	0.12

67	Glyma.11G174300	Glyma.18G062100	0.04	0.12
68	Glyma.11G174400	Glyma.18G062200	0.03	0.16
69	Glyma.11G174500	Glyma.18G062300	0.05	0.13
70	Glyma.11G174800	Glyma.18G062500	0.04	0.16
71	Glyma.11G174900	Glyma.18G062600	0.03	0.14
72	Glyma.11G175200	Glyma.18G062900	0.01	0.12
73	Glyma.11G175400	Glyma.18G063000	0.04	0.11
74	Glyma.11G175500	Glyma.18G063100	0.01	0.08
75	Glyma.11G178700	Glyma.18G063200	0.05	0.13
76	Glyma.11G178800	Glyma.18G063300	0.05	0.13
